# Task‐Based Mapping of Compensatory Strategies and Movement Kinematics After Stroke: A Systematic Scoping Review

**DOI:** 10.1002/pri.70215

**Published:** 2026-04-13

**Authors:** Pedro Henrique Sousa de Andrade, Caroline Rodrigues Osawa, Maria Eduarda Salum Aveiro, Clara Rezende Rocha, Rodrigo Bazan, Luciane Aparecida Pascucci de Sande Souza, Gustavo José Luvizutto

**Affiliations:** ^1^ Department of Applied Physical Therapy Federal University of Triângulo Mineiro (UFTM) Uberaba Minas Gerais Brazil; ^2^ Department of Neuroscience and Mental Health Botucatu Medical School (UNESP) Botucatu, São Paulo Brazil

**Keywords:** compensatory strategies, kinematics, mobility, postural control, stroke rehabilitation, upper extremity

## Abstract

Although compensatory motor strategies are widely described after stroke, there is still no structured synthesis integrating their kinematic characteristics across core functional tasks. This scoping review aimed to map compensatory strategies and movement kinematics in mobility, postural control, and upper‐limb tasks after stroke. The review followed the Joanna Briggs Institute methodology and was registered on the Open Science Framework. Ten databases were searched through August 2024. Studies including individuals post‐stroke that used motion analysis to characterize compensatory strategies during sit‐to‐stand and stand‐to‐sit transfers, gait, step up/down, postural control, and upper‐limb tasks (reaching, grasping, and manipulation) were included. Compensatory strategies were defined as alternative task‐oriented motor patterns involving redistribution of movement to preserved segments or substitution of impaired motor elements to maintain functional performance. Movement kinematics comprised spatiotemporal parameters and joint‐level kinematic variables derived from motion analysis. Data were extracted and synthesized according to task category. A total of 101 studies were included. In mobility tasks, the most frequently reported compensatory strategies involved asymmetrical weight‐bearing, reduced step length and cadence, prolonged movement time, and reduced joint excursion at the hip, knee, and ankle. During postural control tasks, common findings included weight‐bearing asymmetry, increased mediolateral displacement of the center of pressure, and reduced weight‐shifting capacity. In upper‐limb tasks, compensatory patterns were characterized by prolonged movement time, reduced elbow extension, and increased trunk and shoulder contribution. In conclusion, this review provides a structured task‐based mapping of compensatory motor behavior and its associated kinematic parameters in individuals after stroke.

## 1 Introduction

Stroke is the second leading cause of death worldwide and the third leading cause of health loss among adults, considering mortality and disability (GBD 2021). Reduced cerebral blood flow after stroke causes muscle weakness (Wagner et al. 2007), impaired coordination (Belas Dos Santos et al. 2018), and abnormal synergies due to loss of essential motor components (Roh et al. 2013). These motor impairments affect movement control, limiting gait, mobility, and upper‐limb function, and negatively impact social participation and quality of life (Ezekiel et al. 2019; Martino Cinnera et al. 2020; Lieshout et al. 2020). In the late subacute and chronic stages of stroke recovery, persistent motor impairments often may contribute to motor reorganization and the emergence of compensatory behavioral strategies (Mao et al. 2018; Wolf et al. 2025). These strategies enable individuals to perform daily tasks such as reaching, grasping, manipulation (Wolf et al. 2025; Choi et al. 2023), mobility (Mao et al. 2018; Carmo et al. 2012) and postural control (Honeycutt et al. 2016; Martinez et al. 2019). These compensations typically involve alternative coordination within the more affected limb or recruitment of other body segments to complete tasks (Levin et al. 2009; Bernhardt et al. 2017; Kwakkel et al. 2023).

During acute and early subacute phases, rehabilitation primarily targets recovery of essential movement components, particularly those related to mobility (Lum et al. 2009; Donnellan et al. 2012; AVERT Trial Collaboration group 2015). In chronic phases, as recovery stabilizes and neural reorganization plateaus, compensatory synergies and movement adaptations often appear during daily activities (De Luca et al. 2019). When appropriately addressed, these strategies may support independence by facilitating personal care, leisure activities, and participation in social roles (De Luca et al. 2019; Kelly et al. 2007; Jang 2013). However, excessive reinforcement of compensatory patterns can lead to secondary consequences, including pain, reduction in range of motion (ROM) (Lewis et al. 2005) and “learned non‐use” of the more affected upper limb (Kerr et al. 2011; Pain et al. 2015), or maladaptive use of the lower limbs, reflecting inefficient motor control and altered movement coordination during task execution (Dos Anjos et al. 2020).

The Movement System Task Force of the Academy of Neurologic Physical Therapy proposed a framework to standardize movement analysis in individuals with neurologic disorders. This initiative introduced a set of core tasks, including sit‐to‐stand and stand‐to‐sit transfers, standing and sitting balance, gait, step up/down, and reaching, grasping, and manipulation, to facilitate systematic evaluation of motor behavior across functional contexts (Hedman et al. 2018). This framework supports clinical reasoning by enabling identification of motor control impairments, detection of compensatory strategies, and evaluation of their impact on function and activities of daily living (Carmo et al. 2012; Tomita et al. 2020; Subramanian et al. 2022). Previous studies have examined compensatory strategies across post‐stroke tasks, primarily focusing on reaching, gait, and sit‐to‐stand transfers (Subramanian et al. 2022; Faria et al. 2010; Boukadida et al. 2015; Goyal et al. 2023). However, these findings have not been systematically summarized across core task domains, limiting our understanding of how compensatory patterns vary by functional contexts.

The American Physical Therapy Association (Quinn et al. 2021) emphasizes that physical therapists, as movement specialists, should qualitatively analyze task execution to guide evidence‐based clinical decision‐making. However, clinical practice lacks a structured synthesis of how post‐stroke motor compensations are expressed across functional tasks essential to daily activities. This scoping review aimed to map compensatory motor strategies and associated movement kinematics reported during Movement System core task performance after stroke. By organizing the evidence according to task demands, the findings may support a more systematic qualitative movement assessment in physiotherapy practice.

## 2 Materials and Methods

### 2.1 Protocol and Registration

This review followed the Joanna Briggs Institute (JBI) methodological recommendations for Systematic Scoping Reviews (Peters et al. 2020) and was registered in the Open Science Framework (OSF; DOI 10.17605/OSF.IO/VM9ST).

### 2.2 Eligibility Criteria

Eligibility criteria were structured using the PCC strategy: Patient, Concept, and Context. Original and review studies were included without language or date restrictions.

Review studies were used only to identify additional eligible primary studies through reference list screening. To prevent data overlap, duplicate primary studies identified across reviews and database searches were removed during the selection process. The target population included patients with ischemic or hemorrhagic stroke in the late subacute stage (3–6 months) or chronic stage (> 6 months) (Bernhardt et al. 2017). The concept included studies analyzing kinematic compensatory strategies and movements adaptations resulting from stroke during the performance of core tasks grouped into three categories: (1) mobility—sit‐to‐stand transfer, stand‐to‐sit transfer, gait, step up/down; (2) postural control; and (3) upper‐limb tasks—reaching, grasping, and manipulation. No contextual restrictions were applied; studies from clinical and research settings were eligible.

Studies were excluded if they did not involve humans, only simulated tasks, or were protocols, book chapters, editorial letters, guidelines, websites, or reports without empirical data.

### 2.3 Information Sources and Search

The electronic search strategy was developed in collaboration with the institutional library service and was applied consistently across all databases. One reviewer conducted the electronic searches across all selected databases (Cochrane Library, LILACS, Scopus, CINAHL, Web of Science, Science Direct, Springer, MEDLINE via PubMed, EMBASE, SciELO, and OVID). Two additional reviewers independently verified the search strategy and confirmed the completeness and accuracy of the retrieved records. All references were exported to reference management software, and duplicate records were removed prior to screening. All studies published up to August 2024 were included with no language or date restrictions. The complete electronic search strategy for one database (MEDLINE via PubMed) is available in Table S1.

### 2.4 Selection of Sources of Evidence

Duplicate records were identified using Rayyan and removed prior to screening. No automated database filters were applied before screening. Two reviewers independently screened titles and abstracts according to predefined eligibility criteria. Records considered potentially relevant were retrieved for full‐text assessment. Full texts were independently evaluated to determine final eligibility. Screening decisions and reasons for exclusion were documented in Rayyan. Inter‐reviewer agreement during screening was high (Cohen's *κ* = 0.92). Disagreements were resolved by discussion or by a third reviewer.

### 2.5 Data Extraction Process

Two reviewers independently extracted data using a standardized form developed by the authors. Extracted data included study design, population characteristics (sample size, age, stroke type, lesion site, time since stroke, and impairment level), side of motor impairment, task procedures, movement analysis systems, outcomes, and related results. The terms “more affected side” and “less affected side” refer to the limbs with greater and lesser motor impairment after stroke, respectively. This terminology was adopted because the limb often described as “unaffected” may also present subtle motor deficits; therefore, “less affected” provides a more accurate description.

Data extraction focused on two primary domains: (1) compensatory strategies and (2) movement kinematics. Compensatory strategies were defined as alternative task‐oriented motor patterns involving redistribution of movement across preserved segments or substitution of impaired elements to maintain functional performance despite neurological impairments (Levin et al. 2009). Movement kinematics included spatiotemporal parameters (e.g., movement duration, gait velocity, phase timing), and joint‐level kinematic variables (e.g., ROM across trunk, pelvis, hip, knee, ankle, and upper limb) derived from 3D motion capture, inertial sensors, or 2D video analysis.

### 2.6 Data Synthesis

A descriptive synthesis was conducted to summarize and compare the evidence. The included studies were organized into three major categories according to the functional task analyzed: (1) mobility, including sit‐to‐stand and stand‐to‐sit transfers, gait, step up/down, and turning; (2) postural control, comprising sitting and standing balance tasks; and (3) upper‐limb tasks, including reaching, grasping, and manipulation. Within each category, findings were synthesized narratively to describe task characteristics, movement analysis methods, reported outcomes, and identified compensatory strategies. All compensatory strategies and movement kinematics were extracted regardless of statistical significance. The review focused on mapping these findings rather than estimating effect magnitude. Frequencies reflect the number of studies reporting a given finding and do not represent effect size, magnitude, or clinical importance.

## 3 Results

**FIGURE 1:**
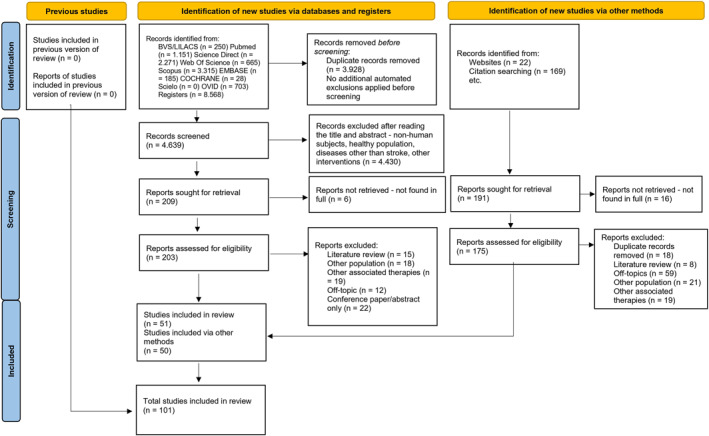
Flowchart of the study selection process.

The flowchart illustrating the study selection process (*n* = 101) is shown in Figure 1. Fourteen studies were included for the sit‐to‐stand transfer task (Mao et al. 2018; Engardt and Olsson 1992; Cheng et al. 1998; Guerriero et al. 2000; Chou et al. 2003; Richards et al. 2003; Duclos et al. 2008; Lecours et al. 2008; Galli et al. 2008; Na et al. 2016; P. F. S. Silva et al. 2017; Darwish et al. 2019; Nantawanichakorn et al. 2020; Franco et al. 2024), four for the stand‐to‐sit transfer (Engardt and Olsson 1992; Cheng et al. 1998; Na et al. 2016; Franco et al. 2024), 21 for gait (Carmo et al. 2012; Chou et al. 2003; Richards et al. 2003; Laborde et al. 2003; Titianova et al. 2003; C. M. Kim and Eng 2004; G. Chen et al. 2005; Bensoussan et al. 2006; Jonsdottir et al. 2009; Balasubramanian et al. 2010; Roerdink and Beek 2011; Hacmon et al. 2012; Polese et al. 2012; Mazuquin et al. 2014; Stanhope et al. 2014; Bonnyaud et al. 2016; H. S. Kim et al. 2016; Titus et al. 2018; Belayeva et al. 2020; Y. Wang et al. 2020; Haruyama et al. 2021), two for step up/down (Goyal et al. 2023; Novak and Brouwer 2013), eight for turning (Bonnyaud et al. 2016; Lamontagne and Fung 2009; Hollands et al. 2010; Ahmad et al. 2014; Liang et al. 2018; Abdollahi et al. 2021; Soangra et al. 2021; Abdollahi et al. 2022), 13 for postural control (Honeycutt et al. 2016; Martinez et al. 2019; Kusoffsky et al. 2001; Goldie et al. 1996; Turnbull et al. 1996; Lamontagne et al. 2003; Lin et al. 2007; Genthon et al. 2008; Mansfield et al. 2012, 2013; Pilkar et al. 2018; Chern et al. 2010; Gray et al. 2012) and 44 for the reaching, grasping, and manipulation task (Choi et al. 2023; Turnbull et al. 1996; Levin 1996; Roby‐Brami et al. 1997; Archambault et al. 1999; Cirstea and Levin 2000; Michaelsen et al. 2001; Levin et al. 2002; Cirstea et al. 2003; Roby‐Brami, Jacobs et al. 2003; Roby‐Brami, Feydy et al. 2003; Reisman and Scholz, 2003; Michaelsen et al. 2004; Zackowski et al. 2004; McCrea et al. 2005; Rose and Winstein 2005; van Vliet and Sheridan 2007; Raghavan et al. 2010; Robertson and Roby‐Brami 2011; Dejong and Lang 2012; Robertson et al. 2012; Schaefer et al. 2012; Merdler et al. 2013; Shaikh et al. 2014; Stewart et al. 2014; Levin et al. 2016; García Álvarez et al. 2017; Ma et al. 2017; Valdés et al. 2017; Tomita et al. 2018; Feingold‐Polak et al. 2021; Ota et al. 2023; Kamper et al. 2002; Wenzelburger et al. 2005; Kilbreath et al. 2006; Messier et al. 2006; Nowak et al. 2007; Michaelsen et al. 2009; Sangole and Levin 2009; Alt et al. 2011; van Kordelaar et al. 2012; Aprile et al. 2014; Thrane et al. 2019). Eight studies (Engardt and Olsson 1992; Cheng et al. 1998; Chou et al. 2003; Richards et al. 2003; Na et al. 2016; Franco et al. 2024; Bonnyaud et al. 2016; Kusoffsky et al. 2001) examined more than one task and contributed to more than one analysis.

### 3.1 Mobility

Among the 14 studies included for the sit‐to‐stand transfer task, 10 were cross‐sectional observational (Mao et al. 2018; Guerriero et al. 2000; Chou et al. 2003; Richards et al. 2003; Lecours et al. 2008; Na et al. 2016; Silva et al. 2017; Darwish et al. 2019; Nantawani et al. 2020; Franco et al. 2024), two were comparative experimental (Engardt and Olsson 1992; Galli et al. 2008), one was comparative quasi‐experimental (Duclos et al. 2008) and one was retrospective case‐control (Cheng et al. 1998). A total of 570 participants were included in these studies (343 in the stroke group, with a mean age of 58.24 ± 10.35 years). The participants' characteristics are presented in Table S2. The complete description of the task is presented in Table S3.

Among the four studies included for the stand‐to‐sit transfer task, two were cross‐sectional observational studies (Na et al. 2016; Franco et al. 2024), one was a comparative experimental study (Engardt and Olsson 1992), and one was a retrospective case‐control study (Cheng et al. 1998). A total of 191 participants were included in these studies (105 in the stroke group, with a mean age of 60.3 ± 9.56 years). The participants' characteristics are presented in Table S4. The complete description of the task is presented in Table S5.

Among the 21 studies included for the gait task, 17 were cross‐sectional observation (Carmo et al. 2012; Chou et al. 2003; Richards et al. 2003; Laborde et al. 2003; Titianova et al. 2003; Kim and Eng 2004; Chen et al. 2005; Bensoussan et al. 2006; Jonsdottir et al. 2009; Balasubramanian et al. 2010; Roerdink and Beek 2011; Hacmon et al. 2012; Stanhope et al. 2014; Bonnyaud et al. 2016; Kim et al. 2016; Titus et al. 2018; Belayeva et al. 2020; Haruyama et al., 2021), one was a case‐control (Mazuquin et al., 2014), one was an experimental comparative study (Polese et al., 2012) and one was a retrospective cohort study (Wang et al., 2020). A total of 898 participants were included in these studies (584 in the stroke group; mean age: 55.3 ± 4.3 years). The participants' characteristics are presented in Table S6. The complete description of the task is presented in Table S7.

Among the two studies analyzing the step up/down task, both were cross‐sectional observational designs (Goyal et al., 2023; Novak and Brouwer 2013). A total of 74 participants were included (47 in the stroke group; mean age: 59.51 ± 7.68 years). The participants' characteristics are detailed in Table S8, and the corresponding task protocols are described in Table S9.

For the turning task, eight cross‐sectional observational studies were identified (Bonnyaud et al. 2016; Lamontagne and Fung 2009; Hollands et al., 2010; Ahmad et al. 2014; Liang et al. 2018; Abdollahi et al. 2021; Soangra et al. 2021; Abdollahi et al. 2022). These studies included a total of 151 participants (128 in the stroke group; mean age: 61.3 ± 12.2 years). Participants' characteristics are presented in Table S10, and the task protocols are provided in Table S11.

**FIGURE 2:**
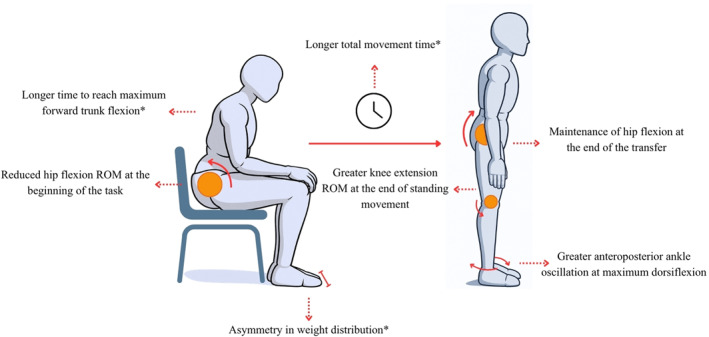
Representation of the main compensation strategies and movement kinematics in the sit‐to‐stand and stand‐to‐sit tasks. These schematic representations illustrate commonly reported compensatory strategies; not all features occur simultaneously in all individuals. All compensations refer to the more affected side when side‐specific. Strategies marked with an asterisk (*) were identified in both sit‐to‐stand and stand‐to‐sit transfers. Strategies without an asterisk were identified exclusively during sit‐to‐stand performance. ROM, range of motion. Figure created with the assistance of an AI‐based image generation tool (OpenAI, San Francisco, CA, USA).

**FIGURE 3:**
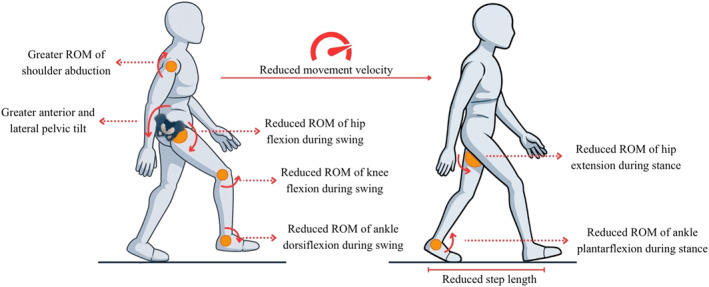
Representation of the main compensation strategies and movement kinematics in the gait task. These schematic representations illustrate commonly reported compensatory strategies; not all features occur simultaneously in all individuals. All compensations refer to the more affected side when side‐specific. ROM, range of motion. Figure created with the assistance of an AI‐based image generation tool (OpenAI, San Francisco, CA, USA).

**FIGURE 4:**
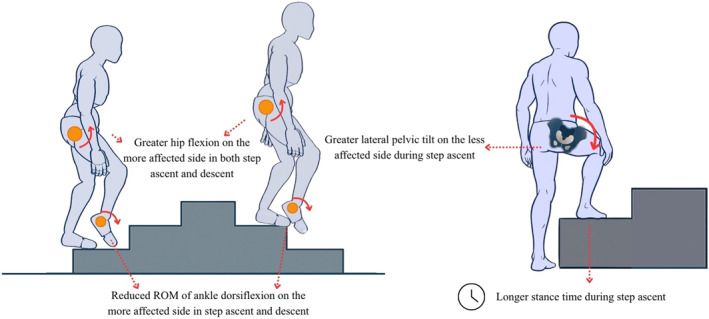
Representation of the main compensation strategies and movement kinematics in the step up/down tasks. These schematic representations illustrate commonly reported compensatory strategies; not all features occur simultaneously in all individuals. ROM, range of motion. Figure created with the assistance of an AI‐based image generation tool (OpenAI, San Francisco, CA, USA).

**FIGURE 5:**
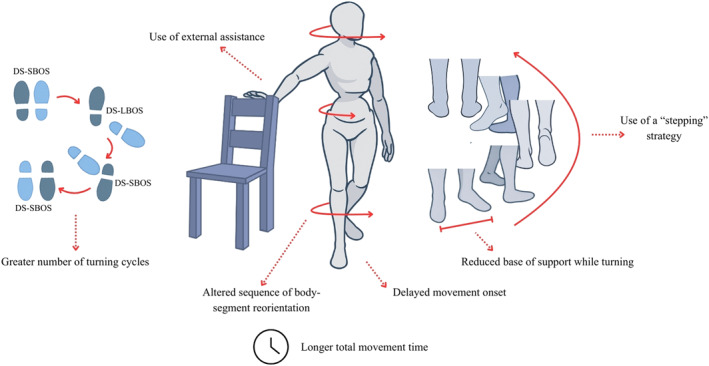
Representation of the main compensation strategies and movement kinematics in the turning task. These schematic representations illustrate commonly reported compensatory strategies; not all features occur simultaneously in all individuals. DS‐LBOS, double support—large base of support; DS‐SBOS, double support—short base of support. Figure created with the assistance of an AI‐based image generation tool (OpenAI, San Francisco, CA, USA).

A detailed summary of the main compensatory strategies and movement kinematic variables identified in mobility tasks is presented in Table 1. The table reports the frequency of each identified strategy and kinematic variable across studies involving subacute, chronic, or mixed stroke samples. Of the 54 compensatory strategies and kinematic variables identified in mobility tasks, 80% in sit‐to‐stand/stand‐to‐sit (12/15), 80% in step up/down (4/5), and 52% in gait (14/27) were identified in the sagittal plane. In turning (7 total), 57% were identified in the transverse plane. Figures 2, 3, 4, 5 provide schematic representations of these compensatory strategies and associated kinematic patterns observed during sit‐to‐stand and stand‐to‐sit transfers (Figure 2), gait (Figure 3), step up/down (Figure 4), and turning tasks (Figure 5) after stroke.

**TABLE 1:** List of the main compensation strategies and movement adaptations identified in the included studies for the mobility tasks.

Findings	Subacute (*n* = 4) *n*, (%)	Chronic, (*n* = 38) *n*, (%)	Subacute/chronic,^a^ (*n* = 7) *n*, (%)
Compensatory strategies			
Sit‐to‐stand/stand‐to‐sit task			
Greater lateral trunk displacement	0 (0)	1 (2.6)	0 (0)
Greater posterior pelvic tilt in the sitting position	0 (0)	1 (2.6)	0 (0)
Reduced anterior pelvic tilt on more affected side^b^	0 (0)	1 (2.6)	0 (0)
Maintenance of hip flexion at the end of the transfer	1 (25)	0 (0)	0 (0)
Gait task			
Greater base of support	0 (0)	3 (7.9)	0 (0)
Asymmetric foot positioning during stepping	0 (0)	1 (2.6)	0 (0)
Flat‐foot initial contact	0 (0)	1 (2.6)	0 (0)
Greater anterior pelvic tilt	1 (25)	1 (2.6)	0 (0)
Greater lateral pelvic tilt toward the more affected side	1 (25)	1 (2.6)	0 (0)
Greater lateral pelvic displacement	0 (0)	1 (2.6)	0 (0)
Greater forward trunk inclination	0 (0)	0 (0)	1 (14.2)
Greater posterior trunk rotation toward the more affected side	0 (0)	0 (0)	1 (14.2)
Reduced trunk progression during the step of the less affected limb	0 (0)	1 (2.6)	0 (0)
Step up/down			
Greater hip flexion on the more affected side in both tasks	0 (0)	1 (2.6)	0 (0)
Greater lateral pelvic tilt on the less side during step ascent	0 (0)	1 (2.6)	0 (0)
Turning task			
Greater number of turning cycles	0 (0)	3 (7.9)	1 (14.2)
Altered sequence of body‐segment reorientation during turning	0 (0)	2 (5.2)	1 (14.2)
Delayed movement onset	0 (0)	1 (2.6)	0 (0)
Reduced base of support during turning	0 (0)	1 (2.6)	0 (0)
Use of a “stepping” strategy to complete the turn	0 (0)	0 (0)	1 (14.2)
Use of external support	0 (0)	0 (0)	1 (14.2)
Movement kinematics			
Sit‐to‐stand/stand‐to‐sit task			
Longer time to reach the maximum trunk flexion	0 (0)	1 (2.6)	0 (0)
Longer time to achieve maximum extension velocity	0 (0)	1 (2.6)	0 (0)
Reduced maximum body extension velocity	0 (0)	1 (2.6)	0 (0)
Reduced ROM of hip flexion at task initiation	1 (25)	0 (0)	0 (0)
Greater ROM of knee extension at the end of the standing phase	1 (25)	0 (0)	0 (0)
Greater ankle dorsiflexion at the movement initiation	0 (0)	1 (2.6)	0 (0)
Greater anteroposterior ankle oscillation at maximum dorsiflexion	1 (25)	0 (0)	0 (0)
Greater ROM of shoulder flexion	0 (0)	1 (2.6)	0 (0)
Greater ROM of shoulder internal rotation	0 (0)	1 (2.6)	0 (0)
Greater ROM of shoulder elevation	0 (0)	1 (2.6)	0 (0)
Gait task			
Reduced gait velocity	0 (0)	4 (10.5)	0 (0)
Longer single‐support time on the less affected side	0 (0)	1 (2.6)	1 (14.2)
Longer swing time of the more affected limb	0 (0)	0 (0)	1 (14.2)
Shorter swing time of the less affected limb	0 (0)	1 (2.6)	1 (14.2)
Reduced ROM of hip flexion during swing	0 (0)	4 (10.5)	1 (14.2)
Greater ROM of hip internal rotation during stance	1 (25)	1 (2.6)	0 (0)
Greater ROM of hip internal rotation during swing	0 (0)	1 (2.6)	0 (0)
Reduced ROM of hip external rotation during stance	0 (0)	1 (2.6)	0 (0)
Greater ROM of knee flexion at initial contact	0 (0)	1 (2.6)	0 (0)
Greater ROM of knee flexion during loading response	0 (0)	1 (2.6)	0 (0)
Reduced ROM of knee extension during stance	1 (25)	2 (5.2)	0 (0)
Reduced ROM of ankle dorsiflexion during stance	0 (0)	1 (2.6)	0 (0)
Reduced ROM of ankle plantarflexion during stance	1 (25)	4 (10.5)	0 (0)
Greater ROM of pelvic rotation	0 (0)	1 (2.6)	0 (0)
Greater ROM of shoulder abduction	0 (0)	3 (12.5)	6 (33.3)
Reduced ROM of shoulder extension	0 (0)	1 (2.6)	0 (0)
Reduced ROM of shoulder external rotation	0 (0)	1 (2.6)	0 (0)
Greater ROM of elbow flexion	0 (0)	1 (2.6)	0 (0)
Step up/down			
Reduced ROM of ankle dorsiflexion on the more affected side	0 (0)	1 (2.6)	0 (0)
Turning task			
Greater ROM of pelvic obliquity	0 (0)	1 (2.6)	0 (0)

Abbreviation: ROM, range of motion.

^a^ Studies that did not differentiate between subacute and chronic stroke participants when reporting compensatory strategies.

^b^ These compensations were observed at task initiation and at the midpoint of the task.

### 3.2 Postural Control

Among the 13 studies included for the postural control task, nine were cross‐sectional observational studies (Kusoffsky et al. 2001; Goldie et al. 1996; Turnbull et al. 1996; Lamontagne et al. 2003; Lin et al. 2007; Genthon et al. 2008; Mansfield et al. 2012; Mansfield et al. 2013; Pilkar et al. 2018) and four were comparative experimental studies (Honeycutt et al. 2016; Martinez et al. 2019; Chern et al. 2010; Gray et al. 2012). A total of 561 individuals were included (387 in the stroke group; mean age of 63.2 ± 12.12). The participants' characteristics are presented in Table S12. The full task description is presented in Table S13.

**FIGURE 6:**
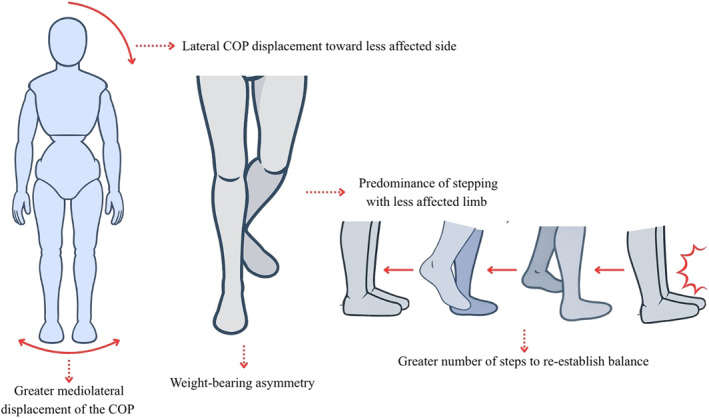
Representation of the main compensation strategies and movement kinematics in the postural control tasks. These schematic representations illustrate commonly reported compensatory strategies; not all features occur simultaneously in all individuals. COP, center of pressure. Figure created with the assistance of an AI‐based image generation tool (OpenAI, San Francisco, CA, USA).

A detailed summary of the main compensatory strategies and movement kinematic variables identified in postural control tasks is presented in Table 2. The table reports the frequency of each identified strategy and kinematic variable across studies involving subacute, chronic, or mixed stroke samples. Of the 16 compensatory strategies and kinematic variables identified in postural control tasks, 44% were identified in the sagittal plane and 44% in the frontal plane, with none in the transverse plane; 12% involved multiple planes. Figure 6 provides a schematic representation of these compensatory strategies and associated kinematic patterns observed during postural control tasks after stroke.

**TABLE 2:** List of the main compensation strategies and movement adaptations identified in the included studies for the postural control tasks.

Findings	Subacute (*n* = 4) *n*, (%)	Chronic (*n* = 7) *n*, (%)	Subacute/chronic^a^ (*n* = 2) *n*, (%)
Compensatory strategies			
Weight‐bearing asymmetry	1 (25)	3 (42.8)	1 (50)
Reduced weight‐shifting capacity in the anterior, mediolateral, and posterior directions	1 (25)	1 (14.2)	0 (0)
Predominant stepping with the less affected limb	1 (25)	1 (14.2)	0 (0)
Increased number of steps to re‐establish balance	1 (25)	1 (14.2)	0 (0)
Use of external support to maintain posture	1 (25)	0 (0)	0 (0)
Movement kinematics			
Greater mediolateral COP displacement	0 (0)	2 (28.5)	1 (50)
Reduced variability of COM acceleration	0 (0)	1 (14.2)	0 (0)
Greater anteroposterior COP displacement	0 (0)	1 (14.2)	0 (0)
Greater anterior COP displacement during reaching tasks	0 (0)	1 (14.2)	0 (0)
Lateral COP displacement toward the less affected side during quiet standing	1 (25)	1 (14.2)	0 (0)
Reduced COP displacement under static balance task	0 (0)	1 (14.2)	0 (0)
Irregular COP trajectory	0 (0)	1 (14.2)	0 (0)
Reduced whole‐body COM acceleration during rapid squats	1 (25)	0 (0)	0 (0)
Reduced head movement speed	0 (0)	0 (0)	1 (50)

Abbreviations: COM, center of mass; COP, center of pressure.

^a^ Studies that did not differentiate between subacute and chronic stroke participants when reporting compensatory strategies.

### 3.3 Upper‐Limb Tasks

Among the 44 studies on reaching, grasping, and manipulation tasks, 30 were comparative experimental studies (Levin 1996; Roby‐Brami et al. 1997; Archambault et al. 1999; Cirstea and Levin 2000; Michaelsen et al. 2001; Levin et al. 2002; Cirstea et al. 2003; Roby‐Brami, Javobs et al. 2003; Roby‐Brami, Feydy et al. 2003; Reisman and Scholz 2003; Michaelsen et al. 2004; Zackowski et al., 2004; McCrea et al., 2005; Rose and Winstein 2005; van Vliet and Sheridan 2007; Raghavan et al. 2010; Robertson et al. 2011; Dejong and Lang 2012; Robertson et al. 2012; Schaefer et al. 2012; Merdler et al. 2013; Shaikh et al. 2014; Stewart et al. 2014; Levin et al. 2016; García Álvarez et al. 2017; Ma et al. 2017; Valdés et al. 2017; Tomita et al. 2018; Feingold‐Polak et al. 2021; Ota et al. 2023) and 14 were cross‐sectional observational studies (Choi et al. 2023; Kusoffsky et al. 2001; Kamper et al., 2002; Wenzelburger et al. 2005; Kilbreath et al. 2006; Messier et al. 2006; Nowak et al. 2007; Michaelsen et al. 2009; Sangole and Levin 2009; Alt et al. 2011; van Kordelaar et al. 2012; Aprile et al. 2014; Thrane et al. 2019; Padilla‐Magaña et al. 2022). A total of 1319 participants were included in these studies (853 in the stroke group; mean age, 60 ± 13.8 years). The participants' characteristics are presented in Table S14. The full task description is presented in Table S15.

**FIGURE 7:**
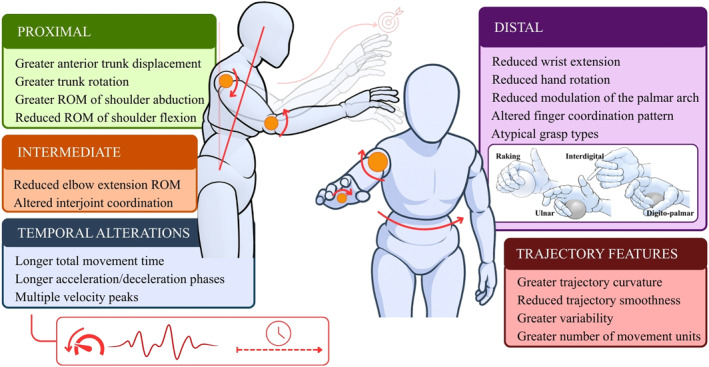
Representation of the main compensation strategies and movement kinematics during upper‐limb tasks (reaching, grasping, and manipulation). These schematic representations illustrate commonly reported compensatory strategies; not all features occur simultaneously in all individuals. All side‐specific compensations refer to the more affected limb. ROM, range of motion. Figure created with the assistance of an AI‐based image generation tool (OpenAI, San Francisco, CA, USA).

A detailed summary of the main compensatory strategies and movement kinematic variables identified in upper‐limb tasks is presented in Table 3. The table reports the frequency of each identified strategy and kinematic variable across studies involving subacute, chronic, or mixed stroke samples. Of the 89 compensatory strategies and kinematic variables identified in upper‐limb tasks, 58% were identified in the sagittal plane, 20% involved multiple planes, 14% were identified in the frontal plane, and 8% in the transverse plane. Figure 7 provides a schematic representation of these compensatory strategies and associated kinematic patterns observed during upper‐limb reaching, grasping, and manipulation tasks after stroke.

**TABLE 3:** List of the main compensation strategies and movement adaptations identified in the included studies for the upper‐limb tasks.

Findings	Subacute (*n* = 2) *n*, (%)	Chronic (*n* = 24) *n*, (%)	Subacute/chronic^a^ (*n* = 18) *n*, (%)
Compensatory strategies			
Greater anterior trunk displacement	1 (50)	8 (33.3)	6 (33.3)
Greater number of movement units	0 (0)	4 (16.6)	5 (27.7)
Greater trunk rotation	0 (0)	5 (20.8)	3 (16.6)
Greater end‐point error	0 (0)	4 (16.6)	2 (11.1)
Greater anterior trunk inclination	0 (0)	3 (12.5)	1 (5.5)
Altered interjoint coordination between the shoulder and elbow of the more affected limb	0 (0)	4 (16.6)	0 (0)
Reduced movement distance	0 (0)	4 (16.6)	0 (0)
Reduced upper‐limb displacement	0 (0)	3 (12.5)	0 (0)
Greater lateral trunk displacement	0 (0)	1 (4.2)	2 (11.1)
Greater arm‐plane angle	0 (0)	3 (12.5)	0 (0)
Reduced anterior trunk displacement	0	2 (8.3)	0 (0)
Greater hand aperture	2 (100)	0 (0)	0 (0)
Trajectory irregularity	0 (0)	2 (8.3)	0 (0)
Greater variability of movement trajectory	0 (0)	2 (8.3)	0 (0)
Movement kinematics			
Longer total movement time	1 (50)	15 (62.5)	9 (50)
Reduced ROM of elbow extension	1 (50)	14 (58.3)	10 (55.5)
Reduced peak reach velocity	1 (50)	9 (37.5)	6 (33.3)
Reduced ROM of shoulder flexion	0 (0)	5 (20.8)	3 (16.6)
Greater ROM of shoulder abduction	0 (0)	3 (12.5)	6 (33.3)
Greater trajectory curvature	1 (50)	4 (16.6)	3 (16.6)
Longer durations of the acceleration phase	1 (50)	5 (20.8)	1 (5.5)
Longer durations of the deceleration phase	0 (0)	4 (16.6)	2 (11.1)
Multiple velocity peaks during reaching	0 (0)	5 (20.8)	1 (5.5)
Reduced ROM of the shoulder horizontal adduction	0 (0)	5 (20.8)	0 (0)
Longer time to achieve maximum hand aperture	0 (0)	2 (8.3)	2 (11.1)
Longer durations of the transport phase	0 (0)	1 (4.2)	3 (16.6)
Reduced movement speed generally	0 (0)	4 (16.6)	0 (0)
Higher hand velocity	0 (0)	1 (4.2)	3 (16.6)
Greater ROM of anterior trunk flexion	0 (0)	3 (12.5)	1 (5.5)
Longer duration of the reaching phase	0 (0)	1 (4.2)	2 (11.1)
Longer duration of the pre‐transfer phase	0 (0)	2 (8.3)	1 (5.5)
Greater ROM of shoulder internal rotation	0 (0)	1 (4.2)	2 (11.1)
Greater ROM of shoulder elevation	0 (0)	0 (0)	3 (16.6)
Reduced wrist extension ROM	0 (0)	2 (8.3)	1 (5.5)
Reduced hand elevation	0 (0)	2 (8.3)	1 (5.5)
Longer duration of the pre‐shaping phase	0 (0)	2 (8.3)	0 (0)
Longer duration of the return of the object to the table phase	0 (0)	0 (0)	2 (11.1)
Reduced upper limb movement speed	0 (0)	2 (8.3)	0 (0)
Higher trunk velocity	0 (0)	2 (8.3)	0 (0)

*Note:* ≤ 1 report: Less frequently reported findings included trunk coordination alterations (e.g., trunk movement preceding hand movement, reduced trunk rotation, reduced lateral trunk displacement), trajectory and movement control changes (e.g., reduced smoothness, greater segmentation, directional variability, reduced trajectory directionality), atypical grasp patterns (e.g., raking, ulnar, interdigital, digito‐palmar), altered hand shaping and grasp modulation, and joint‐specific kinematic changes involving the shoulder, scapula, forearm, wrist, and fingers (e.g., changes in ROM of MCP, PIP, and IP joints). Additional isolated findings involved timing and velocity parameters (e.g., changes in grasp duration, transport velocity, and peak deceleration timing).

Abbreviations: IP, interphalangeal joint; MCP, metacarpophalangeal joint; PIP, proximal interphalangeal joint; ROM, range of motion.

^a^ Studies that did not differentiate between subacute and chronic stroke participants when reporting compensatory strategies.

## 4 Discussion

This review mapped the main compensatory strategies and movement kinematic variables across mobility, postural control, and upper‐limb tasks after stroke. These findings were observed in both late subacute and chronic stages and were predominantly identified in the sagittal plane. Several consistent compensations and kinematic deviations were described across functional tasks, such as asymmetry in load distribution, longer movement duration, reduced joint excursion, and increased contribution of proximal segments.

**FIGURE 8:**
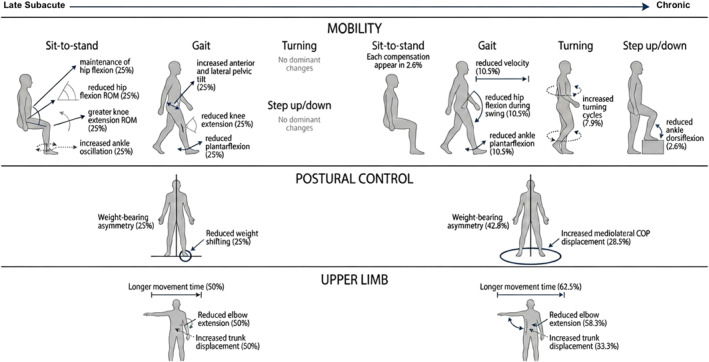
Schematic overview of compensatory strategies and movement kinematic alterations across mobility, postural control, and upper‐limb tasks in subacute and chronic stroke. These schematic representations illustrate commonly reported compensatory strategies; not all features occur simultaneously in all individuals. Percentages indicate the proportion of included studies reporting each alteration within each stage. Figure created with the assistance of Google Gemini (Google LLC, Mountain View, CA, USA).

Figure 8 summarizes the distribution of compensatory strategies and movement kinematic variables across core tasks in the late subacute and chronic stages after stroke. In mobility tasks, late subacute samples showed variable joint contributions. Chronic samples showed reduced movement velocity and lower‐limb joint excursion. In postural control, weight‐bearing asymmetry was present in both stages but was reported more frequently in the chronic stage. In upper‐limb tasks, longer movement time and greater trunk displacement were observed in both stages. Movement slowing was more frequent in chronic samples. Overall, movement slowing, asymmetry, and increased proximal segment contribution were more frequently reported in the chronic stage and may reflect stable motor solutions associated with persistent deficits (Lum et al. 2009; Roby‐Brami, Feydy et al. 2003). However, greater proximal recruitment and slower execution may also support task performance under increased environmental demands (Levin et al. 2009; Lum et al. 2009).

Although many studies assessed movement across multiple planes, compensatory strategies and associated kinematic variables were reported predominantly in the sagittal plane. Post‐stroke motor impairment reduces selective motor control and torque generation in flexor and extensor muscle groups. In the lower limb, this deficit limits plantarflexor propulsion in late stance and reduces knee and hip flexion during swing, impairing forward progression (Laborde et al. 2003; Titianova et al. 2003). In the upper limb, flexor overactivity combined with impaired elbow extension restricts forward arm transport and is frequently accompanied by trunk displacement (Levin et al. 2009; Cirstea et al. 2003). Because most functional tasks depend on anterior–posterior displacement and antigravity control, both impairment expression and compensatory reorganization are most evident in the sagittal plane after stroke.

The following discussion is structured into three domains: mobility, postural control, and upper‐limb tasks.

### 4.1 Mobility Tasks

The analysis of mobility studies revealed several recurrent compensatory strategies and movement kinematic alterations. During sit‐to‐stand and stand‐to‐sit transfers, a longer movement time was one of the reported kinematic alterations. Additional adaptations involving joint motion, segmental coordination, and task‐phase timing were also described across individual studies. In gait, reported alterations included reduced gait velocity, asymmetries in temporal parameters and reduced ROM of hips, knees, and ankles during specific gait phases. During step up/down tasks, some studies reported reduced ROM of knee flexion on the more affected side, particularly during ascent. During turning, the frequently reported alterations included a greater number of turning cycles, and altered segment reorientation.

Individuals after stroke commonly present with weakness of lower‐limb extensor and trunk muscles, in addition to inter‐joint incoordination, which impairs the execution of mobility tasks (Franco et al. 2024; Silva et al. 2015; He et al. 2023). The sit‐to‐stand movement is initiated by forward trunk inclination, followed by a body extension phase once the hips rise from the seat. At this stage, body weight support is transferred to the lower‐limb extensor muscles (Kralj et al. 1990). Conversely, during the stand‐to‐sit movement, individuals must control deceleration against gravity before the hips reach the seat (Kralj et al. 1990). Difficulties in performing these sequential phases lead to longer total movement times.

Reduced gait speed has been consistently associated with reduced functional capacity and increased risk of falls (Fulk et al. 2017; Bower et al. 2019; Patel et al. 2025). These changes may be associated with advanced age, impaired balance, and knee extensor weakness (Jasper et al. 2025). These limitations further restrict participation in outdoor activities and community engagement (Olawale et al. 2018; Fulk et al. 2025). During turning, many individuals exhibit balance deficits due to muscular incoordination (Lewallen et al. 2021). Consequently, they shorten swing time of the less affected limb, increase double‐support duration, and increase the number of turning cycles and total movement time (Abdollahi et al. 2022).

Weakness of the more affected limb may contribute to a shift in the center of mass toward the less affected side. This strategy facilitates sit‐to‐stand and stand‐to‐sit transfers by enhancing control over the less affected limb (Lecours et al., 2008; Chen et al. 2010; Oh et al. 2010). This biomechanical adjustment may be associated with asymmetrical weight‐bearing distribution. In both gait and turning, this asymmetry is reflected by increased double‐support duration and longer single‐limb support on the less affected side, which may represent a compensatory response to instability and reduced confidence in the more affected limb (Schinkel‐Ivy et al. 2017; Chang et al. 2021).

Reduced hip, knee, and ankle ROM may be associated with muscle weakness and joint stiffness (Li 2023). Specifically, knee stiffness may limit flexion during swing (Li 2023), secondary to weakness of muscles such as the biceps femoris and gastrocnemius (Wang et al., 2017) and hyperactivation of the rectus femoris (Akbas et al. 2020). This restriction may lead to compensatory strategies such as pelvic hiking during stepping (Goyal et al. 2023). Another contributing factor may be altered muscle activation timing, which can limit effective force generation and increase antagonist co‐contraction during gait (Ghédira et al. 2021). Similar kinematic alterations are even more evident during step up/down tasks due to the greater mechanical demands placed on the hip, knee, and ankle joints (Protopapadaki et al. 2007).

### 4.2 Postural Control Task

Analysis of postural control studies revealed that the most frequently reported findings were asymmetrical weight‐bearing distribution and increased mediolateral displacement of the center of pressure (COP), while reduced weight‐shifting capacity in the anterior, mediolateral, and posterior directions was reported in fewer studies.

Individuals after stroke frequently exhibit weight‐bearing asymmetry favoring the less affected side (Mansfield et al. 2013). This typically occurs due to a shift of the COP toward that side to compensate for deficits in the more affected limb, enhance stability, or result from sensory alterations in the more affected limb (Chu et al. 2015; Tasseel‐Ponche et al. 2015). In some cases, the more affected side may be favored, associated with learned strategies during rehabilitation (Mansfield et al. 2013) or with an anticipatory mechanism to prepare for balance recovery steps with the less affected limb (Tasseel‐Ponche et al. 2015).

Increased mediolateral COP displacement may reflect greater postural instability, requiring larger adjustments to maintain balance (Roerdink et al. 2006). Reduced ability to shift weight in anterior, mediolateral, and posterior directions is associated with reliance on increased cognitive control and reduced adaptability of the postural system, demanding more attentional resources and voluntary control (Roerdink et al. 2006).

### 4.3 Upper‐Limbs Tasks

The analysis of studies addressing upper‐limb tasks revealed that the predominant findings were longer total movement time, reduced elbow extension ROM, lower peak reaching velocity, greater trunk displacement and rotation. Some studies also reported increased shoulder abduction accompanied by reduced horizontal adduction and flexion, a greater number of movement units, longer acceleration and deceleration phases, and greater end‐point error.

Muscle overactivity associated with impaired motor control, combined with weakness of key muscles such as the shoulder flexors and elbow extensors, directly affects the quality of reaching movements (McCluskey et al. 2024). Reduced force generation and impaired selective motor control limit effective forward transport of the upper limb (McCluskey et al. 2024). When the available motor capacity is insufficient to meet task demands, individuals reorganize movement to preserve goal‐directed performance (McCluskey et al. 2024). To compensate for distal weakness and impaired torque production at the shoulder and elbow, individuals rely on forward trunk displacement and rotation, particularly during reaching compared with grasping and manipulation tasks (Mandon et al. 2016; Jayasinghe et al. 2021). Trunk movement therefore represents a strategic redistribution of movement to preserved proximal segments rather than merely a biomechanical consequence of impairment (Ota et al. 2023). Greater shoulder abduction often accompanies reduced horizontal adduction, reflecting altered interjoint coordination during task execution (Choi et al. 2023; Roby‐Brami and Feydy 2003; Ota et al. 2023).

Motor control impairments and compensatory reliance are associated with reduced upper‐limb movement smoothness, necessitating multiple trajectory corrections and consequently increasing the number of movement units (Alt et al. 2011). Longer acceleration and deceleration phases are also observed and may represent an attempt to enhance accuracy; nevertheless, end‐point errors persist despite these strategies (Merdler et al. 2013; Stewart et al. 2014; Feingold‐Polak et al. 2021). Overall, these kinematic alterations contribute to longer total movement time (Stewart et al. 2014; Feingold‐Polak et al. 2021; Thrane et al. 2019).

### 4.4 Future Implications, Limitations, and Strengths

This review map reported compensatory strategies and does not establish normative movement patterns or optimal clinical strategies. Several limitations should be acknowledged. Methodological quality was not assessed, and findings were not weighted according to study quality or effect size. Publication bias cannot be excluded. Motor impairment was not stratified by stroke severity, lesion location, or impairment level, which may have influenced the distribution of compensatory strategies and kinematic variables across tasks. Additionally, there was substantial heterogeneity across study designs and motion analysis methods, which may have influenced the variability in reported findings. Despite these limitations, the study has important strengths. A comprehensive search of major databases identified a substantial number of studies. The task‐based organization provided a structured synthesis of core functional tasks. Inclusion of both late subacute and chronic stages allowed examination of compensatory strategies across recovery stages.

In conclusion, compensatory strategies and associated movement kinematic variables were identified across mobility, postural control, and upper‐limb tasks after stroke. Mobility tasks were commonly characterized by longer transfer time, reduced gait velocity and increased turning cycles, whereas other joint‐specific adaptations were reported in fewer studies. Weight‐bearing asymmetry was the most frequent finding in postural control tasks. Upper‐limb tasks showed longer movement time, reduced elbow extension, and greater trunk displacement. Characterization of these compensatory strategies and their kinematic features guide systematic qualitative movement assessment in clinical practice. Future research should develop standardized, low‐cost instruments to support structured observation of compensatory strategies and task‐specific intervention planning after stroke.

### 4.5 Implications of Physiotherapy Practice

- Compensatory strategies are frequently observed during mobility, postural control, and upper‐limb tasks after stroke and should be systematically considered during functional movement assessment.
- Observation of task‐specific movement kinematics can support structured qualitative analysis of motor performance during functional tasks.
- Task‐based analysis of compensatory strategies may inform clinical reasoning and guide task‐specific physiotherapy interventions after stroke.

## Author Contributions

**Pedro Henrique Sousa de Andrade:** methodology, investigation, data curation, formal analysis, writing – original draft, writing – review and editing. **Caroline Rodrigues Osawa:** methodology, investigation, data curation, writing – review and editing. **Maria Eduarda Salum Aveiro:** methodology, investigation, data curation, writing – review and editing. **Clara Rezende Rocha:** methodology, investigation, data curation, writing – review and editing. **Rodrigo Bazan:** writing – review and editing. **Luciane Aparecida Pascucci de Sande Souza:** conceptualization, writing – review and editing, supervision. **Gustavo José Luvizutto:** conceptualization, formal analysis, writing – original draft, writing – review and editing, supervision, funding acquisition.

## Funding

This work was supported by the Fundação de Amparo à Pesquisa do Estado de Minas Gerais (FAPEMIG), Brazil (Grant 14504).

## Ethics Statement

The authors have nothing to report.

## Consent

The authors have nothing to report.

## Conflicts of Interest

The authors declare no conflicts of interest.

## Supporting information


**Table S1:** Search strategy for each database.

**Table S2:** Description of the participants' characteristics of each study included for the sit‐to‐stand transfer task.

**Table S3:** Description of the sit‐to‐stand transfer task in each included study.

**Table S4:** Description of the participants' characteristics of each study included for the stand‐to‐sit transfer task.

**Table S5:** Description of the stand‐to‐sit transfer task in each included study.

**Table S6:** Description of the participants' characteristics in each included study for the gait task.

**Table S7:** Description of the gait task in each included study.

**Table S8:** Description of the participants' characteristics in each included study for the climb up and down stairs task.

**Table S9:** Task description for the stair ascent and descent task in each included study.

**Table S10:** Description of the participants' characteristics in each included study for the turning task.

**Table S11:** Task description for the turning task in each included study.

**Table S12:** Description of the participants' characteristics in each included study for the postural control task.

**Table S13:** Description of the postural control task in each included study.

**Table S14:** Description of the participants' characteristics in each included study for the reaching, grasping and manipulation task.

**Table S15:** Description of the reaching, grasping and manipulation tasks in each included study.

## Data Availability

This article is a scoping review; no new data were created. All data were extracted from previously published studies cited in this manuscript. The data‐charting matrix can be made available as Supplementary Material upon request.
