# Supplementary material for: Task‐Based Mapping of Compensatory Strategies and Movement Kinematics After Stroke: A Systematic Scoping Review
Source: Physiother Res Int. 2026 Apr 13;31(2):e70215. doi: 10.1002/pri.70215 (PMC13076240; doi:10.1002/pri.70215)
Supplement: Supplementary file 1 — Table S1: Search strategy for each database. [file PRI-31-e70215-s005.docx]

Table S1. Search strategy for each database.

PUBMED:

| #1 | "Stroke"[Mesh] OR Stroke OR Strokes OR (Cerebrovascular Accident) OR (Cerebrovascular Accidents) OR (CVA (Cerebrovascular Accident)) OR (CVAs (Cerebrovascular Accident)) OR (Cerebrovascular Apoplexy) OR (Apoplexy, Cerebrovascular) OR (Vascular Accident, Brain) OR (Brain Vascular Accident) OR (Brain Vascular Accidents) OR (Vascular Accidents, Brain) OR (Cerebrovascular Stroke) OR (Cerebrovascular Strokes) OR (Stroke, Cerebrovascular) OR (Strokes, Cerebrovascular) OR Apoplexy OR (Cerebral Stroke) OR (Cerebral Strokes) OR (Stroke, Cerebral) OR (Strokes, Cerebral) OR (Stroke, Acute) OR (Acute Stroke) OR (Acute Strokes) OR (Strokes, Acute) OR (Cerebrovascular Accident, Acute) OR (Acute Cerebrovascular Accident) OR (Acute Cerebrovascular Accidents) OR (Cerebrovascular Accidents, Acute) OR "Hemorrhagic Stroke"[Mesh] OR (Hemorrhagic Stroke) OR (Hemorrhagic Strokes) OR (Stroke, Hemorrhagic) OR (Subarachnoid Hemorrhagic Stroke) OR (Hemorrhagic Stroke, Subarachnoid) OR (Stroke, Subarachnoid Hemorrhagic) OR (Subarachnoid Hemorrhagic Strokes) OR (Intracerebral Hemorrhagic Stroke) OR (Hemorrhagic Stroke, Intracerebral) OR (Intracerebral Hemorrhagic Strokes) OR (Stroke, Intracerebral Hemorrhagic) OR (Intracerebral Hemorrhage Stroke) OR (Hemorrhage Stroke, Intracerebral) OR (Intracerebral Hemorrhage Strokes) OR (Stroke, Intracerebral Hemorrhage) OR "Ischemic Stroke"[Mesh] OR (Ischemic Stroke) OR (Ischemic Strokes) OR (Stroke, Ischemic) OR (Ischaemic Stroke) OR (Ischaemic Strokes) OR (Stroke, Ischaemic) OR (Cryptogenic Ischemic Stroke) OR (Cryptogenic Ischemic Strokes) OR (Ischemic Stroke, Cryptogenic) OR (Stroke, Cryptogenic Ischemic) OR (Cryptogenic Stroke) OR (Cryptogenic Strokes) OR (Stroke, Cryptogenic) OR (Cryptogenic Embolism Stroke) OR (Cryptogenic Embolism Strokes) OR (Embolism Stroke, Cryptogenic) OR (Stroke, Cryptogenic Embolism) OR (Wake-up Stroke) OR (Stroke, Wake-up) OR (Wake up Stroke) OR (Wake-up Strokes) OR (Acute Ischemic Stroke) OR (Acute Ischemic Strokes) OR (Ischemic Stroke, Acute) OR (Stroke, Acute Ischemic) OR (Stroke, Lacunar) OR "Stroke, Lacunar"[Mesh] OR (Lacunar Stroke) OR (Lacunar Strokes) OR (Strokes, Lacunar) OR (Lacunar Syndrome) OR (Lacunar Syndromes) OR (Syndrome, Lacunar) OR (Syndromes, Lacunar) OR (Infarction, Lacunar) OR (Infarctions, Lacunar) OR (Lacunar Infarction) OR (Lacunar Infarctions) OR (Infarct, Lacunar) OR (Infarcts, Lacunar) OR (Lacunar Infarct) OR (Lacunar Infarcts) OR "Brain Infarction"[Mesh] OR (Brain Infarction) OR (Brain Infarctions) OR (Brain Infarct) OR (Brain Infarcts) OR (Anterior Circulation Brain Infarction) OR (Brain Venous Infarction) OR (Brain Venous Infarctions) OR (Venous Brain Infarction) OR (Venous Brain Infarctions) OR (Anterior Cerebral Circulation Infarction) OR (Posterior Circulation Brain Infarction) |
| --- | --- |
| #2 AND | (Adaptive strategy) OR (Adaptive Strategies) OR (Compensatory Strategy) OR (Compensatory Strategies) OR (Behavioral Compensation) OR (Behavioral Compensations) OR (Behavioral Adaptation) OR (Behavioral Adaptations) |
| #3 AND | "Gait"[Mesh] OR Gait OR Gaits |
| #4 OR | "Sitting Position"[Mesh] OR (Sitting Position) OR (Position, Sitting) OR (Sitting Positions) OR (Sitting) OR (Seated Position) OR (Position, Seated) OR (Positions, Seated) OR (Seated Positions) |
|  | (stand-to-sit) |
| #5 OR | "Standing Position"[Mesh] OR (Standing Position) OR (Position, Standing) OR (Standing Positions) |
|  | (sit-to-stand) |
| #6 OR | "Stair Climbing"[Mesh] OR (Stair climbing) OR (Climbing, Stair) OR (Stair Navigation) OR (Navigation, Stair) |
|  | (step up) OR (step down) OR (step up a stair) OR (step up stairs) OR (Walk up a stair) OR (Walk up stairs) OR (step down a stair) OR (step down stairs) OR (Walk down a stair) OR (Walk down stairs) OR (walking up a stair) OR (walking up stairs) OR (walking down a stair) OR (walking down stairs) |
| #7 OR | Turn OR turning OR (180 turn) OR pivot OR pivoting |
| #8 OR | "Postural Balance"[Mesh] OR (Postural Balance) OR (Posture Equilibrium) OR (Equilibrium, Posture) OR (Posture Equilibriums) OR (Balance, Postural) OR (Postural Equilibrium) OR (Equilibrium, Postural) OR (Posture Balance) OR (Balance, Posture) OR (Posture Balances) OR (Musculoskeletal Equilibrium) OR (Equilibrium, Musculoskeletal) OR (Postural Control) OR (Control, Postural) OR (Postural Controls) OR (Posture Control) OR (Control, Posture) OR (Posture Controls) |
| #9 OR | "Hand Strength"[Mesh] OR (Hand Strength) OR (Strength, Hand) OR (Grip Strength) OR (Strength, Grip) OR (Hand Grip Strength) OR (Grip Strength, Hand) OR (Strength, Hand Grip) OR (Grip) OR (Grips) OR (Grasp) OR (Grasps) |
|  | (Reach) OR (Reaching) OR (Hand Function) OR (Manipulation) |

BVS/LILACS:

|  | mh:"AVC Trombótico" OR (AVC Trombótico) OR (AVC Trombótico Agudo) OR (Thrombotic Stroke) OR (Acute Thrombotic Stroke) OR (Acute Thrombotic Strokes) OR (Stroke, Acute Thrombotic) OR (Stroke, Thrombotic) OR (Thrombotic Stroke, Acute) OR (Thrombotic Strokes) OR (Accidente Cerebrovascular Trombótico) OR (Accidente Cerebrovascular Trombótico Agudo) OR mh:"AVC Isquêmico" OR (AVC Isquêmico) OR (Acidente Vascular Cerebral ao Despertar) OR (Acidente Vascular Cerebral Isquêmico) OR (AVC ao Acordar) OR (AVC Criptogênico) OR (AVC de Embolia Criptogênica) OR (AVC isquêmico Agudo) OR (AVC Isquêmico Criptogênico) OR (AVC Tipo WUS) OR (Icto ao Despertar) OR (Icto Isquêmico) OR (Ischemic Stroke) OR (Acute Ischemic Stroke) OR (Acute Ischemic Strokes) OR (Cryptogenic Embolism Stroke) OR (Cryptogenic Embolism Strokes) OR (Cryptogenic Ischemic Stroke) OR (Cryptogenic Ischemic Strokes) OR (Cryptogenic Stroke) OR (Cryptogenic Strokes) OR (Embolism Stroke, Cryptogenic) OR (Ischaemic Stroke) OR (Ischaemic Strokes) OR (Ischemic Stroke, Acute) OR (Ischemic Stroke, Cryptogenic) OR (Ischemic Strokes) OR (Stroke, Acute Ischemic) OR (Stroke, Cryptogenic) OR (Stroke, Cryptogenic Embolism) OR (Stroke, Cryptogenic Ischemic) OR (Stroke, Ischaemic) OR (Stroke, Ischemic) OR (Stroke, Wake-up) OR (Wake up Stroke) OR (Wake-up Stroke) OR (Wake-up Strokes) OR (Accidente Cerebrovascular Isquémico) OR (Accidente Cerebrovascular al Despertar) OR (Accidente Cerebrovascular Criptogénico) OR (Accidente Cerebrovascular de Embolia Criptogénica) OR (Accidente Cerebrovascular Isquémico Agudo) OR (Accidente Cerebrovascular Isquémico Criptogénico) OR mh:"Acidente Vascular Cerebral Hemorrágico" OR (Acidente Vascular Cerebral Hemorrágico) OR (Acidente Vascular Cerebral Hemorrágico Subaracnóideo) OR (AVC com Hemorragia Intracerebral) OR (AVC Hemorrágico) OR (AVC Hemorrágico Intracerebral) OR (AVC Hemorrágico Subaracnoídeo) OR (AVC Hemorrágico Subaracnóideo) OR (Derrame Cerebral) OR (Derrames Cerebrais) OR (Hemorrhagic Stroke) OR (Hemorrhage Stroke, Intracerebral) OR (Hemorrhagic Stroke, Intracerebral) OR (Hemorrhagic Stroke, Subarachnoid) OR (Hemorrhagic Strokes) OR (Intracerebral Hemorrhage Stroke) OR (Intracerebral Hemorrhage Strokes) OR (Intracerebral Hemorrhagic Stroke) OR (Intracerebral Hemorrhagic Strokes) OR (Stroke, Hemorrhagic) OR (Stroke, Intracerebral Hemorrhage) OR (Stroke, Intracerebral Hemorrhagic) OR (Stroke, Subarachnoid Hemorrhagic) OR (Subarachnoid Hemorrhagic Stroke) OR (Subarachnoid Hemorrhagic Strokes) OR (Accidente Cerebrovascular Hemorrágico) OR (Accidente Cerebrovascular Hemorrágico Intracerebral) OR (Accidente Cerebrovascular por Hemorragia Intracerebral) OR (Accidente Cerebrovascular Subaracnoideo Hemorrágico) OR (Derrame Cerebral) OR mh:"Acidente Vascular Cerebral" OR (Acidente Vascular Cerebral) OR (Acidente Cerebral Vascular) OR (Acidente Cerebrovascular) OR (Acidente Vascular Cerebral (AVC)) OR (Acidente Vascular Cerebral Agudo) OR (Acidente Vascular do Cérebro) OR (Acidente Vascular Encefálico) OR (Acidentes Cerebrais Vasculares) OR (Acidentes Cerebrovasculares) OR (Acidentes Vasculares Cerebrais) OR (Apoplexia) OR (Apoplexia Cerebral) OR (Apoplexia Cerebrovascular) OR (AVC) OR (AVC Agudo) OR (AVE) OR (Icto Cerebral) OR (Ictus Cerebral) OR (Stroke) OR (Acute Cerebrovascular Accident) OR (Acute Cerebrovascular Accidents) OR (Acute Stroke) OR (Acute Strokes) OR (Apoplexy) OR (Apoplexy, Cerebrovascular) OR (Brain Vascular Accident) OR (Brain Vascular Accidents) OR (Cerebral Stroke) OR (Cerebral Strokes) OR (Cerebrovascular Accident) OR (Cerebrovascular Accident, Acute) OR (Cerebrovascular Accidents) OR (Cerebrovascular Accidents, Acute) OR (Cerebrovascular Apoplexy) OR (Cerebrovascular Stroke) OR (Cerebrovascular Strokes) OR (CVA (Cerebrovascular Accident)) OR (CVAs (Cerebrovascular Accident)) OR (Stroke, Acute) OR (Stroke, Cerebral) OR (Stroke, Cerebrovascular) OR (Strokes) OR (Strokes, Acute) OR (Strokes, Cerebral) OR (Strokes, Cerebrovascular) OR (Vascular Accident, Brain) OR (Vascular Accidents, Brain) OR (Accidente Cerebrovascular) OR (Accidente Cerebral Vascular) OR (Accidente Cerebrovascular Agudo) OR (Accidente Vascular Cerebral) OR (Accidente Vascular del Cerebro) OR (Accidente Vascular Encefálico) OR (Accidentes Cerebrovasculares) OR (ACV Agudo) OR (Apoplejía) OR (Apoplejía Cerebral) OR (Apoplejía Cerebrovascular) OR (Ataque) OR (Ataque Cerebral) OR (Ataque Cerebrovascular) OR (Ataque Cerebrovascular Agudo) OR (Ictus) OR (Ictus Cerebral) OR mh:C10.228.140.300.775.400.750$ OR mh:C14.907.253.855.400.750$ OR mh:C10.228.140.300.775.400$ OR  mh:C14.907.253.855.400$ OR mh:C10.228.140.300.775.300$  OR mh:C14.907.253.855.300$ OR mh:C10.228.140.300.775$  OR mh:C14.907.253.855$ |
| --- | --- |
| AND | (Estratégias adaptativas) OR (Adaptive strategies) OR (Estrategias adaptativas) OR (Estratégia Compensatória) OR (Compensatory Strategy) OR (Estrategia compensatória) OR (Compensação Comportamental) OR (Behavioral Compensation) OR (Compensación conductual) OR (Adaptação Comportamental) OR (Behavioral Adaptation) OR (Adaptación conductual) |
| AND | mh:"Marcha" OR Marcha OR Gait OR Gaits OR mh:E01.370.600.250$ OR mh:G11.427.410.568.900.750$ |
| OR | mh:"Postura Sentada" OR (Postura Sentada) OR (Sitting Position) OR (Sitting Positions) OR (Sedestación) OR (Posição ao Sentar) OR (Sentar-se) OR (Stand-to-sit) OR (Sentar) OR mh:G11.427.695.575$ |
| OR | mh:"Posição Ortostática" OR (Posição Ortostática) OR (Posição em pé) OR (Standing Position) OR (Position, Standing) OR (Standing Positions) OR (Posición de Pie) OR (Bipedestación) OR (Bipedestaciones) OR (Estación Bípeda) OR (Posición Bípeda) OR (Posición Ortostática) OR (Sit-to-stand) OR (Levantar-se) OR (Levantar) OR mh:G11.427.695.600$ |
| OR | mh:"Subida de escada" OR (Subida de escada) OR (stair climbing) OR (subida de escaleras) OR mh:G11.427.410.568.900.875$ OR (Subir degraus) OR (Walk up stairs) OR (Subir escaleras) OR (Descer degraus) OR (Walk down stairs) OR (Bajar escaleras) OR mh:G11.427.410.698.277.937.500$ OR mh:I03.350.937.500$ |
| OR | (Pivoting) OR (Turn) OR (Turning) |
| OR | mh:"Equilíbrio Postural" OR (Equilíbrio Postural) OR (Controle Postural) OR  (Controle da Postura) OR (Controle de Postura) OR (Equilíbrio da Postura) OR  (Equilíbrio na Postura) OR (Postural Balance) OR (Balance, Postural) OR  (Balance, Posture) OR (Control, Postural) OR (Control, Posture) OR  (Equilibrium, Musculoskeletal) OR (Equilibrium, Postural) OR (Equilibrium, Posture) OR (Musculoskeletal Equilibrium) OR (Postural Control) OR (Postural Controls) OR (Postural Equilibrium) OR (Posture Balance) OR (Posture Balances) OR (Posture Control) OR (Posture Controls) OR (Posture Equilibrium) OR (Posture Equilibriums) OR (Equilibrio Postural) OR (Balance Postural) OR (Control Postural) OR mh:F02.830.816.541.752$ OR  mh:G07.888.750.500$ OR mh:G11.427.690$ OR mh:G11.561.790.541.595$ |
| OR | mh:"Força da Mão" OR (Força da Mão) OR (Aperto de Mão) OR (Empunhadura) OR (Força de Preensão) OR (Força de Preensão da Mão) OR (Hand Strength) OR (Grasp) OR (Grasps) OR (Grip) OR (Grip Strength) OR (Grips) OR (Hand Grip Strength) OR (Hand Strengths) OR (Fuerza de la Mano) OR (Apretón) OR (Asimiento) OR mh:E01.370.600.425.500$ OR mh:G11.427.560.500$ OR mh:SP6.270.233.111.534$ |

SCIELO:

|  | “Acidente Vascular Cerebral” OR “Acidente Cerebral Vascular” OR “Acidente Cerebrovascular” OR “Acidente Vascular Cerebral “AVC”“ OR “Acidente Vascular Cerebral Agudo” OR “Acidente Vascular do Cérebro” OR “Acidente Vascular Encefálico” OR “Acidentes Cerebrais Vasculares” OR “Acidentes Cerebrovasculares” OR “Acidentes Vasculares Cerebrais” OR “Apoplexia” OR “Apoplexia Cerebral” OR “Apoplexia Cerebrovascular” OR “AVC” OR “AVC Agudo” OR “AVE” OR “Icto Cerebral” OR “Ictus Cerebral” OR “Stroke” OR “Acute Cerebrovascular Accident” OR “Acute Cerebrovascular Accidents” OR “Acute Stroke” OR “Acute Strokes” OR “Apoplexy” OR “Apoplexy, Cerebrovascular” OR “Brain Vascular Accident” OR “Brain Vascular Accidents” OR “Cerebral Stroke” OR “Cerebral Strokes” OR “Cerebrovascular Accident” OR “Cerebrovascular Accident, Acute” OR “Cerebrovascular Accidents” OR “Cerebrovascular Accidents, Acute” OR “Cerebrovascular Apoplexy” OR “Cerebrovascular Stroke” OR “Cerebrovascular Strokes” OR “CVA “Cerebrovascular Accident”“ OR “CVAs “Cerebrovascular Accident”“ OR “Stroke, Acute” OR “Stroke, Cerebral” OR “Stroke, Cerebrovascular” OR “Strokes” OR “Strokes, Acute” OR “Strokes, Cerebral” OR “Strokes, Cerebrovascular” OR “Vascular Accident, Brain” OR “Vascular Accidents, Brain” OR “Accidente Cerebrovascular” OR “Accidente Cerebral Vascular” OR “Accidente Cerebrovascular Agudo” OR “Accidente Vascular Cerebral” OR “Accidente Vascular del Cerebro” OR “Accidente Vascular Encefálico” OR “Accidentes Cerebrovasculares” OR “ACV Agudo” OR “Apoplejía” OR “Apoplejía Cerebral” OR “Apoplejía Cerebrovascular” OR “Ataque” OR “Ataque Cerebral” OR “Ataque Cerebrovascular” OR “Ataque Cerebrovascular Agudo” OR “Ictus” OR “Ictus Cerebral” |
| --- | --- |
|  | “Estratégias adaptativas” OR “Adaptive strategies” OR “Estrategias adaptativas” OR “Estratégia Compensatória” OR “Compensatory Strategy” OR “Estrategia compensatória” OR “Compensação Comportamental” OR “Behavioral Compensation” OR “Compensación conductual” OR “Adaptação Comportamental” OR “Behavioral Adaptation” OR “Adaptación conductual” |
|  | Marcha OR Gait OR Gaits |
|  | “Postura Sentada” OR “Sitting Position” OR “Sitting Positions” OR “Sedestación” OR “Posição ao Sentar” OR “Sentar-se” OR “Stand-to-sit” OR “Sentar” |
|  | “Posição Ortostática” OR “Posição em pé” OR “Standing Position” OR “Position, Standing” OR “Standing Positions” OR “Posición de Pie” OR “Bipedestación” OR “Bipedestaciones” OR “Estación Bípeda” OR “Posición Bípeda” OR “Posición Ortostática” OR “Sit-to-stand” OR “Levantar-se” OR “Levantar” |
|  | “Subida de escada” OR “stair climbing” OR “subida de escaleras” OR “Subir degraus” OR “Walk up stairs” OR “Subir escaleras” OR “Descer degraus” OR “Walk down stairs” OR “Bajar escaleras” |
|  | “Pivoting” OR “Turn” OR “Turning” |
|  | “Equilíbrio Postural” OR “Controle Postural” OR “Controle da Postura” OR “Controle de Postura” OR “Equilíbrio da Postura” OR  “Equilíbrio na Postura” OR “Postural Balance” OR “Balance, Postural” OR  “Balance, Posture” OR “Control, Postural” OR “Control, Posture” OR  “Equilibrium, Musculoskeletal” OR “Equilibrium, Postural” OR “Equilibrium, Posture” OR “Musculoskeletal Equilibrium” OR “Postural Control” OR “Postural Controls” OR “Postural Equilibrium” OR “Posture Balance” OR “Posture Balances” OR “Posture Control” OR “Posture Controls” OR “Posture Equilibrium” OR “Posture Equilibriums” OR “Equilibrio Postural” OR “Balance Postural” OR “Control Postural” |
|  | “Força da Mão” OR “Aperto de Mão” OR “Empunhadura” OR “Força de Preensão” OR “Força de Preensão da Mão” OR “Hand Strength” OR “Grasp” OR “Grasps” OR “Grip” OR “Grip Strength” OR “Grips” OR “Hand Grip Strength” OR “Hand Strengths” OR “Fuerza de la Mano” OR “Apretón” OR “Asimiento” |

WEB OF SCIENCE:

| #1 | Stroke OR Strokes OR (Cerebrovascular Accident) OR (Cerebrovascular Accidents) OR (CVA (Cerebrovascular Accident)) OR (CVAs (Cerebrovascular Accident)) OR (Cerebrovascular Apoplexy) OR (Brain Vascular Accident) OR (Brain Vascular Accidents) OR (Cerebrovascular Stroke) OR (Cerebrovascular Strokes) OR Apoplexy OR (Cerebral Stroke) OR (Cerebral Strokes) OR (Acute Stroke) OR (Acute Strokes) OR (Acute Cerebrovascular Accident) OR (Acute Cerebrovascular Accidents) OR (Hemorrhagic Stroke) OR (Ischemic Stroke) OR (Lacunar Stroke) OR (Brain Infarction) |
| --- | --- |
| AND #2 | (Adaptive strategy) OR (Adaptive Strategies) OR (Compensatory Strategy) OR (Compensatory Strategies) OR (Behavioral Compensation) OR (Behavioral Compensations) OR (Behavioral Adaptation) OR (Behavioral Adaptations) |
| AND #3 | Gait OR Gaits |
| OR #4 | (Sitting Position) OR (Position, Sitting) OR (Sitting Positions) OR (Sitting) OR (Seated Position) OR (Position, Seated) OR (Positions, Seated) OR (Seated Positions) |
|  | (stand-to-sit) |
| OR #5 | (Standing Position) OR (Position, Standing) OR (Standing Positions) |
|  | (sit-to-stand) |
| OR #6 | (Stair climbing) OR (Stair Navigation) |
|  | (step up) OR (step down) OR (step up stairs) OR (Walk up stairs) OR (step down stairs) OR (Walk down stairs) OR (walking up stairs) OR (walking down stairs) |
| OR #7 | Turn OR turning OR (180 turn) OR pivot OR pivoting |
| OR #8 | (Postural Balance) OR (Posture Equilibrium) OR (Equilibrium, Posture) OR (Posture Equilibriums) OR (Balance, Postural) OR (Postural Equilibrium) OR (Equilibrium, Postural) OR (Posture Balance) OR (Balance, Posture) OR (Posture Balances) OR (Musculoskeletal Equilibrium) OR (Equilibrium, Musculoskeletal) OR (Postural Control) OR (Control, Postural) OR (Postural Controls) OR (Posture Control) OR (Control, Posture) OR (Posture Controls) |
| OR #9 | (Hand Strength) OR (Strength, Hand) OR (Grip Strength) OR (Strength, Grip) OR (Hand Grip Strength) OR (Grip Strength, Hand) OR (Strength, Hand Grip) OR (Grip) OR (Grips) OR (Grasp) OR (Grasps) |
|  | (Reach) OR (Reaching) OR (Hand Function) OR (Manipulation) |

EMBASE:

| #1 | 'cerebrovascular accident'/syn OR 'acute ischemic stroke'/syn OR 'anterior circulation stroke'/syn OR Stroke OR Strokes OR ‘Cerebrovascular Accident’ OR ‘Cerebrovascular Accidents’ OR ‘CVA (Cerebrovascular Accident)’ OR ‘CVAs (Cerebrovascular Accident)’ OR ‘Cerebrovascular Apoplexy’ OR ‘Brain Vascular Accident’ OR ‘Brain Vascular Accidents’ OR ‘Cerebrovascular Stroke’ OR ‘Cerebrovascular Strokes’ OR Apoplexy OR ‘Cerebral Stroke’ OR ‘Cerebral Strokes’ OR ‘Acute Stroke’ OR ‘Acute Strokes’ OR ‘Acute Cerebrovascular Accident’ OR ‘Acute Cerebrovascular Accidents’ OR ‘Hemorrhagic Stroke’ OR ‘Ischemic Stroke’ OR ‘Lacunar Stroke’ OR ‘Brain Infarction’ |
| --- | --- |
| AND #2 | 'maladaptive behavior'/syn OR ‘Adaptive strategy’ OR ‘Adaptive Strategies’ OR ‘Compensatory Strategy’ OR ‘Compensatory Strategies’ OR ‘Behavioral Compensation’ OR ‘Behavioral Compensations’ OR ‘Behavioral Adaptation’ OR ‘Behavioral Adaptations’ |
| AND #3 | 'gait'/syn |
| OR #4 | 'sit to stand'/syn OR 'sit to stand task'/syn OR 'standing'/syn OR ‘Standing Position’ OR ‘Position, Standing’ OR ‘Standing Positions’ OR ‘sit-to-stand’ |
| OR #5 | 'sitting'/syn OR ‘Sitting Position’ OR ‘Position, Sitting’ OR ‘Sitting Positions’ OR ‘Sitting’ OR ‘Seated Position’ OR ‘Position, Seated’ OR ‘Positions, Seated’ OR ‘Seated Positions’ OR ‘stand-to-sit’ |
| OR #6 | 'stair climbing'/syn OR ‘step up’ OR ‘step down’ OR ‘step up stairs’ OR ‘Walk up stairs’ OR ‘step down stairs’ OR ‘Walk down stairs’ OR ‘walking up stairs’ OR ‘walking down stairs’ |
| OR #7 | 'turning'/syn OR Turn OR turning OR ‘180 turn’ OR pivot OR pivoting |
| OR #8 | 'body equilibrium'/syn OR ‘Posture Equilibrium’ OR ‘Posture Equilibriums’ OR ‘Posture Balance’ OR ‘Posture Balances’ OR ‘Musculoskeletal Equilibrium’ OR ‘Postural Control’ OR ‘Postural Controls’ OR ‘Posture Control’ OR ‘Posture Controls’ |
| OR #9 | 'reach'/syn OR 'reaching'/syn OR 'reaching movement'/syn OR 'reaching task'/syn OR 'reach to grasp movement'/syn OR 'manipulation'/syn OR 'grasp'/syn OR 'grasping'/syn OR 'hand function'/syn OR ‘Hand Strength’ OR ‘Strength, Hand’ OR ‘Grip Strength’ OR ‘Strength, Grip’ OR ‘Hand Grip Strength’ OR ‘Grip Strength, Hand’ OR ‘Strength, Hand Grip’ OR ‘Grip’ OR ‘Grips’ OR ‘Grasp’ OR ‘Grasps’ OR ‘Reach’ OR ‘Reaching’ OR ‘Hand Function’ OR ‘Manipulation’ |

SCOPUS:

|  | Stroke OR Strokes OR “Cerebrovascular Accident” OR “Cerebrovascular Accidents” OR “CVA” OR “CVAs” OR “Cerebrovascular Apoplexy” OR “Brain Vascular Accident” OR “Brain Vascular Accidents” OR “Cerebrovascular Stroke” OR “Cerebrovascular Strokes” OR Apoplexy OR “Cerebral Stroke” OR “Cerebral Strokes” OR “Acute Stroke” OR “Acute Strokes” OR “Acute Cerebrovascular Accident” OR “Acute Cerebrovascular Accidents” OR “Hemorrhagic Stroke” OR “Hemorrhagic Strokes” OR “Subarachnoid Hemorrhagic Stroke” OR “Subarachnoid Hemorrhagic Strokes” OR “Intracerebral Hemorrhagic Stroke” OR “Intracerebral Hemorrhagic Strokes” OR “Intracerebral Hemorrhage Stroke” OR “Intracerebral Hemorrhage Strokes” OR “Ischemic Stroke” OR “Ischemic Strokes” OR “Ischaemic Stroke” OR “Ischaemic Strokes” OR “Acute Ischemic Stroke” OR “Acute Ischemic Strokes” OR “Lacunar Stroke” OR “Lacunar Strokes” OR “Lacunar Syndrome” OR “Lacunar Syndromes” OR “Lacunar Infarction” OR “Lacunar Infarctions” OR “Lacunar Infarct” OR “Lacunar Infarcts” OR “Brain Infarction” OR “Brain Infarctions” OR “Brain Infarct” OR “Brain Infarcts” OR “Anterior Circulation Brain Infarction” OR “Brain Venous Infarction” OR “Brain Venous Infarctions” OR “Venous Brain Infarction” OR “Venous Brain Infarctions” OR “Anterior Cerebral Circulation Infarction” OR “Posterior Circulation Brain Infarction” |
| --- | --- |
| AND | “Adaptive strategy” OR “Adaptive Strategies” OR “Compensatory Strategy” OR “Compensatory Strategies” OR “Behavioral Compensation” OR “Behavioral Compensations” OR “Behavioral Adaptation” OR “Behavioral Adaptations” |
| AND | Gait OR Gaits |
| OR | “Sitting Position” OR “Sitting Positions” OR “Sitting” OR “Seated Position” OR “Seated Positions” OR “Stand-to-sit” |
| OR | “Standing Position” OR “Standing Positions” OR “Sit-to-stand” |
| OR | “Stair climbing” OR “Stair Navigation” OR “step up” OR “step down” OR “step up stairs” OR “Walk up stairs” OR “step down stairs” OR “Walk down stairs” OR “walking up stairs” OR “walking down stairs” |
| OR | Turn OR turning OR “180 turn” OR pivot OR pivoting |
| OR | “Postural Balance” OR “Posture Equilibrium” OR “Posture Equilibriums” OR “Postural Equilibrium” OR “Posture Balance” OR “Posture Balances” OR “Musculoskeletal Equilibrium” OR “Postural Control” OR “Postural Controls” OR “Posture Control” OR “Posture Controls” |
| OR | “Hand Strength” OR “Grip Strength” OR “Hand Grip Strength” OR “Grip” OR “Grips” OR “Grasp” OR “Grasps” OR “Reach” OR “Reaching” OR “Hand Function” OR “Manipulation” |

OVID:

|  | Stroke OR Strokes OR “Cerebrovascular Accident” OR “Cerebrovascular Accidents” OR “CVA” OR “CVAs” OR “Cerebrovascular Apoplexy” OR “Brain Vascular Accident” OR “Brain Vascular Accidents” OR “Cerebrovascular Stroke” OR “Cerebrovascular Strokes” OR Apoplexy OR “Cerebral Stroke” OR “Cerebral Strokes” OR “Acute Stroke” OR “Acute Strokes” OR “Acute Cerebrovascular Accident” OR “Acute Cerebrovascular Accidents” OR “Hemorrhagic Stroke” OR “Hemorrhagic Strokes” OR “Subarachnoid Hemorrhagic Stroke” OR “Subarachnoid Hemorrhagic Strokes” OR “Intracerebral Hemorrhagic Stroke” OR “Intracerebral Hemorrhagic Strokes” OR “Intracerebral Hemorrhage Stroke” OR “Intracerebral Hemorrhage Strokes” OR “Ischemic Stroke” OR “Ischemic Strokes” OR “Ischaemic Stroke” OR “Ischaemic Strokes” OR “Acute Ischemic Stroke” OR “Acute Ischemic Strokes” OR “Lacunar Stroke” OR “Lacunar Strokes” OR “Lacunar Syndrome” OR “Lacunar Syndromes” OR “Lacunar Infarction” OR “Lacunar Infarctions” OR “Lacunar Infarct” OR “Lacunar Infarcts” OR “Brain Infarction” OR “Brain Infarctions” OR “Brain Infarct” OR “Brain Infarcts” OR “Anterior Circulation Brain Infarction” OR “Brain Venous Infarction” OR “Brain Venous Infarctions” OR “Venous Brain Infarction” OR “Venous Brain Infarctions” OR “Anterior Cerebral Circulation Infarction” OR “Posterior Circulation Brain Infarction” |
| --- | --- |
| AND | “Adaptive strategy” OR “Adaptive Strategies” OR “Compensatory Strategy” OR “Compensatory Strategies” OR “Behavioral Compensation” OR “Behavioral Compensations” OR “Behavioral Adaptation” OR “Behavioral Adaptations” |
| AND | Gait OR Gaits |
| OR | “Sitting Position” OR “Sitting Positions” OR “Sitting” OR “Seated Position” OR “Seated Positions” OR “Stand-to-sit” |
| OR | “Standing Position” OR “Standing Positions” OR “Sit-to-stand” |
| OR | “Stair climbing” OR “Stair Navigation” OR “step up” OR “step down” OR “step up stairs” OR “Walk up stairs” OR “step down stairs” OR “Walk down stairs” OR “walking up stairs” OR “walking down stairs” |
| OR | Turn OR turning OR “180 turn” OR pivot OR pivoting |
| OR | “Postural Balance” OR “Posture Equilibrium” OR “Posture Equilibriums” OR “Postural Equilibrium” OR “Posture Balance” OR “Posture Balances” OR “Musculoskeletal Equilibrium” OR “Postural Control” OR “Postural Controls” OR “Posture Control” OR “Posture Controls” |
| OR | “Hand Strength” OR “Grip Strength” OR “Hand Grip Strength” OR “Grip” OR “Grips” OR “Grasp” OR “Grasps” OR “Reach” OR “Reaching” OR “Hand Function” OR “Manipulation” |

SCIENCE DIRECT:

| #1 | (Stroke) AND (“Adaptive strategy” OR “Compensatory Strategy” OR “Behavioral Compensation” OR “Behavioral Adaptation” ) AND (Gait) |
| --- | --- |
| #2 | (Stroke) AND (“Adaptive strategy” OR “Compensatory Strategy” OR “Behavioral Compensation” OR “Behavioral Adaptation” ) AND (“Sitting Position” OR “Sitting” OR “Seated Position” OR “Stand-to-sit”) |
| #3 | (Stroke) AND (“Adaptive strategy” OR “Compensatory Strategy” OR “Behavioral Compensation” OR “Behavioral Adaptation” ) AND (“Standing Position” OR “Sit-to-stand”) |
| #4 | (Stroke) AND (“Adaptive strategy” OR “Compensatory Strategy” OR “Behavioral Compensation” OR “Behavioral Adaptation” ) AND (“Stair climbing” OR “Stair Navigation” OR “Walk up stairs” OR “Walk down stairs”) |
| #5 | (Stroke) AND (“Adaptive strategy” OR “Compensatory Strategy” OR “Behavioral Compensation” OR “Behavioral Adaptation” ) AND (pivot OR pivoting) |
| #6 | (Stroke) AND (“Adaptive strategy” OR “Compensatory Strategy” OR “Behavioral Compensation” OR “Behavioral Adaptation”) AND (“Postural Balance” OR “Postural Equilibrium” OR “Musculoskeletal Equilibrium” OR “Postural Control”) |
| #7 | (Stroke) AND (“Adaptive strategy” OR “Compensatory Strategy” OR “Behavioral Compensation” OR “Behavioral Adaptation”) AND (“Hand Strength”) |

COCHRANE

|  | Stroke (MeSH) |
| --- | --- |
| AND | Adaptation, Psychological |
| OR | “Adaptive strategy” OR “Adaptive Strategies” OR “Compensatory Strategy” OR “Compensatory Strategies” OR “Behavioral Compensation” OR “Behavioral Compensations” OR “Behavioral Adaptation” OR “Behavioral Adaptations” |
| AND | Gait |
| OR | Sitting Position |
| OR | “Sitting Positions” OR “Sitting” OR “Seated Position” OR “Seated Positions” OR “Stand-to-sit” |
| OR | Standing Position |
| OR | “Standing Position” OR “Standing Positions” OR “Sit-to-stand” |
| OR | Stair Climbing |
| OR | “Stair Navigation” OR “step up” OR “step down” OR “step up stairs” OR “Walk up stairs” OR “step down stairs” OR “Walk down stairs” OR “walking up stairs” OR “walking down stairs” |
| OR | Turn OR turning OR “180 turn” OR pivot OR pivoting |
| OR | Postural Balance |
| OR | Hand Strength |
| OR | “Reach” OR “Reaching” OR “Hand Function” OR “Manipulation” |
