# Supplementary material for: Task‐Based Mapping of Compensatory Strategies and Movement Kinematics After Stroke: A Systematic Scoping Review
Source: Physiother Res Int. 2026 Apr 13;31(2):e70215. doi: 10.1002/pri.70215 (PMC13076240; doi:10.1002/pri.70215)
Supplement: Supplementary file 2 — Table S2: Description of the participants' characteristics of each study included for the sit‐to‐stand transfer task. [file PRI-31-e70215-s014.docx]

Table S2. Description of the participants’ characteristics of each study included for the sit-to-stand transfer task.

| **Author/year** | **Study type** | **N / age (years)** | **Stroke site and/or type** | **Time-based classification** | **Muscle strength** | **Spasticity** | **Assessment tools** |
| --- | --- | --- | --- | --- | --- | --- | --- |
| Engardt, Olsson, 1992 | Comparative experimental | N = 42 / 64.4 ± 7.9 (Stroke)  N = 16 / 58.7 ± 11.1 (Control) | Type: Ischemic (n = 34), hemorrhagic (n = 6), not specified (n = 2). | Subacute | Not reported | Not reported | Not reported |
| Cheng et al., 1998 | Retrospective case-control | N = 18 / 63.82 ± 6.42 (Stroke “fallers”)  N = 15 / 63 ± 6.04 (Stroke “non-fallers”)  N = 25 / 63.36 ± 8.29 (Control) | Type: Ischemic (Stroke “fallers”, n = 11; Stroke “non-fallers”, n = 10), hemorrhagic (Stroke “fallers”, n = 7; Stroke “non-fallers”, n = 5) | Chronic | Not reported | Not reported | Barthel index: 80 - 95 |
| Guerriero, Bombardi, Risaliti, 2000 | Cross-sectional observational | N = 10 / 57.6 (Stroke)  N = 7 / 55.2 (Control) | Not reported | Chronic | Not reported | Not reported | Not reported |
| Chou et al., 2003 | Cross-sectional observational | N = 40 / 60 ± 10.4 (Stroke)  N = 22 / 60.8 ± 7.4 (Control) | Site: Cortical (n = 25), subcortical (n = 15). | Chronic | Not reported | Not reported | Not reported |
| Richards et al., 2003 | Cross-sectional observational | N = 10 / 65 – 74 anos (Stroke) | Not reported | Chronic | Not reported | Not reported | RMA: 8/13 |
| Duclos, Nadeau, Lecours, 2008 | Comparative quasi-experimental | N = 18 / 50 ± 11 (Stroke)  N = 15 / 56.1 ± 10.9 (Control) | Not reported | Chronic | Not reported | CSI  Knee: 5.9 ± 3.2;  Ankle: 6.7 ± 3.7 | CMSA Leg: 4.6 ± 1.1  CMSA Foot: 3.8 ± 1.4  BBS: 51 ± 6 |
| Lecours et al., 2008 | Cross-sectional observational | N = 17 / 49.7 ± 11.3 (Stroke)  N = 15 / 56.1 ± 10.9 (Control) | Not reported | Chronic | Not reported | Not reported | CMSA Leg: 4.7 ± 1.1  CMSA Foot: 4 ± 1.5 |
| Galli et al., 2008 | Comparative experimental | N = 7 / 45.28 ± 7.58 (Stroke)  N = 13 / 34.54 ± 5.12 (Control) | Not reported | Chronic | Not reported | Not reported | Not reported |
| Na, Hwang, Woo, 2016 | Cross-sectional observational | N = 30 / 52.2 ± 9.7 (Stroke)  N = 30 / 22.7 ± 1.5 (Control) | Type: Ischemic (n = 22), hemorrhagic (n = 8) | Chronic | Not reported | Not reported | BBS: 42.9 ± 6.7  FGA: 14.6 ± 6.3  TIS: 14.2 ± 3.3 |
| Silva et al., 2017 | Cross-sectional observational | N = 18 / 59.78 ± 9.94 (Stroke)  N = 18 / 59.67 ± 9.67 (Control) | Not reported | Chronic | Not reported | Not reported | FMA Total: 80 (22)*  TIS: 16.5 (6)* |
| Mao et al., 2018 | Cross-sectional observational | N = 25 / 58.24 ± 10.46 (Stroke)  N = 17 / 57.53 ± 6.63 (Control) | Not reported | Subacute | Not reported | Not reported | Not reported |
| Darwish et al., 2019 | Cross-sectional observational | N = 30 / 45 – 60 anos (Stroke)  N = 15 / 45 – 60 anos (Control) | Not reported | Chronic | MRC Lower limbs ≥ 3 | MAS – Lower limbs: 1 – 1+ | Not reported |
| Nantawanichakorn et al., 2020 | Cross-sectional observational | N = 39 / 59.4 ± 7.4 (Stroke)  N = 10 / 60.8 ± 9.7 (Control) | Not reported | Chronic | Not reported | Not reported | Not reported |
| Franco et al., 2023 | Cross-sectional observational | N = 15 / 59.27 ± 10.29 (Stroke)  N = 15 / 58.80 ± 9.87 (Control) | Not reported | Chronic | Not reported | Not reported | 5TSTS: 20.9 ± 9.7  TIS: 17 ± 5 |

5TSTS: Five time sit-to-stand; BBS: Berg Balance Scale; CMSA: Chedoke McMaster Stroke Assessment; FGA: Functional Gait Assessment; FMA: Fugl-Meyer Assessment; MAS: Modified Ashworth Scale; MRC: Medical Research Council; RMA: Rivermead Motor Assessment; TIS: Trunk Impairment Scale.

*values presented as median (IQR).
