# Supplementary material for: Task‐Based Mapping of Compensatory Strategies and Movement Kinematics After Stroke: A Systematic Scoping Review
Source: Physiother Res Int. 2026 Apr 13;31(2):e70215. doi: 10.1002/pri.70215 (PMC13076240; doi:10.1002/pri.70215)
Supplement: Supplementary file 3 — Table S3: Description of the sit‐to‐stand transfer task in each included study. [file PRI-31-e70215-s010.docx]

Table S3. Description of the sit-to-stand transfer task in each included study.

| **Author/year** | **Task description** | **Kinematic outcomes of interest** | **Movement analysis instrument used** | **Results** |
| --- | --- | --- | --- | --- |
| Engardt, Olssen, 1992 | Position: Sitting on a chair without armrests, seat height adjusted to 100% of knee height, with ¾ of thighs on the seat and trunk upright. Feet parallel (10-18 cm), each on a force platform.  Task: Standing up in two ways: 1) habitual; 2) with weight equally distributed on the feet. Familiarization allowed. Mean of three repetitions analyzed. | Movement time;  Percentage of weight distribution. | Motion analysis system (ELITE) with markers on head and pelvis; vertical reaction force assessed with transducers coupled to force platforms, analyzed with K1-Raise software. | Longer time to stand up in both conditions, no influence of instruction used. Asymmetric distribution, with lower load on the more affected leg. Improved symmetry with instruction. |
| Cheng et al., 1998 | Position: Sitting on a chair without armrests and backrest, seat height adjusted to knee height, barefoot and wearing shorts. Feet parallel (10-15 cm), each on a force platform.  Task: Standing up at comfortable speed, remaining standing for 30 s, then sitting down. Three repetitions analyzed. | Movement duration;  Mediolateral displacement of COP (COP X);  Anteroposterior displacement of COP (COP X). | AMTI force platforms under each foot to measure anteroposterior and mediolateral balance, vertical forces, and center of pressure. | Longer movement duration and greater weight asymmetry, especially in the “fallers” stroke group. Greater COP displacement in mediolateral and anteroposterior directions. |
| Guerriero, Bombardi, Risaliti, 2000 | Position: Sitting on a chair with seat height adjusted to leg length.  Task: Standing up while keeping feet on a force platform. | Duration of flexion and extension phases;  Lateral trunk displacement in each phase;  Vertical and anteroposterior shoulder displacement in each phase;  Mediolateral displacement of COP. | ELITE system with two cameras and a force platform; three markers (acROMia and jugular notch) to analyze flexion phase (start) and extension phase (end). | Longer duration of flexion and extension phases. Greater lateral trunk displacement in both phases. Greater elevation of the more affected shoulder. Inverted movement of the more affected shoulder: posterior displacement in flexion and anterior in extension. Greater use of mediolateral forces. |
| Chou et al., 2003 | Position: Sitting barefoot, in shorts, on a chair without armrests and backrest, seat height adjusted to knee height. Feet parallel (10-15 cm), each on a force platform.  Task: Standing up at comfortable speed, remaining standing for 30 s, then sitting down. Three repetitions analyzed. | Movement duration;  Maximum vertical force;  Difference in maximum vertical force between lower limbs;  Mediolateral displacement of COP;  Anteroposterior displacement of COP. | AMTI force plataforms under each foot to measure anteroposterior and mediolateral balance, vertical forces, and center of pressure. | Longer movement duration, greater asymmetry of vertical force between limbs, and greater mediolateral COP displacement. |
| Richards et al., 2003 | Position: Sitting on a chair adjusted so that ankles, hips, and knees remained at 90º flexion.  Task: Adapted Rivermead Motor Assessment; standing up fROM a chair and maintaining standing posture for 15 s. Three repetitions performed. | Time to reach maximum extension velocity;  Task completion time;  Initial angular displacement;  Angle at point of maximum extension velocity;  Final angular displacement;  Maximum extension velocity. | Two Panasonic M3000 video cameras (sagittal plane) and markers on lower limbs; analysis performed with APAS. | Longer time to reach maximum extension velocity, longer total task duration, and lower maximum extension velocity. |
| Duclos, Nadeau, Lecours, 2008 | Position: Sitting on a chair, each thigh and foot on a force platform, arms crossed on chest.  Task: Standing up at natural speed, in two foot positioning conditions: 1) spontaneous (always first); 2) asymmetrical (less affected foot in front, at 50% of the length of the more affected foot in the stroke group; non-dominant foot in front in the control group). Two trials per position, four in total. | Lateral displacement of center of pressure, pelvis, and shoulders;  “Time-to-Contact” (TtC) of center of pressure. | Optotrak 3020 system (Northern Digital Inc.) recorded kiematic data with markers placed on feet, legs, thighs, pelvis, trunk and head. | COP, pelvis, and shoulders deviated to the less affected side, mainly before standing up, and trunk inclined to the less affected side during most of the task in spontaneous foot position. Lower lateral displacement of COP and shoulders with more affected foot behind, and reduced trunk inclination to the less afffected side, present only at the start of the task, with values close to control group. Lower minium TtC, no improvement with foot position. |
| Lecours et al., 2008 | Position: Sitting on a chair, each thigh and foot on a force platform, arms crossed on chest.  Task: Standing up, maintaining standing for 4-5 s, and sitting down. Three foot positions used: 1) spontaneous (always first, without instruction); 2) symmetrical (feet at 15º dorsiflexion); 3) asymmetrical (more affected foot posterior in stroke group; dominant foot posterior in control group, with anterior foot at 50% of posterior length, which remained at 15º dorsiflexion). Two trials per position. | Weight-bearing asymmetry;  Absolute trunk translation;  Relative trunk translation;  Lateral trunk flexion. | Chair with force sensors on thighs; two AMTI force plates (OR6-7-1000) recorded forces under each foot; Optotrak system captured infrared markers on key points of lower and upper limb. | High asymmetry in spontaneous and symmetrical foot positions, with greater load on less affected limb. Greater load balance in asymmetrical condition. Greater absolute trunk translation (spontaneous and symmetrical), toward less affected side; lower in asymmetrical condition. Greater lateral trunk flexion in spontaneous and symmetrical positions (toward less affected side); lower in asymmetrical. |
| Galli et al., 2008 | Position: Sitting barefoot, on a chair without armrests and backrest, seat height adjusted to 110% of distance fROM fibular head to lateral malleoulus. Thighs and feet on force plates.  Task: Standing up looking upward, at comfortable speed. Five repetitions. | Duration of each movement phase;  Total movement duration;  Shoulder flexion/extension ROM (tilt);  Shoulder abduction/adduction ROM (obliquity);  Shoulder external/internal rotation ROM;  Ankle dorsiflexion angle at movement onset;  Maximum ankle dorsiflexion angle. | Optoeletronic system ELITE2002 with eight cameras recorded body markers; task divided into initiation (trunk inclines), seat-off (buttocks leave seat), and stabilization (joints in extension). | Longer duration in all phases and total task. Greater shoulder ROM in flexion/extension, abduction/adduction, external/internal rotation. Greater ankle dorsiflexion angle. |
| Na, Hwang, Woo, 2016 | Task: Timed “Up and Go” test; standing up, walking three meters, turning around an obstacle, returning and sitting down. | Task duration;  Anteroposterior CM acceleration variation;  Mediolateral CM acceleration variation;  Vertical CM acceleration variation. | CM acceleration recorded with wireless triaxial acceleROMeter (G-walk) positioned at L5, analyzed with BTS G-studio software. | Longer task duration. Lower CM acceleration variation in anteroposterior, mediolateral, and vertical directions. |
| Silva et al., 2017 | Position: Sitting on a chair without armrests, seat height adjusted to 100% of leg length (knee height), with 75% of thigh on seat. Arms crossed on chest.  Task: Standing up five times, at two speeds: 1) comfortable; 2) fast. Mean of 3 repetitions analyzed. One minute rest between repetitions. | Total movement duration;  Duration of phase 1 and phase 2;  Maximum trunk forward flexion. | Six Qualisys ProReflex cameras, pressure sensor (Honeywell TruStability®), and Sony® digital camera used for analysis; three markers on each region (sternum, pelvis, thighs, legs, and feet). Movement divided into two phases: initiation to seat-off, and seat-off to end. | Longer total movement duration at both speeds. Longer phase 2 duration at self-selected speed, and phases 1 and 2 at fast speed. Greater maximum trunk flexion only at fast speed. |
| Mao et al., 2018 | Position: Sitting on chairs without backrest and armrests, seat height adjusted for hips and knees at 90º flexion. Feet on force platforms, shoulder-width apart. Arms crossed on chest.  Task: Standing up and looking upward, remaining standing for 10 s, sitting down, and repeating 10 times, with 5 s intervals. | Average curve of hip, knee, and ankle joint angles;  Time of each transition point;  Time of each movement phase;  Hip joint angle at T1, T4, T5;  Knee joint angle at T2, T4, T5;  Ankle joint angle at T3. | Vicon system (MX13, VICON Peak) with six infrared cameras recorded markers on plevis and lower limbs. Movement divided into transition points (T0: trunk flexion onset; T1: maximum hip flexion; T2: knee extension onset; T3: maximum ankle dorsiflexion; T4: onset of full hip and knee extension; T5: standing stability) and 5 phases (phase 1: T0 to before hips leave seat; phase 2: seat-off to T1; phase 3: T2 to T3; phase 4: T3 to T4; phase 5: T4 to T5). | Delay in all transition point (T1-T5) and movement phases. Phases 1 nad 4 longer. Lower maximum hip flexion at T1; hip remained flexed at T4 and T5. Lower knee flexion on more affected side ate T4 and T5. Greater anteroposterior ankle oscillation at T3. |
| Darwish et al., 2019 | Position: Sitting barefoot, on a chair without backrest, seat height adjusted to leg length (knee at 90º). Arms crossed on chest.  Task: Standing up at comfortable speed. Two repetitions performed. | Pelvic tilt angle in sitting position;  Pelvic tilt angle at task onset;  Pelvic tilt angle at task midpoint. | Pelvic tilt angles bilaterally assessed with 2D video system, using markers on trunk and lower limb. | Increased posterior pelvic til (PPT) angle in sitting positionn. Decreased anterior pelvic tilt (APT) angle at task onset. Reduction of APT at task midpoint; maintained on more affected side (right hemiparesis group) throughout the task. |
| Nantawanichakorn et al., 2020 | Position: Sitting on a chair with armrests and backrest, feet on digital load cells.  Task: Standing up under two conditions: 1) normalm (arms optional, as in daily life); 2) ideal, trying to distribute weight equally between feet, arms optional. | Minimum weight-bearing;  Maximum weight-bearing;  Mean weight-bearing;  Movement duration. | Four digital load cells (half-bridge weighing sensors) measured lower limb weight-bearing. | Asymmetry in weight-bearing, with lower load on more affected side. Reduction in asymmetry in ideal condition. Increased movement duration in ideal condition. |
| Franco et al., 2023 | Position: Sitting on a chair without armrests, seat height adjusted to 100% of leg length, with 75% of thigh on seat. Arms crossed on chest, gaze straight ahead, feet asymmetrical.  Task: Standing up at two speeds (self-selected and fast), remaining standing for 10 s, sittting down, and resting for 1 minute. Five repetitions per speed. | Maximum trunk forward flexion angle;  Duration of maximum trunk forward flexion angle;  Total time;  Duration of phase 1 of movement;  Duration of phase 2 of movement. | Optoelectronic system with six ProReflex cameras (QualisysAB) recorded markers on lower limb and trunk; data processed with Visual 3D™ v6. Task divided into phase 1 (initiation to seat-off) and phase 2 (seat-off to end). | Greater trunk forward flexion angle, with longer time to reach it, mainly at fast speed. Longer total movement time, phase 1 and phase 2 durations, at both speeds. |
