# Supplementary material for: Task‐Based Mapping of Compensatory Strategies and Movement Kinematics After Stroke: A Systematic Scoping Review
Source: Physiother Res Int. 2026 Apr 13;31(2):e70215. doi: 10.1002/pri.70215 (PMC13076240; doi:10.1002/pri.70215)
Supplement: Supplementary file 4 — Table S4: Description of the participants' characteristics of each study included for the stand‐to‐sit transfer task. [file PRI-31-e70215-s012.docx]

Table S4. Description of the participants’ characteristics of each study included for the stand-to-sit transfer task.

| **Author/year** | **Study type** | **N / age (years)** | **Stroke site and/or type** | **Time-based classification** | **Muscle strength** | **Spasticity** | **Assessment tools** |
| --- | --- | --- | --- | --- | --- | --- | --- |
| Engardt, Olsson, 1992 | Comparative experimental | N = 42 / 64.4 ± 7.9 (Stroke)  N = 16 / 58.7 ± 11.1 (Control) | Type: Ischemic (n = 34), hemorrhagic (n = 6), not specified (n = 2). | Subacute | Not reported | Not reported | Not reported |
| Cheng et al., 1998 | Retrospective case-control | N = 18 / 63.82 ± 6.42 (Stroke “fallers”)  N = 15 / 63 ± 6.04 (Stroke “non-fallers”)  N = 25 / 63.36 ± 8.29 (Control) | Type: Ischemic (Stroke “fallers”, n = 11; Stroke “non-fallers”, n = 10), hemorrhagic (Stroke “fallers”, n = 7; Stroke “non-fallers”, n = 5) | Chronic | Not reported | Not reported | Barthel index: 80 - 95 |
| Na, Hwang, Woo, 2016 | Cross-sectional observational | N = 30 / 52.2 ± 9.7 (Stroke)  N = 30 / 22.7 ± 1.5 (Control) | Type: Ischemic (n = 22), hemorrhagic (n = 8) | Chronic | Not reported | Not reported | BBS: 42.9 ± 6.7  FGA: 14.6 ± 6.3  TIS: 14.2 ± 3.3 |
| Franco et al., 2023 | Cross-sectional observational | N = 15 / 59.27 ± 10.29 (Stroke)  N = 15 / 58.80 ± 9.87 (Control) | Not reported | Chronic | Not reported | Not reported | 5TSTS: 20.9 ± 9.7  TIS: 17 ± 5 |

5TSTS: 5-time sit-to-stand; BBS: Berg Balance Scale; FGA: Functional Gait Assessment; TIS: Trunk Impairment Scale.
