# Supplementary material for: Task‐Based Mapping of Compensatory Strategies and Movement Kinematics After Stroke: A Systematic Scoping Review
Source: Physiother Res Int. 2026 Apr 13;31(2):e70215. doi: 10.1002/pri.70215 (PMC13076240; doi:10.1002/pri.70215)
Supplement: Supplementary file 5 — Table S5: Description of the stand‐to‐sit transfer task in each included study. [file PRI-31-e70215-s004.docx]

| **Author/year** | **Task description** | **Kinematic outcomes of interest** | **Movement analysis instrument used** | **Results** |
| --- | --- | --- | --- | --- |
| Engardt, Olssen, 1992 | Position: Sitting on a chair without armrests, seat height adjusted to 100% of knee height, with ¾ of thighs on the seat and trunk upright. Feet parallel (10-18 cm), each on a force platform.  Task: Standing up in two ways: 1) habitual; 2) with weight equally distributed on the feet. Familiarization allowed. Mean of three repetitions analyzed. | Movement time;  Percentage of weight distribution. | Motion analysis system (ELITE) with markers on head and pelvis; vertical reaction force assessed with transducers coupled to force platforms, analyzed with K1-Raise software. | Longer time to stand up in both conditions, no influence of instruction used. Asymmetric distribution, with lower load on the more affected leg. Improved symmetry with instruction. |
| Cheng et al., 1998 | Position: Sitting on a chair without armrests and backrest, seat height adjusted to knee height, barefoot and wearing shorts. Feet parallel (10-15 cm), each on a force platform.  Task: Standing up at comfortable speed, remaining standing for 30 s, then sitting down. Three repetitions analyzed. | Movement duration;  Mediolateral displacement of COP (COP X);  Anteroposterior displacement of COP (COP X). | AMTI force platforms under each foot to measure anteroposterior and mediolateral balance, vertical forces, and center of pressure. | Longer movement duration and greater weight asymmetry, especially in the “fallers” stroke group. Greater COP displacement in mediolateral and anteroposterior directions. |
| Na, Hwang, Woo, 2016 | Task: Timed “Up and Go” test; standing up, walking three meters, turning around an obstacle, returning and sitting down. | Task duration;  Anteroposterior CM acceleration variation;  Mediolateral CM acceleration variation;  Vertical CM acceleration variation. | CM acceleration recorded with wireless triaxial acceleROMeter (G-walk) positioned at L5, analyzed with BTS G-studio software. | Longer task duration. Lower CM acceleration variation in anteroposterior, mediolateral, and vertical directions. |
| Franco et al., 2023 | Position: Sitting on a chair without armrests, seat height adjusted to 100% of leg length, with 75% of thigh on seat. Arms crossed on chest, gaze straight ahead, feet asymmetrical.  Task: Standing up at two speeds (self-selected and fast), remaining standing for 10 s, sittting down, and resting for 1 minute. Five repetitions per speed. | Maximum trunk forward flexion angle;  Duration of maximum trunk forward flexion angle;  Total time;  Duration of phase 1 of movement;  Duration of phase 2 of movement. | Optoelectronic system with six ProReflex cameras (QualisysAB) recorded markers on lower limb and trunk; data processed with Visual 3D™ v6. Task divided into phase 1 (initiation to seat-off) and phase 2 (seat-off to end). | Greater trunk forward flexion angle, with longer time to reach it, mainly at fast speed. Longer total movement time, phase 1 and phase 2 durations, at both speeds. |

Table S5. Description of the stand-to-sit transfer task in each included study.

AMTI: Advanced Mechanical Technology, Inc.; CM: Center of mass; COP: Center of pression; LR-VF-Diff: Left-Right Vertical Force Difference.
