# Supplementary material for: Task‐Based Mapping of Compensatory Strategies and Movement Kinematics After Stroke: A Systematic Scoping Review
Source: Physiother Res Int. 2026 Apr 13;31(2):e70215. doi: 10.1002/pri.70215 (PMC13076240; doi:10.1002/pri.70215)
Supplement: Supplementary file 6 — Table S6: Description of the participants' characteristics in each included study for the gait task. [file PRI-31-e70215-s002.docx]

Table S6. Description of the participants’ characteristics in each included study for the gait task.

| **Author/year** | **Study type** | **N / age (years)** | **Stroke site and/or type** | **Time-based classification** | **Muscle strength** | **Spasticity** | **Assessment tools** |
| --- | --- | --- | --- | --- | --- | --- | --- |
| Chou et al., 2003 | Cross-sectional observational | N = 40 / 60 ± 10.4 (Stroke)  N = 22 / 60.8 ± 7.4 (Control) | Site: Cortical (n = 25), subcortical (n = 15). | Chronic | Not reported | MAS 1-2: n = 24  MAS 3-4: n = 16 | Not reported |
| Laborde et al., 2003 | Cross-sectional observational | N = 7 / 42.7 ± 18.3 (Stroke)  N = 5 / 23.5 ± 2.1 (Control) | Type: Ischemic (n = 6); hemorrhagic (n = 1). | Subacute and chronic | Not reported | MAS  Tríceps sural: 3.1 ± 1.6  Quadríceps: 1.1 ± 1.2 | Not reported |
| Richards et al., 2003 | Cross-sectional observational | N = 10 / 65 – 74 years (Stroke) | Not reported | Chronic | Not reported | Not reported | RMA: 8 |
| Titianova et al., 2003 | Cross-sectional observational | N = 25 / 55 ± 7.5 (Stroke)  N = 31 / 50.6 ± 5.2 (Control) | Type: Ischemic (n = 11), hemorrhagic (n = 14).  Site: MCA (n = 11), cortiço-subcortical (n = 9), subcortical (n = 1), internal capsule (n = 1). | Chronic | Not reported | Not reported | CMSA Lower limb: 3 – 6  mRS: 2.7 ± 0.9  Barthel index: 82.5 ± 11  FAP: 66.9 ± 17.8 |
| Kim, Eng, 2004 | Cross-sectional observational | N = 20 / 61.2 ± 8.4 (Stroke) | Type: Ischemic (n = 11), hemorrhagic (n = 7), not specifiied (n = 2). | Chronic | Not reported | MAS: 0 - 2 | Not reported |
| Chen et al., 2005 | Cross-sectional observational | N = 6 / 60 ± 7 (Stroke)  N = 6 / 61 ± 8 (Control) | Not reported | Chronic | Not reported | Not reported | FMA-LE: 21 ± 4 |
| Bensoussan et al., 2006 | Cross-sectional observational | N = 3 / 37 ± 17 (Stroke)  N = 3 / 36.6 ± 17.6 (Contro) | Type: Ischemic (n = 1), hemorrhagic (n = 2).  Site: Sylvian fissure (n = 1), internal capsule (n = 1), thalamus (n = 1). | Chronic | Not reported | MAS:  Gastrocnemius: 3.6 ± 0.5  Soleus: 3 ± 0 | FIM: 122.3 ± 2.8  Barthel index: 96.6 ± 2.8  FAC: 5 ± 0 |
| Jonsdottir et al., 2009 | Cross-sectional observational | N = 39 / 58 (28 – 79) (Stroke)  N = 14 / 60 (34 – 75) (Control) | Type: Ischemic (n = 26), hemorrhagic (n = 13). | Late subacute and chronic | Not reported | Not reported | Not reported |
| Balasubramanian et al., 2010 | Cross-sectional observational | N = 39 / 60.21 ± 12.32 (Stroke)  N = 20 / 66.15 ± 10.03 (Control) | Not reported | Chronic | Not reported | Not reported | Not reported |
| Roerdink, Beek, 2011 | Cross-sectional observational | N = 10 / 36 (46 – 78 years) (Stroke)  N = 9 / 69 (60 – 78 years) (Control) | Not reported | Late subacute and chronic | Not reported | Not reported | FMA Total: 84.6 ± 27.7  FMA-Arm: 49.1 ± 20.5  FMA-LE: 24.9 ± 6.6  FMA-Balance: 10.6 ± 2.7  BBS: 52.6 ± 3.7  Motricity index: 72.1 ± 22.8 |
| Carmo et al., 2012 | Cross-sectional observational | N = 14 / 53 ± 10.3 (Stroke)  N = 7 / 49.8 ± 4 (Control) | Not reported | Chronic | Not reported | MAS: 1 | FMA Total: 49.3 ± 20.7  BBS: 50 |
| Hacmon et al., 2012 | Cross-sectional observational | N = 11 / 62 ± 11.1 (Stroke)  N = 11 / 68 ± 4.6 (Control) | Not reported | Chronic | Not reported | Not reported | CMSA postural: 5.6 ± 0.7  CMSA leg: 5.9 ± 0.9  CMSA foot: 5.5 ± 1.2  CMSA arm: 5.3 ± 1.8  CMSA hand: 4.8 ± 2.2  BESTest: 87.6 ± 9.8  FGA: 23.3 ± 3.5 |
| Polese et al., 2012 | Comparative experimental | N = 19 / 56.5 ± 7.4 (Stroke) | Not reported | Chronic | Knee extensor (kg): 16 ± 10 | Ashworth scale: 1.6 ± 1 | FMA-LE: 23 (15 – 31)  BBS: 48 (39 – 56) |
| Mazuquin et al., 2014 | Case-control | N = 2 / 60 ± 4.2 (Stroke)  N = 1 / 56 (Control) | Type: Ischemic (n = 2).  Site: Basilar artery (n = 1), MCA (n = 1). | Chronic | Not reported | Not reported | Barthel index: 92.5 ± 10.6 |
| Stanhope et al., 2014 | Cross-sectional observational | N = 8 / 63.6 ± 11.9 (household stroke)  N = 7 / 64.3 ± 9.5 (limited community stroke)  N = 6 / 54.9 ± 8.3 (community stroke) | Not reported | Chronic | Not reported | Not reported | FMA-LE:  Household: 19 ± 5.6  Limited comunnity: 22 ± 6.9  Community: 25 ± 2.1 |
| Bonnyaud et al., 2016 | Cross-sectional observational | N = 29 / 54.2 ± 12.2 (Stroke)  N = 25 / 51.6 ± 8.7 (Control) | Not reported | Chronic | MRC:  Hip flexors: 3,9 ± 0,4  Hip extensors: 3.2 ± 0.9  Knee flexors: 3.1 ± 0.8  Knee extensors:  4.6 ± 0.4  Ankle dorsiflexors: 3.2 ± 1.3  Ankle plantarflexors: 1.7 ± 1.2 | MAS:  Quadriceps: 1 ± 1  Hamstrings: 0.2 ± 0.4  Triceps surae: 0.9 ± 1 | Not reported |
| Kim et al., 2016 | Cross-sectional observational | N = 5 / 61.2 ± 10 (Stroke)  N = 5 / 29 ± 2.9 (Control) | Not reported | Chronic | Not reported | Not reported | Not reported |
| Titus et al., 2018 | Cross-sectional observational | N = 17 / 56.3 ± 9.5 (Stroke) | Not reported | Subacute and chronic | Not reported | Not reported | Not reported |
| Belyaeva et al., 2020 | Cross-sectional observational | N = 15 / 61.7 ± 8.7 (Supratentorial stroke)  N = 9 / 59.9 ± 7.1 (Infratentorial stroke)  N = 11 / 56.1 ± 8.9 (Control) | Type: Ischemic (n = 24)  Site: MCA (Supratentorial stroke, n = 15; Infratentorial stroke, n = 0), cerebellum (Supratentorial stroke, n = 0; Infratentorial stroke, n = 9) | Subacute | Supratentorial stroke MRC:  Upper limb: 3,4 ± 0,9  Lower limb: 4,1 ± 0,7  Infratentorial MRC:  Upper limb: 3,7 ± 0,7  Lower limb: 4,0 ± 0,8 | MAS: 0.5 ± 0.7 | Supratentorial stroke NIHSS: 6.4 ± 0.6  Infratentorial stroke NIHSS: 6.1 ± 0.8 |
| Wang et al., 2020 | Retrospective cohort | N = 38 / 56.4 ± 8.8 (0.5 – 1.4 km/h stroke)  N = 38 / 56.7 ± 8.2 (0.5 – 1.4 km/h controle)  N = 33 / 54.7 ± 11.5 (1.5 -2.4 km/h stroke)  N = 33 / 54.8 ± 11.5 (1.5 -2.4 km/h control)  N = 29 / 53 ± 12.5 (2.5 – 3.4 km/h stroke)  N = 29 / 52 ± 12.4 (2.5 – 3.4 km/h control)  N = 18 / 50.7 ± 12.5 (3.5 – 4.4 km/h stroke)  N = 18 / 50.4 ± 12.5 (3.5 – 4.4 km/h control)  N = 12 / 48.8 ± 13.1 (4.5 – 5.5 km/h stroke)  N = 12 / 48.3 ± 13.1 (4.5 – 5.5 km/h control) | Type: Ischemic  0.5 – 1.4 km/h stroke, n = 25  1.5 – 2.4 km/h stroke, n = 20  2.5 – 3.4 km/h stroke, n = 16  3.5 – 4.4 km/h stroke, n = 15  4.5 – 5.5 km/h stroke, n = 10  Hemorrhagic  0.5 – 1.4 km/h stroke, n = 13  1.5 – 2.4 km/h stroke, n = 13  2.5 – 3.4 km/h stroke, n = 13  3.5 – 4.4 km/h stroke, n = 3  4.5 – 5.5 km/h stroke, n = 2 | Subacute and chronic | Not reported | Not reported | SIAS:  0.5 – 1.4 km/h: 7.4 ± 3.1  1.5 – 2.4 km/h: 8.8 ± 2.7  2.5 – 3.4 km/h: 10.6 ± 2.8  3.5 – 4.4 km/h: 12.7 ± 2.3  4.5 – 5.5 km/h: 13.3 ± 1.4 |
| Haruyama et al., 2021 | Cross-sectional observational | N = 42 / 52.3 ± 13 (Stroke)  N = 10 / 51.9 ± 14.8 (Control) | Not reported | Chronic | Not reported | MAS quadriceps: 0 (0, 1)*  MAS hamstrings: 0 (0, 1)*  MAS gastrocnemius: 1 (0.25, 2)* | FMA-LE: 25.9 ± 3.8  GAIT: 23.3 ± 8.9 |

BESTest: Balance Evaluation Systems Test; BBS: Berg Balance Scale; CMSA: Chedoke-McMaster Stroke Assessment; FAC: Functional Ambulation Category; FAP: Functional Ambulation Profile; FGA: Functional Gait Assessment; FIM: Functional Independence Measure; FMA-LE: Fugl-Meyer Assessment – Lower Extremity; FMA total: Fugle-Meyer Assessment – total score; GAIT: Gait Assessment and Intervention Tool; MAS: Modified Ashworth Scale; MCA: middle cerebral artery; MRC: Medical Research Council; MiniBESTest: Mini Balance Evaluation Systems Test; mRS: Modified Rankin Scale; NIHSS: National Institutes of Health Stroke Scale; RMA: Rivermead Motor Assessment; SIAS: Stroke Impairment Assessment Set; TIS: Trunk Impairment Scale.

*values presented as median (25^th^, 75^th^ percentiles)
