# Supplementary material for: Task‐Based Mapping of Compensatory Strategies and Movement Kinematics After Stroke: A Systematic Scoping Review
Source: Physiother Res Int. 2026 Apr 13;31(2):e70215. doi: 10.1002/pri.70215 (PMC13076240; doi:10.1002/pri.70215)
Supplement: Supplementary file 7 — Table S7: Description of the gait task in each included study. [file PRI-31-e70215-s009.docx]

**Table S7.** Description of the gait task in each included study.

| **Author/year** | **Task description** | **Kinematic outcomes of interest** | **Movement analysis instrument used** | **Results** | |
| --- | --- | --- | --- | --- | --- |
| Chou et al., 2003 | Task: Walk in a 10-meter corridor at a comfortable pace. Three recording were used for analysis. | Gait speed;  Cadence;  Step length;  Stride time;  Percentage of single support phase;  Percentage of double support phase;  Symmetry index. | Vicon 370 system with high-resolution cameras (Oxford metrics, Oxford, UK) and three AMTI force plates; markers positioned on lower-limb anatomical landmarks. | Reduced gait speed, cadence, and step length. Increased stride time. Lower percentage of single support phase and prolonged double support phase. Lower symmetry index. | |
| Laborde et al., 2003 | Task: Walk barefoot along a 10-meter track inside the Henry-Gabrielle Hospital laboratory. Gait was to be performed spontaneously and naturally. | Marker positions at the hip, knee, and ankle joints;  Types of gait identified. | 3D Vicon system and 13 markers positioned on anatomical landmarks of both lower limbs. Movement was analyzed only in the sagittal plane. | Three gait types identified: 1. Near normal, with knee flexion > 45º and hip hiking < 30 mm; 2. Hip hiking > 40 mm due to knee flexion <30º; 3. Hip hiking < 10 mm and ankle elevation < 30 mm. | |
| Richards et al., 2003 | Task: Adapted fROM the Rivermead Motor Assessment. Walk straight for 10 meters, unaided, at a comfortable pace. | Time to knee flexion in mid-stance;  Time to maximum knee flexion in swing;  Time to knee extension in mid-stance;  Stride time;  Angle at initial heel contact;  Maximum knee flexion angle in mid-stance;  Maximum knee flexion angle in swing;  Maximum knee extension angle in mid-stance;  Angular velocity of flexion in stance and swing;  Angular velocity of extension in stance and swing. | Two Panasonic M3000 video cameras positioned in the sagittal plane and markers placed on the thighs and shanks. Data were analyzed using APAS. | Greater angle at initial heel contact. Lower maximum knee extension angle in mid-stance. Lower angular velocities of flexion and extension in both stance and swing. | |
| Titianova et al., 2003 | Task: Walk twice along a 10.5-m corridor at a comfortable pace. | Gait speed;  Number of steps;  Cadence;  Step length;  Stride length;  Step width (heel-to-heel base of support);  Cycle time;  Percentage of swing phase;  Percentage of stance phase;  Percentage of double support;  Double support time. | Portable GAITRite system, consisting of a 4.57 m electronic walkway with an active recording area of 3.66 x 0.61 m. The system requires at least one complete stride of each side within the recording area. | Reduced gait speed, cadence, step and stride length, and percentage of swing phase on the less affected side. Increased number of steps, base of support, cycle time, percentage of double support and of single-limb stance on the less affected side, and total double support time. | |
| Kim, Eng, 2004 | Task: Walk 8 meters at habitual speed over three force platforms, without orthoses. Use of walking aids was allowed. | Hip, knee, and ankle ROM in the sagittal, frontal, and transverse plane;  Peak hip flexion, extension, abduction, and internal/external rotation;  Peak knee flexion;  Peak ankle plantarflexion and inversion. | Optoelectronic sensor (Northern Digital, Waterloo, Canada) tracking marker movement on each lower limb. | Lower hip, knee, and ankle ROM on the more affected side in the sagittal plane. Greater ankle ROM on the more affected side in the frontal plane. Greater knee and ankle ROM on the more affected side in the transverse plane. Lower peaks of hip flexion, extension, abduction, and rotation, knee flexion, and ankle plantarflexion on the more affected side. | |
| Chen et al., 2005 | Task: Walk on a treadmill at a comfortable, previously defined speed. Control group walked at the same speed as the paired stroke participant; use of orthoses and walking aids allowed. | Speed;  Cadence;  Stride time;  Stride length;  Step width;  Swing time;  Step length. | Qualisys motion analysis system (Qualysis Inc., East Windsor, CT) and five ProReflex digital cameras. Markers were placed on the runk, pelvis, thighs, shanks and feet. | Greater step width. Longer swing time in the more affected limb. Greater magnitude of step-length asymmetry. |  |
| Bensoussan et al., 2006 | Position: Barefoot, each foot on a force plate.  Task: Walk at self-selected speed. Ten trials for each limb that initiated gait. | Duration of P1;  Duration of P2;  Duration of P3;  Total gait initiation time;  Ankle ROM in the sagittal plane;  Knee elevation in swing;  Foot clearance;  Initial contact with the ground. | ELITE optoeletronic system (BTS spa, Milan, Italy) with six cameras positioned behind and in front of the individual and anatomical markers on the lower limbs. Two AMTI force plates (Advanced Mechanical Technology Inc., Watertown, MA, USA). Three gait-initiation phases defined for analysis: a postural phase (P1), a single-support phase (P2), and a double-support phase (P3). | Longer P1 and shorter P2 when the more affected leg was used as support. Longer total gait initiation time. Greater ankle ROM in the sagittal plane and greater knee elevation ins wing on the more affected sidie. Initial contact performed with foot-flat. | |
| Jonsdottir et al., 2009 | Task: Walk five times along the proposed path at a comfortable pace, without orthoses or walking aids. They were also to perform a single lap at a faster speed. | Cadence;  Stride length. | ELITE optolectronic system (BTS, Italy). Markers were positioned following a predefined arrangement. | Higher cadence, especially in the slower participants. Reduction of stride length in one-third of participants at preferred and fast speeds. | |
| Balasubramanian et al., 2010 | Position: Use of safety harnesses attached to the ceilling, without partial body-weight support. Accompanied by physiotherapists.  Task: First, walk two to three times over the GAITRite surface (3.65 m) at a comfortable pace. Then, walk three times on a split-belt treadmill (TECMACHINE) at a self-selected speed. Recording for 30 seconds. | Anteroposterior foot placement;  Mediolateral foot placement;  Global foot placement (step length and width);  Step-length asymmetry;  Paretic limb propulsion (forward progression);  Percentage of weight on the paretic limb. | Instrumented 3.65 m surface (GAITRite). Markers were used, and trajectories were fitted to an eigth-segment musculoskeletal model generated with SIMM (MusculoGraphics, Inc.), consisting of a trunk, pelvis, and legs for each subject. | More anterior and lateral placement of the more affected foot relative to the pelvis, especially at slower speed. Step-length asymmetry between sides. Greater load on the foot when placed more laterally. | |
| Roerdink, Beek, 2011 | Position: Use of safety harnesses, with two people near the treadmill to ensure safety.  Task: Walk for 90 seconds on a treadmill at a self-selected speed previously determined by the 10-meter walk test. | Step length;  Trunk progression;  Anterior foot placement. | Three-dimensional motion system with markers (Optotrak 3020, Northern Digital Inc., Waterloo, Canada). Markers were placed on the pelvis and heels. | Greater trunk progression during the step of the more affected side. Variation in anterior foot placement and step length, without a consistent pattern. | |
| Carmo et al., 2012 | Task: Walk at a comfortable speed. At least one gait cycle for each lower limb was recorded. | Step length;  Step width;  Stride legnth;  Stride time;  Gait speed;  Cadence;  Duration of single support, double support and swing phase;  Maximum, minimum, mean angles and ROM of ankle, knee, hip, shoulder, and elbow in sagittal, frontal and transverse planes. | DVideo kinematic analysis system with four Gen-lock Basler video cameras (Model A602fc, Germany) and markers placed on participants’s bodies. | Reduced step length, stride length, and gait speed. Increased swing time and reduced single and double support times on the more affected side. In the sagittal plane, reduced ankle ROM (lower maximum extension), knee ROM (lower maximum flexion), hip and shoulder ROM (lower maximum extension); and increased elbow ROM (higher mean flexion). In the frontal plane, reduced ROM of ankle, knee and hip; and increased shoulder ROM (higher mean abduction). In the transverse plane, reduced ROM of ankle, knee, and shoulder (lower external rotation), and elbow; and increased hip ROM. | |
| Hacmon et al., 2012 | Position: Use of a safety harness attached to the ceiling, without movement restriction or weight support.  Task: Walk at two speeds: stroke group at habitual speed and 20% faster; control group at habitual speed and 20% slower. A metronome was used to maintain cadence. Four 30-second trials were performed. | Step length;  Step width;  Cadence;  Gait speed;  Thoracic ROM;  Pelvic ROM;  Sagittal arm movement. | Motion capture system with 12 cameras (Vicon-512, Los Angeles, California, USA) and markers positioned on the runk and lower limbs. | Shorter and wider steps. Higher cadence and lower gait speed. Greater thoracic ROM. Lower pelvic rotation ROM and lower sagittal movement of the more affected arm. | |
| Polese et al., 2012 | Task: Corridor walking with force platforms under 4 conditions: 1. With an assistive device, at fast speed; 2. With an assistive device, at comfortable speed; 3. Without an assistive device, at fast speed; 4. Without an assistive device, at comfortable speed. Three recordings for each condition. | Gait speed;  Maximum ankle dorsiflexion angle;  Maximum knee flexion angle;  Maximum hip extension angle. | Qualisys Pro-Reflex-MCU 240 system (Qualisys Medical AB, Gothenburg, Sweden) with eight cameras and markers on the lower limbs. | Increased gait speed with cane use, at both speeds. | |
| Mazuquin et al., 2014 | Task: Walk three times in a 10-meter corridor at a speed they considered “comfortable”. | The following variables were analyzed for the hip (H), knee (K) and ankle (A) joints:  Joint angle at initial contact (H1, K1, A1);  Maximum flexion angle in loading response (H2, K2, A2);  Maximum extension angle in stance (H3, K3, A3);  Joint angle at toe-off (H4, K4, A4);  Maximum flexion angle in swing (H5, K5, A5);  Total ROM in the sagittal plane (H6, K6, A6). | Inertial sensor system (MVN, Xsens Tech®, Enschede, Netherlands) with 17 inertial sensors on participants’ bodies, connected to an integrator system (Xbus Master, Xsens Tech®, Enschede, Netherlands). | Paitents A and B showed lower hip flexion and extension angles in stance and swing. Patient B showed greater knee flexion at initial contact and loading response and maintained a flexed position throughout stance; and lower ankle dorsiflexion in stance and a reduced total dorsiflexion ROM. | |
| Stanhope et al., 2014 | Position: Use of a safety harness connected to an overhead support coupled to a split-belt treadmill (Bertec Corp., Clumbus, OH, USA), without providing weight support.  Task: Walk on a treadmill for 2 minutes, twice, at a speed previously defined by a walking test. | Peak ankle dorsiflexion angle;  Peak knee flexion angle;  Peak pelvic tilt angle;  Peak hip abduction angle;  Toe displacement. | Gait analysis system with eight cameras and 42 markers positioned on the trunk, pelvis, and lower limbs. | Greater ankle dorsiflexion, greater knee flexion, and lower pelvic tilt during gait for those wight higher gait speed. | |
| Bonnyaud et al., 2016 | Task: Timed “Up and Go” test: stand up fROM a chair, walk 3 meters, go around an object, return, and sit down again. The stroke group pivoted toward the more affected limb, and the control group toward the dominant limb. The test was performed 3 times at a comfortable speed. | Time of the TUG “Go” and “Return” phases;  Cadence;  Step width;  Step length;  Percentage of single-limb stance;  Percentage of swing phase;  Peak hip flexion/extension;  Peak knee flexion/extension;  Peak ankle dorsiflexion/plantarflexion;  Maximum ankle dorsiflexion in swing. | Motion system with 8 cameras (Motion Analysis Corporation) with 34 markers. The test was divided into three moments for analysis: “Go”, “Turn” and “Return”. | Longer times in the “Go” and “Return” phases. Reduction in cadence, step length, percentage of single-limb stance and swing. Greater step width. Lower peak hip, knee, and ankle flexion/extension on the more affected side. Greater peak knee flexion on the less affected side. Reduced maximum ankle dorsiflexion in swing on the more affected side. | |
| Kim et al., 2016 | Task: Walk at a comfortable speed along a 10-meter corridor inside a gait laboratory. Task performed five times. | Ankle dorsiflexion/plantarflexion ROM;  Knee flexion/extension ROM;  Hip flexion/extension ROM;  Pelvic til, rotation, and obliquity ROM;  Lateral pelvic displacement;  Step length;  Stride length;  Gait speed;  Percentage duration of stance phase. | Vicon motion capture system (Nexus, Oxford, UK) with eight high-speed optical cameras (Vicon, Oxford, UK) capturing the positions of 16 markers attached to participants’ bodies. | Reduced ankle dorsiflexion/plantarflexion ROM, knee flexion/extension ROM, and hip flexion/extension ROM. Greater pelvic tilt and rotation ROM and lateral pelvic displacement. Reduced step length, stride length, and gait speed. Longer stance phase duration. | |
| Titus et al., 2018 | Task: Walk at comfortable speed for 10 meters in a 30-meter laboratory, 6 times. | Peak trunk angle at initial contact, toe-off, and over the entire gait cycle for sagittal, frontal, and transverse planes. | T-10 Vicon system with eight cameras (Vicon Motion System Ltd, Oxford, UK) and 22 markers positioned on pelvis and lower limbs. | Anterior trunk lean on both sides. Lateral lean toward the more affected side. Posterior rotation toward the more affected side. | |
| Belyaeva et al., 2020 | Task: Perform five consecutive gait cycles along a walkway, barefoot and at a comfortable pace. | Lateral pelvic tilt (sagittal plane);  Oblique pelvic tilt (frontal plane);  Hip flexion;  Hip extension;  Knee flexion;  Knee extension;  Ankle dorsiflexion;  Ankle plantarflexion. | Physiomed Smart motion analysis system (Physiomed, Germany, Davis protocol), equipped with a high-precision, high-resolution SMART-D digital optoelectronic system for analysis of all movement types. Filming was done with 13 video cameras. | Excessive lateral and anterior pelvic tilt on the more affected side, increased hip internal rotation, reduced ankle plantarflexion in patients with supratentorial stroke. Increased anterior tilt and reduced lateral tilt of the pelvis on the more affected side, excessive hip flexion, hip and knee extension, and insufficient ankle dorsiflexion and plantarflexion in both legs. | |
| Wang et al., 2020 | Task: Walk for 20 seconds at a comfortable speed, previously defined by a 10-meter walk test. Analysis of 5 complete gait cycles. | Step length;  Stride length;  Cadence;  Stance time;  Swing time;  Double support time. | Three-dimensional motion capture system with a force assessment plate (KinemaTracer, Kissei Comtec Co., Ltd., matsumoto, Japan) and 12 markers positioned on the shoulder, pelvis, and lower limbs. | Increased step and stride length and reduced cadence in those slower participants. Longer single and double support times on both sides, mainly in those who were slower. Increased swing time on the more affected side and reduced on the less affected side. | |
| Haruyama et al., 2021 | Task: Walk barefoot along a 10-meter corridor at a self-selected speed. Gait performed 8 times. | Minimum pelvis-hallux distance (PTDmin);  Time to reach PTDmin;  Peak foot clearance in the frontal plane;  Time to foot clearance in the frontal plane;  Peak hip tilt angle;  Peak hip circumduction angle;  Peak foot clearance in the sagittal plane;  Time to peak foot clearance in the sagittal plane;  Peak hip flexion angle;  Peak knee flexion angle;  Peak ankle dorsiflexion angle. | Motion capture system with eight cameras (Vicon Vantage V8; Vicon Motion Systems, Oxford, UK). Sixteen markers were placed on the lower limbs. | Lower PTDmin on the more affected side and higher on the less affected side. Longer time to reach PTDmin on the more affected side. Higher peak foot clearance in the frontal plane on the more affected side, occurring earlier, and lower peak in the sagittal plane. Greater hip tilt and circumduction angles on the more affected side. Lower knee flexion on the more affected side. | |

AMTI: Advanced Mechanical Technology, Inc; APAS: Ariel Performance Analysis System; BTS: Bioengineering Technology and Systems; MVN: Motion Visual Network; ROM: Range of movement; SIMM: Software for Interactive Musculoskeletal Modeling; TUG: Timed Up and Go test; UK: United Kingdom; USA: United States of America; VHS: Video Home System.
