# Supplementary material for: Task‐Based Mapping of Compensatory Strategies and Movement Kinematics After Stroke: A Systematic Scoping Review
Source: Physiother Res Int. 2026 Apr 13;31(2):e70215. doi: 10.1002/pri.70215 (PMC13076240; doi:10.1002/pri.70215)
Supplement: Supplementary file 8 — Table S8: Description of the participants' characteristics in each included study for the climb up and down stairs task. [file PRI-31-e70215-s011.docx]

**Table S8.** Description of the participants’ characteristics in each included study for the climb up and down stairs task.

| **Author/year** | **Study type** | **N / age (years)** | **Stroke site and/or type** | **Time-based classification** | **Muscle strength** | **Spasticity** | **Assessment tools** |
| --- | --- | --- | --- | --- | --- | --- | --- |
| Novak, Brouwer, 2013 | Cross-sectional observational | N = 10 / 60.1 ± 10.3 (Stroke)  N = 10 / 59.4 ± 8.7 (Control) | Not reported | Chronic | Not reported | Not reported | CB&M: 53.9 ± 20.3 |
| Goyal et al., 2023 | Cross-sectional observational | N = 5 / 59.0 ± 7.6 (Stroke)  N = 5 / 60.4 ± 5.0 (Control) | Not reported | Chronic | Not reported | Not reported | FMA-LE: 20.4 ± 5  ABC: 84.3 ± 13.9 |

ABC: Activities-specific Balance Confidence Scale; CB&M: Community Balance and Mobility Scale; FMA-LE: Fugl-Meyer Assessment – Lower Extremity.
