# Supplementary material for: Task‐Based Mapping of Compensatory Strategies and Movement Kinematics After Stroke: A Systematic Scoping Review
Source: Physiother Res Int. 2026 Apr 13;31(2):e70215. doi: 10.1002/pri.70215 (PMC13076240; doi:10.1002/pri.70215)
Supplement: Supplementary file 9 — Table S9: Task description for the stair ascent and descent task in each included study. [file PRI-31-e70215-s007.docx]

**Table S9.** Task description for the stair ascent and descent task in each included study.

| **Author/year** | **Task description** | **Kinematic outcomes of interest** | **Movement analysis instrument used** | **Results** |
| --- | --- | --- | --- | --- |
| Novak, Brouwer, 2013 | Task: Ascend and descend a 4-step staircase, each step 15 cm high, 26 cm deep, and 56 cm wide, at self-selected pace, using a step-over-step pattern. Two conditions: with handrail support and without support. | Ankle dorsiflexion/plantarflexion and inversion/eversion ROM;  Knee flexion/extension ROM;  Hip flexion/extension and abduction/adduction ROM;  Stance time;  Cadence. | Optotrak 3020 motion-capture system with two sensors positioned on the lower limbs; AMTI OR6-6 force platform. | Lower ankle dorsiflexion/plantarflexion ROM on the more affected side, both in ascent and descent. Greater knee and hip flexion/extension ROM on the less affected side, in ascent and descent. Longer stance time during ascent. Lower cadence under both conditions. |
| Goyal et al., 2023 | Position: Standing on two individual force plates located behind the step platforms, equipped with a safety harness connected to a passive ceiling rail system. Steps 10.2 cm high, designed to cover the full surface of two force plates.  Task: Ascend the step naturally, avoiding use of the hands. Twelve trials were recorded per leading leg. | Hip agduction during swing;  Hip flexion during swing;  Knee flexion during swing;  Pelvic obliquity during swing. | Motion capture system with 12 cameras (Qaulisys, Gothenburg, Sweden) and markers positioned on the sternum, pelvis, thigh, shank and foot, plus four-maker clusters on thigh and shank. | Lower hip and knee flexion angles and presence of plevic obliquity during swing of the less affected limb. |

AMTI: Advanced Mechanical Technology, Inc.
