# Supplementary material for: Task‐Based Mapping of Compensatory Strategies and Movement Kinematics After Stroke: A Systematic Scoping Review
Source: Physiother Res Int. 2026 Apr 13;31(2):e70215. doi: 10.1002/pri.70215 (PMC13076240; doi:10.1002/pri.70215)
Supplement: Supplementary file 10 — Table S10: Description of the participants' characteristics in each included study for the turning task. [file PRI-31-e70215-s001.docx]

**Table S10.** Description of the participants’ characteristics in each included study for the turning task.

| **Author/year** | **Study type** | **N / age (years)** | **Stroke site and/or type** | **Time-based classification** | **Muscle strength** | **Spasticity** | **Assessment tools** |
| --- | --- | --- | --- | --- | --- | --- | --- |
| Lamontagne; Fung, 2009 | Cross-sectional observational | N = 8 / 62 ± 4 (Stroke)  N = 7 / 65 ± 7 (Control) | Site: MCA (n = 8) | Subacute and Chronic | Not reported | Not reported | CMSA postural: 4.2 ± 1.0  CMSA leg: 3.8 ± 1.7  CMSA foot: 2.1 ± 1.8 |
| Hollands et al., 2010 | Cross-sectional observational | N = 9 / 59.1 ± 9.6 (Stroke without falls history)  N = 9 / 60.8 ± 11.7 (Stroke with falls history)  N = 18 / idade não reportada (Controle) | Not reported | Chronic | Not reported | Not reported | FMA-LE: 29.5 ± 4.8  BBS: 54.8 ± 1.6  TUG: 30.6 ± 11.2 |
| Ahmad et al., 2014 | Cross-sectional observational | N = 10 / 66 ± 10 (Stroke)  N = 10 / 65 ± 8 (Control) | Not reported | Chronic | Not reported | Not reported | Barthel index: 91 ± 11  BBS: 47 ± 6 |
| Bonnyaud et al., 2016 | Cross-sectional observational | N = 29 / 54.2 ± 12.2 (Stroke)  N = 25 / 51.6 ± 8.7 (Control) | Not reported | Chronic | MRC:  Hip flexors: 3,9 ± 0,4  Hip extensors: 3.2 ± 0.9  Knee flexors: 3.1 ± 0.8  Knee extensors:  4.6 ± 0.4  Ankle dorsiflexors: 3.2 ± 1.3  Ankle plantarflexors: 1.7 ± 1.2 | MAS:  Quadriceps: 1 ± 1  Hamstrings: 0.2 ± 0.4  Triceps surae: 0.9 ± 1 | Not reported |
| Liang; Chen; Lee, 2018 | Cross-sectional observational | N = 30 / 58 ± 11 (Stroke)  N = 30 / 54 ± 24 (Control) | Type: Ischemic (n = 18), hemorrhagic (n = 12). | Late subacute and chronic | Not reported | Not reported | FAT: 22 ± 7  CMSA leg: 5 ± 1  CMSA foot: 4 ± 2  BBS: 47 ± 7  10MWT: 18 ± 15  TIS: 18 ± 3 |
| Abdollahi et al., 2021 | Cross-sectional observational | N = 5 / 59.6 ± 7.3 (Stroke)  N = 5 / 67.6 ± 7.6 (Control) | Not reported | Chronic | Not reported | Not reported | Not reported |
| Abdollahi et al., 2022 | Cross-sectional observational | N = 14 / 69 ± 8.4 (Stroke)  N = 14 / 74 ± 8.7 (Control) | Not reported | Chronic | Not reported | Not reported | Not reported |
| Soangra et al., 2021 | Cross-sectional observational | N = 14 / 74 ± 8.7 (Stroke)  N = 14 / 69 ± 8.4 (Control) | Not reported | Chronic | Not reported | Not reported | Not reported |

10MWT: 10-meter walk test; BBS: Berg Balance Scale; CMSA: Chedoke-McMaster Stroke Assessment; FAT: Frenchay Arm Test; FMA-LE: Fugl-Meyer Assessment – Lower Extremity; TIS: Trunk Impairment Scale; TUG: Timed Up and Go test.
