# Supplementary material for: Task‐Based Mapping of Compensatory Strategies and Movement Kinematics After Stroke: A Systematic Scoping Review
Source: Physiother Res Int. 2026 Apr 13;31(2):e70215. doi: 10.1002/pri.70215 (PMC13076240; doi:10.1002/pri.70215)
Supplement: Supplementary file 11 — Table S11: Task description for the turning task in each included study. [file PRI-31-e70215-s015.docx]

**Table S11.** Task description for the turning task in each included study.

| **Author/year** | **Task description** | **Kinematic outcomes of interest** | **Movement analysis instrument used** | **Results** |
| --- | --- | --- | --- | --- |
| Lamontagne, Fung, 2009 | Task: Walk in a corridor (9 m x 0.9 m) and make a 90º turn (left/right) according to a light arrow activated 1 meter before the intersection. The start was always with the preferred lower limb. Two speeds: preferred and slower (equivalent to that of individuals with hemiparesis). | Horizontal orientation of gaze, head, thorax, pelvis, and feet;  Onset timing of body-segment reorientation;  Gait speed during turns;  Sequence of body-segment reorientation. | Vicon-512 motion-analysis system with 6 cameras and 38 markers for kinematic data, and an EyeLink-II video-oculography system for eye movements. | Synchronized yet-discoordinated, caudo-rostral reorientation (pelvis > thorax > head > gaze), with excessive and erratic head and gaze movements, particularly in slower participants and when turning toward the less affected side. Reorientation onset after the intersection, mainly in slower participants. Lower gait speed during turns. |
| Hollands et al., 2010 | Task: Timed “Up and Go” test: stand up, walk 3 meters, turn 180º, return, and sit down. Twenty trials (10 turns toward the more affected side, 10 toward the less affected side), randomized order. Fast and safe execution. | Time to perform the turn;  Tumber of steps to perform the turn;  Latency of axial-segment reorientation;  Head and trunk preview distance; Coordination among axial segments. | Motion-capture system with 13 Vicon cameras tracking 32 reflective markers positioned bilaterally on anatomical landmarks (head, sternum, arms, wrists, hips, thighs, shanks, and feet). | Longer time to perform the turn in patients with a history of falls. Head reorientation closer to the turning point when turning toward the less affected side. |
| Ahmad et al., 2014 | Task: Turn in place to face a target line (45º, 90º, or 135º, right/left) when the central light turned off. Two conditions: predictable (known direction) and unpredictable (random direction). Twelve turns (6 per condition) repeated 5 times in random order. | Movement-onset latency;  Sequence of rotation of body segments;  Effect of target predictability;  Effect of turn angle;  Effect of turn direction. | Three-dimensional CODA motion-analysis system recorded markers on the head, shoulders, pelvis, and feet; Ulmer VNG tracked horizontal eye displacement. | Delayed movement onset with increasing turn angle. Earlier onset when turning to predictable targets. Simultaneous movements of eyes, head, and shoulder for unpredictable targets; for predictable targets, head and shoulder moved before the eyes. For unpredictable 45º and 90º targets, pelvis and feet moved separately, and for 135º they moved simultaneously. For predictable targets at 90º and 135º, pelvis and feet moved simultaneously. |
| Bonnyaud et al., 2016 | Task: Timed “Up and Go” test: stand up, walk 3 meters, go around an object, return, and sit down. Stroke patients turned toward the more affected side; healthy participants, toward the non-dominant side. Comfortable speed, without orthoses or assistive devices. Three repetitions. | Time of the TUG “turn” phase;  Cadence;  Step width;  Step length;  Percentage of single-limb stance;  Percentage of swing phase;  Peak hip flexion/extension;  Peak knee flexion/extension;  Peak ankle dorsiflexion/plantarflexion;  Maximum ankle dorsiflexion in swing. | Motion system with 8 cameras (Motion Analysis Corporation) recorded 34 markers at anatomical landmarks. Three moments were defined for analysis according to the test: “Go”, “Turn” and “Return”. | Longer execution time of the “Turn” phase. Lower cadence, step width and step length, percentage of single-limb stance and of swing. Lower peaks of hip extension, knee flexion, ankle dorsiflexion, and lower maximum dorsiflexion in swing on the more affected side. |
| Liang, Chen, Lee, 2018 | Task: 180º in-place turn at a comfortable pace. One practice and one actual test for each direction (right/left). Initial direction was chosen by the participant. | Turn time;  Number of steps;  Strategy used for the turn;  Balance during the turn. | FUJIFILM F200EXR video camera (Japan) used to capture movements | Longer time (> 3 seconds) and more steps (5 or more) to complete the turn. Adoption of a “stepping” strategy. Greater instability, requiring assistance. |
| Abdollahi et al., 2021 | Position: Standing, looking at a target at shoulder height.  Task: 360º turn at a normal pace. | Number of cycles;  Turn duration;  Mean angular velocity of flexion of the trunk, sacrum, and knee;  ROM of lateral flexion of the trunk and sacrum;  ROM of flexion/extension of the trunk, sacrum and knee. | Four XSens IMU sensors (Enschede, Netherlands) to capture kinematic data, positioned on the legs, sternum and sacrum. | Greater number of cycles, longer turn duration, and greater ROM of lateral flexion of the trunk and sacrum. |
| Abdollahi et al., 2022 | Position: Standing on a floor mark, gaze straight ahead, without looking at the ground. Using a harness attached to a ceiling rail for safety.  Task: 360º in-place turn at a comfortable pace. A virtual target indicated the final position. | Angular velocity of flexion/extension, lateral flexion, and rotation of the sternum and sacrum;  ROM of flexion/extension, lateral flexion, and rotation of the sternum and sacrum;  Angular velocity of knee flexion/extension and rotation;  Knee flexion/extensino and rotation ROM;  Number of cycles;  Total turn duration;  Percentage of stance phase in each cycle (%). | Portable inertial-measurement-unit (IMU) system by Xsens, with four sensors positioned on the sternum, sacrum, and each leg. | Higher angular velocity of flexion/extension and rotation of the sternum and sacrum. Greater ROM of flexion/extension and lateral flexion of the sternum and sacrum. Lower angular velocity of knee flexion/extension and rotation for both the lead and trail legs. Lower knee flexion/extension ROM only at the beginning of the turn. Greater number of cycles, longer total turn duration, and hgiher percentage of stance phase. |
| Soangra et al., 2021 | Position: Standing, facing a target at shoulder height, without looking at the ground.  Task: 360º turn at self-selected pace. The turn should end by repositioning the feet on predefined marks on force plates (1 m x 2 m). Two trials per direction (right/left). | Number of turning cycles; Critical time;  Number of critical single-support phases;  Time of double support with feet apart;  Time of double support with feet together;  Mean time spent in each turning cycle. | Motion-capture system with 10 infrared cameras (Nexus 1.2, Vicon) recorded 26 reflective markers at anatomical landmarks. The task was performed on two force plates (GRAIL, Motek Medical). | Greater number of turning cycles (mean 5.4), longer critical time, greater number of critical single-support phases, longer double-support time, and longer time in each turning cycle. |

GRAIL: Gait Real-time Analysis Interactive lab; IMU: Inertial measurement unit; ROM: Range of movement; TUG: Timed Up and Go test; VNG: videonistagmography.
