# Supplementary material for: Task‐Based Mapping of Compensatory Strategies and Movement Kinematics After Stroke: A Systematic Scoping Review
Source: Physiother Res Int. 2026 Apr 13;31(2):e70215. doi: 10.1002/pri.70215 (PMC13076240; doi:10.1002/pri.70215)
Supplement: Supplementary file 12 — Table S12: Description of the participants' characteristics in each included study for the postural control task. [file PRI-31-e70215-s003.docx]

**Table S12**. Description of the participants’ characteristics in each included study for the postural control task.

| **Author/year** | **Study type** | **N / age (years)** | **Stroke site and/or type** | **Time-based classification** | **Muscle strength** | **Spasticity** | **Assessment tools** |
| --- | --- | --- | --- | --- | --- | --- | --- |
| Goldie et al., 1996 | Cross-sectional observational | N = 12 / 64 (56.5 – 67.5) (Stroke)  N = 12 / 66.5 (58 – 70) (Control) | Type: ischemic (n = 11), hemorrhagic (n = 1). | Subacute | Not reported | Not reported | Motor Assessment Scale: 3.7 ± 2.1  10MWT: 37.7 (18.1 – 49.5)* |
| Turnbull, Charteris, Wall, 1996 | Cross-sectional observational | N = 20 / 57.2 ± 10.65 (Stroke)  N = 20 / 61.5 ± 12.98 (Control) | Not reported | Chronic | Not reported | Not reported | Not reported |
| Kusoffsky, Apel, Hirschfeld, 2001 | Cross-sectional observational | N = 8 / 60.12 ± 7.21 (Stroke)  N = 8 / 59 ± 7.25 (Control) | Type: Hemorrhagic (n = 4), ischemic (n = 4) | Subacute and chronic | Not reported | Not reported | FMA-UE: 63.3 ± 2  FMA-LE: 30.9 ± 1.6  MAS-UL: 18 ± 0  NHPT: 53.4 ± 32 |
| Lamontagne, Paquet, Fung, 2003 | Cross-sectional observational | N = 8 / 65 ± 9 (Stroke)  N = 5 / 67 ± 9 (Control) | Type: Ischemic (n = 8). | Subacute and chronic | Not reported | Not reported | CMSA postural: 5.3 ± 0.5  CMSA leg: 4.8 ± 1.4  CMSA foot: 4 ± 1.2  BBS: 47 ± 7.1 |
| Lin et al., 2007 | Cross-sectional observational | N = 20 / 62.9 ± 10.42 (LCVA)  N = 15 / 61.43 ± 8.63 (RCVA)  N = 15 / 62.13 ± 10.37 (HL)  N = 16 / 61.5 ± 8.9 (HR) | Type: Ischemic (LCVA, n = 10; RCVA, n = 8), hemorrhagic (LCVA, n = 10; RCVA, n = 7). | Chronic | Not reported | MAS LCVA:  LE: 0.1 ± 0.2  UE: 0.3 ± 0.3  MAS RCVA:  LE: 0.1 ± 0.1  UE: 0.3 ± 0.3 | BBS:  LCVA: 42.1 ± 8.23  RCVA: 41 ± 1.7 |
| Genthon et al., 2008 | Cross-sectional observational | N = 41 / 58.8 ± 13.5 (Stroke)  N = 40 / 58.4 ± 11.1 (Control) | Not reported | Subacute | Not reported | Não reportado | Not reported |
| Chern et al., 2010 | Comparative experimental | N = 23 / 69.9 ± 4.2 (Stroke)  N = 13 / 70.5 ± 3.8 (Control) | Not reported | Chronic | Not reported | Not reported | BBS: 41.3 ± 10.3 |
| Gray, Ivanova, Garland, 2012 | Comparative experimental | N = 17 / 56.4 ± 13.5 (Stroke)  N = 17 / 56.1 ± 15.2 (Control) | Not reported | Subacute | Not reported | Not reported | CMSA leg: 5.4 ± 1.2  CMSA foot: 5.4 ± 1.2  BBS: 51.3 ± 5.4  CB&M: 47.1 ± 13.2 |
| Mansfield et al., 2012 | Cross-sectional observational | N = 21 / 57.9 ± 15 (No preference stroke)  N = 19 / 61.9 ± 12.3 (Nonparetic preference stroke)  N = 7 / 60 ± 21.1 (Paretic preference stroke) | Type: Ischemic (No preference stroke, n = 20; Nonparetic preference stroke, n = 12; Paretic preference stroke, n = 6), hemorrhagic (No preference stroke, n = 1; Nonparetic preference stroke, n = 6; Paretic preference stroke, n = 0), transforming to hemorrhagic (No preference stroke, n = 0; Nonparetic preference stroke, n = 1; Paretic preference stroke, n = 1). | Subacute | Not reported | Not reported | NIHSS: 3.1 ± 2.8  CMSA leg: 4.7 ± 1.2  CMSA foot: 4.5 ± 1.2 |
| Mansfield et al., 2013 | Cross-sectional observational | N = 18 / 71.8 ± 9.9 (Asymmetric paretic)  N = 52 / 67.9 ± 13.1 (Asymmetric non paretic)  N = 59 / 67.5 ± 13.4 (Symmetric) | Not reported | Chronic | Not reported | Not reported | CMSA leg: 5.2 ± 1.2  CMSA foot: 4.7 ± 1.5  NIHSS: 2.6 ± 2.6 |
| Honeycutt, Nevisipour, Grabiner, 2016 | Comparative experimental | N = 10 / 61.7 ± 3.4 (Fallers stroke)  N = 7 / 57.7 ± 2.5 (Non-fallers stroke) | Type: Ischemic (Fallers stroke, n = 6; Non-fallers stroke, n = 5), hemorrhagic (Fallers stroke, n = 4; non-fallers stroke, n = 1), unknown (Fallers stroke, n = 0; Non-fallers stroke, n = 1). | Chronic | Not reported | Not reported | BBS: 47.7 ± 2.3  10MWT: 9.69 ± 2.8  5TSTS: 25 ± 4.1 |
| Pilkar, Arzouni, Nolan, 2018 | Cross-sectional observational | N = 10 / 57 ± 13.8 (Stroke)  N = 12 / 56.3 ± 16.5 (Control) | Not reported | Chronic | Not reported | Not reported | BBS: 49.3 ± 5.4 |
| Martinez et al., 2019 | Comparative experimental | N = 20 / 54.7 ± 12.4 (Stroke)  N = 16 / 52.8 ± 16.4 (Control) | Not reported | Chronic | Not reported | Not reported | ABC: 82.2 ± 10.1  TUG: 13.6 ± 3.9 |

10MWT: 10-meter walk test; 5TSTS: Five Times Sit-to-Stand; ABC: Activities-specific Balance Confidence Scale; BBS: Berg Balance Scale; CB&M: Community Balance and Mobility Scale; CMSA: Chedoke-McMaster Stroke Assessment; FMA-LE: Fugl-Meyer Assessment – Lower Extremity; FMA total: Fugl-Meyer Assessment – Total score; HL: healthy controls using the left arm; HR: healthy controls using the right arm; MAS: Motor Assessment Scale; MAS-UL: Motor Assessment Scale – Upper Limb; NHPT: Nine-Hole Peg Test; NIHSS: National Institutes of Health Stroke Scale; TUG: Timed Up and Go test.

*values presented as median (interquartile range)
