# Supplementary material for: Task‐Based Mapping of Compensatory Strategies and Movement Kinematics After Stroke: A Systematic Scoping Review
Source: Physiother Res Int. 2026 Apr 13;31(2):e70215. doi: 10.1002/pri.70215 (PMC13076240; doi:10.1002/pri.70215)
Supplement: Supplementary file 13 — Table S13: Description of the postural control task in each included study. [file PRI-31-e70215-s006.docx]

**Table S13.** Description of the postural control task in each included study.

| **Author/year** | **Task description** | **Kinematic outcomes of interest** | **Movement analysis instrument used** | **Results** |
| --- | --- | --- | --- | --- |
| Goldie et al., 1996 | Position: Standing, with one foot on a force plate and the other maintaining light contact with the floor. Feet were placed in standardized positions: for the lateral direction (feet parallel with 100 mm between them); for the anterior direction, in a step posture.  Task: Displace the hip and shoulders to transfer as much weight as possible to the leg on the force plate and maintain the position for 5 seconds without losing balance. | Percentage of body weight transferred to each leg. | Force plate (Kistler 9281) used to measure vertical reaction force during maximal single-leg weight transfer | Reduced weight transfer in the lateral and anterior directions for both limbs, with lower values on the more affected limb |
| Turnbull, Charteris, Wall, 1996 | Position: First, standing with feet parallel, 8 cm apart, eyes fixed on a point on the wall. Then, with the feet on force plates adjusted in two diagonal configurations: one with the left foot behind and the right in front (diagonal D1) and the other with the positions inverted (diagonal D2).  Task: Assessment of weight transfer in quiet stance, with maximal displacement of weight forward, backward, to the right foot, and to the left foot; and, in D1 and D2, with maximal transfer to the rear foot, front foot, and symmetric distribution. | Center of pressure (COP) position;  COP displacement amplitude;  Stability area. | Chatteex BalanceSystem with 4 pressure plates (forefoot and heel) to measure weight distribution while participants stood. | COP shifted toward the less affected side. Difficulty transferring weight backward. Deficit in diagonals D1 and D2 for both limbs. Forward transfer: adequate in the anteroposterior axis, deficient in the mediolateral axis. Lower COP displacement amplitude in all directions. Regarding stability area: left-hemiplegia group with anterolateral shift and low backward transfer; right-hemiplegia group with irregular area, with deficits especially to the right and posterior. |
| Kusoffsky, Apel, Hirschfeld, 2001 | Position: Standing, distance to the table equal to arm length, starting with both arms alongside the body.  Task: Reach, grasp, and move a milk carton (0.5 L) fROM one table (72 cm height) to a lateral table (65 cm). Five repetitions for each arm. | Symmetry of weight distribution during initial quiet stance;  Changes in weight distribution during task execution. | ELITE system with 2 cameras, using only wrist and object markers for analysis. | Asymmetric weight distribution favoring the less affected side. In the initial phase, peak loading amplitude was lower with the less affected hand. During task execution, weight was transferred to the side contralateral to the active hand. With use of the more affected hand, a greater increase in load occurred on the less affected side. |
| Lamontagne, Paquet, Fung, 2003 | Position: Standing, with a light arrow positioned 3 meters away indicating the direction of head movements.  Task: Perform rapid head movements upward, downward, to the right, and to the left according to the appearance of the light arrow, maintaining the position for 3 seconds. | Maximum head-movement velocity;  Head displacement and velocity relative to space and to the trunk;  COM and COP displacement in AP and ML directions;  Differences between COP and COM displacement;  Root mean square (RMS) values for COM and COP displacement and velocity. | 3D motion-analysis system (Vicon512) with 6 cameras and 23 reflective markers, plus 2 triaxial force plates (AMTI OR6-7). | Lower maximum head velocity in all directions. Greater COP displacement, mainly in the ML direction. Larger difference between COP and COM. Higher RMS of displacement and velocity. |
| Lin et al., 2007 | Position: Hand placed on a switch in the midsagittal plane, with the table and switch adjusted to 100% of leg length.  Task: Perform forward reaching under two conditions: object present, holding a plastic cup and moving it along a 10-cm track aligned with the switch; and object absent, extending the arm without holding anything. The stroke group used the less affected arm. | Anterior COP displacement;  Mediolateral COP displacement;  Mean COP velocity. | VICON 370 3D motion-analysis system with 6 cameras and infrared markers, plus 2 AMTI OR6-6-1000 force plates to measure orthogonal forces and compute COP. | Lower anterior COP displacement; increase with object only in the RCVA group. Greater ML COP displacement. |
| Genthon et al., 2008 | Position: Standing, barefoot, each foot on one of two side-by-side force plates. Heels separated (9 cm) and toes turned outward (30°), arms relaxed alongside the body, gaze fixed on a frontal target.  Task: Maintain postural stability for 4 trials of 32 seconds with rest intervals (1 and 3 minutes). The stroke group adopted a spontaneous posture and the control group an asymmetric body-weight distribution. | Mean resultant COP position (COPres) and under each foot along ML and AP axes;  Mean amplitude of COPres trajectories along ML and AP axes and under each foot;  Mean frequency of COPres trajectories along ML and AP axes and under each foot;  Elongation ratio. | Rectangular force plates PF02 (Equi+, France) with 4 vertical load cells each to capture vertical reaction force. | More lateralized COP, mainly on the more affected (less loaded) foot. Greater mean COP amplitude in ML and AP axes. Higher mean trajectory frequency, especially in the AP axis, with no difference between feet. Reduced elongation ratio on the more affected foot. |
| Chern et al., 2010 | Position: Standing, feet shoulder-width apart, gaze forward.  Task: Trunk flexion and arm extension to touch a target on the floor, then return to upright. Targets were positioned in 6 different locations, varying in distance (10% and 30% of body height) and direction (center, more affected/non-dominant side, and less affected/dominant side). | Total COP excursion;  Maximal COP displacement in AP and ML directions;  Mean COP velocity;  Load ratio between lower limbs;  Posturography. | Pressure platform (RSscan International) that recorded COP trajectory and weight distribution between lower limbs. | Greater total COP excursion, except with a distant target on the less affected side. Greater maximal COP displacement in the AP direction, influenced by target position. Lower displacement in the ML direction, except with a target on the less affected side. Lower interlimb load ratio, except with a target on the less affected side. Greater COP sway directed toward the less affected side, except with a distant target on the more affected side. More irregular COP trajectory, mainly during return to upright. |
| Gray, Ivanova, Garland, 2012 | Position: Standing, feet on separate force plates, equipped with a safety belt that did not provide weight support.  Task: Rapid squat, flexing the knees to ~30°, hold for 1 second, then return slowly to the starting position. Rest 6 seconds before the next squat. | Knee acceleration;  COP displacement;  Vertical COM acceleration;  Maximum vertical velocity;  Maximum vertical displacement. | T45-10 acceleROMeter fixed near the knees to detect onset, acceleration, and deceleration of the squat, and AMTI OR6-1000 force plates to measure reaction forces and moments. | Lower knee acceleration. Decoupling between COP displacement and knee acceleration/deceleration. Lower COM vertical acceleration and vertical velocity. |
| Mansfield et al., 2012 | Position: Standing on two force plates, leaning forward with approximately 10% of body weight supported by a cable attached to the wall. A safety harness attached to an overhead frame was used, with a therapist nearby for support.  Task: After the cable was unexpectedly released, an anterior fall was initiated and participants were to take a compensatory step under two conditions: one with free choice of the limb to initiate the step, and another with the preferred limb blocked. | Limb used to initiate the compensatory step;  Frequency of step initiation with the paretic versus non-paretic limb;  Clear limb preference;  Load in quiet stance;  Peak load on the paretic limb;  Pre-perturbation load on the paretic limb;  Inadequate responses;  External assistance required. | Force plates to measure weight distribution between lower limbs and video to record compensatory stepping responses. | More than half of steps were initiated with the less affected limb. More than half of mean load on the less affected limb. Increased load on the more affected limb before perturbation in those with preference for the less affected limb. In 21% of trials, there was an inadequate response to blocking the preferred limb. In 21.7% of trials with a step, assistance was required to prevent a fall, more common in patients with poorer motor recovery. |
| Mansfield et al., 2013 | Position: Standing on two side-by-side force plates, feet oriented at 14° and 0.17 m between heels, looking at a mark on the wall at eye height.  Task: Maintain quiet standing for 30 seconds under two conditions: eyes open and eyes closed. | Weight-bearing asymmetry;  Magnitude of asymmetry;  COP displacement. | Two side-by-side force plates to measure reaction forces and moments during quiet standing. | 12.2% of participants showed paretic asymmetry (PA; more weight on the more affected side). 47.6% showed non-paretic asymmetry (NPA; more weight on the less affected side). 40.1% were classified as symmetric. The NPA group had greater absolute asymmetry than the PA group. Greater COP displacement in the ML direction in both PA and NPA groups. Lower contribution of the more loaded limb to balance control in the AP direction in the PA group. |
| Honeycutt, Nevisipour, Grabiner, 2016 | Position: Standing on a split-belt treadmill, using a ceiling-mounted safety harness that allowed a natural fall, preventing hand or knee contact with the treadmill in case of failure to recover.  Task: Divided into three phases: pre-test, training, and post-test. In pre- and post-test, participants received 6 perturbations (three forward and three backward) with increasing difficulty. Backward perturbations were classified into three levels (small, medium, and large), whereas forward perturbations had insufficient magnitude to cause a fall, aiming to reduce anticipation of perturbation direction. During training, 15 backward perturbations of moderate magnitude were delivered. Participant responses were classified as “fall” or “recovery.” | Reaction time;  Step duration;  Step length;  Trunk flexion;  Knee flexion;  Dx (distance between the vertical projection of the COM and the edge of the base of support);  Trunk-flexion velocity;  Stance-leg flexion velocity;  Knee-flexion velocity;  Dx velocity. | Motion-capture system with 8 cameras (Motion Analysis Co.) recording 22 passive markers on upper limbs, lower limbs, and trunk. | Shorter step length in trials resulting in “fall.” Greater knee flexion and higher end-of-step knee-flexion velocity in trials resulting in “recovery.” More negative Dx at the beginning and end of the step and higher end-of-step trunk-flexion velocity in “fall.” |
| Pilkar, Arzouni, Nolan, 2018 | Position: Standing, unsupported, on a flat surface, eyes open, adopting a natural standing posture, looking forward.  Task: Maintain quiet standing for 120 seconds, without holding any object, under research-staff supervision to ensure safety. | COP displacement;  Mean and peak COP velocity;  Stability indices;  Inter-limb symmetry ratios. | Plantar-pressure measurement system (pedar®-x) with a waist-worn portable device and two sensor insoles inserted in the shoes. | Greater COP displacement and peak velocity on the less affected side in the AP direction. Higher mean COP velocity on the less affected side in AP and ML directions. Higher PPI, PI, and VI indices on the less affected side. Significantly lower inter-limb symmetry ratios. |
| Martinez et al., 2019 | Position: Standing, feet shoulder-width apart, each foot on a separate force plate, using a belt attached to the waist connected to a cable adjusted to umbilical height.  Task: Receive an anterior perturbation (force equivalent to 10% of body weight) triggered upon reaching the desired weight distribution. Three weight-distribution conditions were tested: symmetric posture (50% on each foot), preferred asymmetric posture (70% on the preferred stance leg), and non-preferred asymmetric posture (70% on the non-preferred stance leg). | Step initiation;  Step length;  Step width;  Step height;  Step duration;  Number of steps;  Body velocity at heel contact. | Two AMTI OR6-6 force plates and an 8-camera motion system (QTM-Qualisys) recorded data with reflective markers on points of the legs and trunk (malleoli, calcaneus, metatarsals, condyles, and acROMia). | Earlier step initiation with the less affected leg, mainly in non-preferred asymmetric support. Shorter step length with the more affected leg. Longer step duration with the more affected leg. Greater incidence of steps to recover balance, mainly with the more affected leg. Lower body velocity at heel contact when using the more affected leg. |

AMTI: Advanced Mechanical Technology, Inc.; AP: Anteroposterior; CM: Center of mass; CP: Center of pressure; ML: medio-lateral; NPA: nonparetic asymmetry; PA: paretic asymmetry; PI: Pressure index; PPI: Peak pressure index; RCVA: Right cerebrovascular accident; RMS: Root mean square; VI: Velocity index; QTM: Qualisys Track Manager.
