# Supplementary material for: Task‐Based Mapping of Compensatory Strategies and Movement Kinematics After Stroke: A Systematic Scoping Review
Source: Physiother Res Int. 2026 Apr 13;31(2):e70215. doi: 10.1002/pri.70215 (PMC13076240; doi:10.1002/pri.70215)
Supplement: Supplementary file 14 — Table S14: Description of the participants' characteristics in each included study for the reaching, grasping and manipulation task. [file PRI-31-e70215-s013.docx]

**Table S14.** Description of the participants’ characteristics in each included study for the reaching, grasping and manipulation task.

| **Author/year** | **Study type** | **N / age (years)** | **Stroke site and/or type** | **Time-based classification** | **Muscle strength** | **Spasticity** | **Assessment tools** |
| --- | --- | --- | --- | --- | --- | --- | --- |
| Levin, 1996 | Comparative experimental | N = 10 / 48.5 ± 9.28 (Stroke)  N = 6 / Age not reported (Control) | Site: Internal capsule (n = 5), frontoparietal cortex (n = 4), parietal cortex (n = 1) | Chronic | Not reported | Not reported | FMA-UE: 48.8 ± 16.5 |
| Roby-Brami et al., 1997 | Comparative experimental | N = 17 / 52.82 ± 10.95 (Stroke)  N = 6 / 51.8 (41 – 58) (Control) | Type: Hemorrhagic (n = 4);  Ischemic [n = 11 (MCA n = 2; ACA n = 1)];  Sinus thROMbosis (n = 2) | Subacute and chronic | Not reported | Not reported | Not reported |
| Archambault et al., 1999 | Comparative experimental | N = 8 / 50 ± 15.16 (Stroke)  N = 6 / 46 ± 16 (Control) | Type: Hemorrhagic (n = 7), embolic (n = 1)  Site: Temportal lobe (n = 1), parietal lobe (n = 2), temporoparietal lobe (n = 1), internal capsule (n = 3), basal ganglia (n = 3), thalamus (n = 1), MCA (n = 3) | Chronic | Not reported | CSI: 7.5 ± 1.8 | FMA-UE: 50.2 ± 14.2 |
| Cirstea, Levin, 2000 | Comparative experimental | N = 9 / 54.8 ± 14.58 (Stroke)  N = 9 / 43 ± 18 (Control) | Type: Ischemic (n = 6), hemorrhagic (n = 3)  Site: Frontotemporoparietal lobe (n = 2), frontoparietal lobe (n = 1), parietal lobe (n = 2), temporal lobe (n = 1), subcortical (n = 2), internal capsule (n = 1), thalamus (n = 1), MCA (n = 2) | Subacute and chronic | Not reported | MAS: 1.4 ± 1 | FMA-UE: 40.7 ± 17.6 |
| Michaelsen et al., 2001 | Comparative experimental | N = 11 / 54.8 ± 13.9 (Stroke)  N = 11 / 55 ± 13.7 (Control) | Type: Ischemic (n = 8), hemorrhagic (n = 3)  Site: Internal carotid artery, parietal lobe and subcortical (n = 1), temporal lobe (n = 1), frontoparietal lobe (n = 1), thalamus (n = 1), posterior internal capsule (n = 2), MCA (n = 5). | Chronic | Not reported | CSI: 7.9 ± 2.4 | FMA-UE: 47.5 ± 14.6  BBS: 50.8 ± 4.6 |
| Kusoffsky, Apel, Hirschfeld, 2001 | Cross-sectional observational | N = 8 / 60.12 ± 7.21 (Stroke)  N = 8 / 59 ± 7.25 (Control) | Type: Hemorrhagic (n = 4), ischemic (n = 4) | Subacute and chronic | Not reported | Not reported | FMA-UE: 63.3 ± 2  FMA-LE: 30.9 ± 1.6  MAS-UL: 18 ± 0  NHPT: 53.4 ± 32 |
| Kamper et al., 2002 | Cross-sectional observational | N = 16 / 30 – 85 anos (Stroke)  N = 4 / 24 – 39 anos (Control) | Not reported | Chronic | Not reported | Not reported | Not reported |
| Levin et al., 2002 | Comparative experimental | N = 11 / 54.8 ± 13.9 (Stroke)  N = 11 / 55.0 ± 13.7 (Control) | Tipo: Isquêmico (n = 8), hemorrágico (n = 3)  Site: temporal lobe (n = 1), frontoparietal lobe (n = 1), parietal lobe (n = 1), thalamus (n = 1), posterior internal capsule (n = 2), MCA (n = 5), internal carotid artery (n = 1). | Chronic | Not reported | CSI: 8 ± 2 | FMA-UE: 47.5 ± 14.6  BBS: 50.8 ± 4.6 |
| Cirstea et al., 2003 | Comparative experimental | N = 18 / 54.22 ± 16.57 (Stroke)  N = 10 / 43 ± 18 (Control) | Site: MCA (n = 7), frontoparietal lobe (n = 4), parietal lobe (n = 2), insula (n = 1), temporoparietal lobe (n = 1), temporal lobe (n = 1), subcortical (n = 1), corona radiata (n = 1), internal carotid artery (n = 1). | Chronic | Not reported | CSI: 7.8 ± 2.1 | FMA-UE: 41.6 ± 19.8 |
| Roby-brami et al., 2003 | Comparative experimental | N = 15 / 55.66 ± 10.12 (Stroke)  N = 7 / 35.8 (22 – 53) (Control) | Site: MCA (n = 14), basilar artery (n = 1).  Type: Ischemic (n = 15). | Subacute and chronic | Not reported | Not reported | Not reported |
| Roby-brami et al., 2003 | Comparative experimental | N = 8 / 55.75 ± 9.54 (Stroke)  N = 7 / 35.8 (22 – 53) (Control) | Site: MCA (n = 7), basilar artery (n = 1).  Type: Ischemic (n = 8). | Subacute and chronic | Grip force: 54.7 ± 15.1 | Not reported | FAT: 4.3 ± 0.7  NHPT: 72.5 ± 48.7  BBT: 34 ± 19.5 |
| Reisman, Scholz, 2003 | Comparative experimental | N = 8 / 60.6 ± 4.7 (Stroke)  N = 8 / 60.7 ± 4.6 (Control) | Type: Ischemic (n = 7); hemorrhagic (n = 1).  Site: Insular region e subinsular (n = 1), pons (n = 2), putamen (n = 2), thalamus (n = 1), internal capsule (n = 1), left side of the cortex (n = 1). | Chronic | Not reported | Not reported | FMA-UE: 44.7 ± 8 |
| Michaelsen et al., 2004 | Comparative experimental | N = 19 / 52.36 ± 19.22 (Stroke)  N = 7 / 53 ± 24 (Control) | Type: Ischemic (n = 7), hemorrhagic (n = 3)  Site: MCA (n = 5), arteriovenous malformation (n = 2), parietal lobe (n = 3), temporal lobe (n = 1), temporoparietal lobe (n = 1), thalamus (n =1), internal capsule (n = 1). | Chronic | Wrist extension (kgF): 4 ± 3 | Not reported | FMA-UE: 41.2 ± 12.2  BBT: 17 ± 17 |
| Zackowski et al., 2004 | Comparative experimental | N = 18 / 55 ± 2.4 (Stroke)  N = 18 / 54.6 ± 2.4 (Control) | Type: Ischemic (n = 14), hemorrhagic (n = 4).  Site: Frontal lobe (n = 9), temporal lobe (n = 5), parietal lobe (n = 7), basal ganglia (n = 3), external capsule (n = 1), putamen (n = 2), caudate nucleus (n = 1), pons (n = 3), midbrain (n = 1). | Subacute and chronic | Upper limb dynamometry (lbs): 18.6 ± 13.0 | Upper limb MAS: 1.1 ± 0.9 | Not reported |
| McCrea, Eng, Hodgson, 2005 | Comparative experimental | N = 22 / 60.9 ± 6.1 (Stroke)  N = 10 / 61 ± 9 (Control) | Type: Ischemic (n = 12); hemorrhagic (n = 8).  Site: Cortical (n = 7); subcortical (n = 13). | Chronic | Not reported | Not reported | FMA-UE: 38.2 ± 19  Motor Assessment Scale: 1.3 ± 1.1 |
| Rose, Winstein, 2005 | Comparative experimental | N = 30 / 63 (27 – 87) (Stroke)  N = 30 / 67 (49 – 86) (Control) | Not reported | Chronic | Grip (%): 65 (27 – 100);  Lateral pinch (%): 71 (27 – 100);  Palmar pinch (%): 63 (24 – 100) | Not reported | FMA-UE: 60 (43 – 66) |
| Wenzelburger et al., 2005 | Cross-sectional observational | N = 18 / 60.9 ± 10.7 (Stroke)  N = 18 / 62 anos (Control) | Type: Ischemic (n = 18)  Site: Internal capsule (n = 18) | Chronic | Wrist extensors MRC: 4.4 ± 0.8 | Wrist extensors MAS: 1.4 ± 1.5 | RMA: 10.6 ± 2.9  FIM: 84.6 ± 12 |
| Kilbreath et al., 2006 | Cross-sectional observational | N = 13 / 67.9 ± 8.3 (Stroke)  N = 13 / 69.6 ± 9.9 (Control) | Not reported | Chronic | Not reported | Ashworth scale: 0.9 ± 0.8 | FAT: 4.7 ± 0.8 |
| Messier et al., 2006 | Cross-sectional observational | N = 15 / 69.4 ± 12 (Stroke)  N = 13 / 67.8 ± 7.5 (Control) | Type: Ischemic (n = 14); hemorrhagic (n = 1)  Site: MCA (n = 11), vertebrobasilar (n = 1), lacunar (n = 2) | Chronic | Not reported | Not reported | FMA-UE: 43 - 63 |
| Nowak et al., 2007 | Cross-sectional observational | N = 16 / 54.5 ± 16.6 (Stroke)  N = 8 / 56 ± 17 (Control) | Type: Ischemic (n = 16)  Site: MCA (n = 16), ganglia nuclei (n = 14), internal capsule (n = 8), corona radiata (n = 9). | Subacute and chronic | Wrist extensors MRC:4.5 ± 0.6 | Not reported | NIHSS: 3.6 ± 2.3  mRS: 1.6 ± 0.7  ARAT: 41.5 ± 13.6 |
| Van vliet, Sheridan, 2007 | Comparative experimental | N = 12 / 66.9 anos (Stroke)  N = 12 / 64.8 anos (Control) | Site: MCA (n = 12). | Subacute and chronic | Not reported | Elbow MAS: 0.5 ± 0.6  Wrist MAS: 0.4 ± 0.9  Finger MAS: 0.3 ± 0.8 | RMA: 9.1 ± 2.0 |
| Michaelsen, Magdalon, Levin, 2009 | Cross-sectional observational | N = 12 / 66 ± 14 (Stroke)  N = 7 / 64 ± 17 (Control) | Site:MCA (n = 4), parietal lobe (n = 2), occipitoparietal lobe (n = 1), ganglia nuclei (n = 2), thalamus (n = 2), corona radiata (n = 1), internal capsule (n = 1), basilar artery (n = 1). | Chronic | Grip strength ratio: 0.3 ± 0.1  Wrist extensor strength ratio: 0.7 ± 0.1 | Not reported | FMA-UE: 51 ± 7  BBT: 25.2 ± 12.7 |
| Sangole, Levin, 2009 | Cross-sectional observational | N = 10 / 65 ± 9 (Stroke)  N = 8 / 55 ± 10 (Control) | Not reported | Chronic | Not reported | Not reported | CMSA arm: 5.3 ± 1.3  CMSA hand: 4.8 ± 1.5 |
| Raghavan et al., 2010 | Comparative experimental | N = 8 / 56.3 ± 18.2 (Stroke)  N = 8 / Age not reported (Control) | Type: Ischemic (n = 7); hemorrhagic (n = 1)  Site: Internal capsule (n =7); nuclei ganglia (n = 2); thalamus (n = 1). | Chronic | Not reported | Shoulder MAS: 0.2 ± 0.4  Elbow MAS: 1 ± 0.8  Wrist MAS: 0.6 ± 0.5 | FMA-UE: 47.3 ± 5.5.  WMFT: 5.1 ± 1.3 |
| Alt Murphy; Willén; Sunnerhagen, 2011 | Cross-sectional observational | N = 19 / 61 ± 11.1 (Stroke)  N = 19 / 57.3 (41-78) (Control) | Type: Ischemic (n = 14); hemorrhagic (n = 5) | Chronic | Not reported | Not reported | FMA-UE: 53.4 ± 8.7 |
| Robertson, Roby-Brami, 2011 | Comparative experimental | N = 16 / 52.4 ± 15.5 (Stroke)  N = 10 / 41 (25 – 69) (Control) | Site: MCA (n = 8), ACA (n = 1), pontine artery (n = 1), coroidal artery (n = 1), thalamus (n = 2), parieto-occipital lobe (n = 1), frontoparietal lobe (n = 1). | Subacute and chronic | Not reported | Not reported | BBT: 27.7 ± 20.1  ARAT: 38.8 ± 15.8  Barthel index: 83.4 ± 25.7 |
| DeJong, Lang, 2012 | Comparative experimental | N = 16 / 59 ± 11 (Stroke)  N = 11 / Age not reported (> 30 years) (Control) | Type: Ischemic (n = 14); hemorrhagic (n = 2) | Subacute and chronic | Not reported | Elbow flexors MAS: 0.6 ± 0.6 | ARAT: 41 ± 9  SIS: 55.5 ± 19.7 |
| Robertson, Roche, Roby-Brami, 2012 | Comparative experimental | N = 18 / 52 ± 13.8 (Stroke)  N = 9 / 51 (29 – 71 years) (Control) | Type: Ischemic (n = 12); hemorrhagic (n = 6)  Site: MCA (n = 12); ACA (n = 2); anterior choroidal artery (n = 1); temporal lobe (n = 1); supplementar motor area (n = 1); parieto-frontal lobe (n = 1); capsulothalamic region (n = 1); capsulolenticular region (n = 1). | Chronic | MRC:  Shoulder flexion: 3.8 ± 0.4  Shoulder abduction: 3.8 ± 0.3  Shoulder internal rotation: 4 ± 0  Shoulder external rotation: 3.8 ± 0.4  Elbow flexion: 3.8 ± 0.6  Elbow extension: 4 ± 0 | MAS:  Elbow flexors: 1.1 ± 1.3  Shoulder adductors: 0.5 ± 0.9  Shoulder internal rotators: 0.6 ± 0.9 | FAT: 3.2 ± 1.6  Barthel index: 98.4 ± 2.8 |
| Schaefer et al., 2012 | Comparative experimental | N = 16 / 58 ± 11 (Stroke)  N = 12 / 53 ± 16 (Control) | Type: Ischemic (n = 14); hemorrhagic (n = 2) | Subacute and chronic | Grip strength: 70.3 ± 25.1  Pinch strength: 67.7 ± 22 | Elbow flexors MAS: 0.7 ± 1.1 | ARAT: 40.2 ± 9.3  SIS: 49.7 ± 21.4 |
| Van Kordelaar, van Wegen, Kwakkel, 2012 | Cross-sectional observational | N = 46 / 60.3 ± 12.59 (Stroke)  N = 12 / 52.75 ± 5.88 (Control) | Type: Ischemic (n = 45); hemorrhagic (n = 1) | Subacute and chronic | Not reported | Not reported | NIHSS: 1 (0 – 4)  FMA-UE: 63 (51 – 65)  ARAT: 45 (38 – 57) |
| Merdler et al., 2013 | Comparative experimental | N = 16 / 65.2 ± 9.8 (Stroke)  N = 8 / 58.6 ± 7 (Control) | Type: Ischemic (n = 10); hemorrhagic (n = 4)  Site: MCA (n = 3), ganglia nuclei (n = 5), internal capsule (n = 2), thalamus (n = 2), parietal lobe (n = 1), pons (n = 1). | Subacute and chronic | Not reported | Not reported | FMA-UE: 42.9 ± 10.7  BBT: 59.5 ± 32.3 |
| Aprile et al., 2014 | Cross-sectional observational | N = 6 / 78 ± 7.4 (Stroke)  N = 6 / 64.5 (52 – 74 years) (Control) | Type: Ischemic (n = 4); hemorrhagic (n = 2). | Subacute and chronic | Not reported | Not reported | FMA-UE: 43.5 ± 6.9  Barthel index: 49.6 ± 9.9 |
| Shaikh et al., 2014 | Comparative experimental | N = 11 / 66.1 ± 15.5 (Stroke)  N = 11 / 51.6 ± 14.5 (Control) | Site: MCA (n = 8), internal capsule (n = 2), pontomesencephalic junction (n = 1), corona radiata (n = 1), frontoparietal lobe (n = 1). | Subacute and chronic | Hand-held dynamometry:  Shoulder flexors: 66.3 ± 14.2  Elbow extensors: 72 ± 16.3  Wrist extensors: 76 ± 14.3 | CSI: 7.5 ± 1.2 | FMA-UE: 52.5 ± 10.9  BBS: 51.9 ± 4.7 |
| Stewart, Gordon, Winstein, 2014 | Comparative experimental | N = 14 / 59.1 ± 10.7 (Stroke)  N = 6 / 63.8 ± 14.4 (Control) | Site: ganglia nuclei (n = 5); internal capsule (n = 7); thalamus (n = 1); insula (n = 1); temporal lobe (n = 3); MCA (n =1); frontal lobe (n = 1); parietal lobe (n = 1). | Chronic | Not reported | Not reported | FMA-UE: 52.7 ± 7.9  SIS: 62.1 ± 22.7  ARAT: 44.5 ± 10.1 |
| Levin et al., 2016 | Comparative experimental | N = 16 / 65.2 ± 9.8 (Stroke)  N = 8 / 58.6 ± 7 (Control) | Type: Ischemic (n = 10), hemorrhagic (n = 4), not reported (n = 2)  Site: MCA (n = 3), ganglia nuclei (n = 5), internal capsule (n = 2), parietal lobe (n = 1), thalamus (n = 2) | Chronic | Not reported | Not reported | FMA-UE: 44.2 ± 10.6 |
| Alvarez et al., 2017 | Comparative experimental | N = 38 / 54 ± 15 (Stroke)  N = 10 / 32 ± 6 (Control) | Not reported | Subacute and chronic | Not reported | Not reported | FMA-UE: 38.5 (14)*  ARAT: 25 (17)* |
| Ma et al., 2017 | Comparative experimental | N = 18 / 52.2 ± 11.7 (Stroke)  N = 18 / 52.1 ± 11.9 (Control) | Type: Ischemic (n = 9), hemorrhagic (n = 9).  Site: Cerebral cortex (n = 6), ganglia nuclei (n = 5), pons (n = 2), thalamus (n = 4). | Chronic | Not reported | Not reported | FMA-UE: 48.4 ± 7 |
| Valdes, Glegg, Van der Loos, 2017 | Comparative experimental | N = 10 / 65.4 ± 8.9 (Stroke)  N = 10 / 65.2 ± 8.68 (Control) | Type: Ischemic (n = 6), hemorrhagic (n = 4).  Site: MCA (n = 2), PCA (n = 1), internal capsule (n = 2), external capsule (n = 2), ganglia nuclei (n = 3), pontine artery (n = 1), corona radiata (n = 1), sylvian fissure (n = 1), frontal lobe (n = 1). | Chronic | Not reported | MAS:  Biceps: 0.7 ± 0.9  Triceps: 0.7 ± 0.9  Wrist flexors: 0.7 ± 0.9  Wrist extensors: 0.8 ± 1.0 | FMA-UE: 51.2 ± 12.6 |
| Tomita, Mullick, Levin, 2018 | Comparative experimental | N = 10 / 64.5 ± 7.1 (Mild stroke)  N = 9 / 59.4 ± 9.1 (Moderate stroke)  N = 12 / 65 ± 10 (Control) | Type: Ischemic (n = 10, mild stroke; n = 6, moderate stroke), hemorrhagic (n = 3, moderate stroke). | Chronic | Not reported | CSI: 7 ± 3 | FMA-UE: 48.8 ± 12.2  FMA-LE: 24.2 ± 2.4  MiniBESTest: 13.4 ± 5.1 |
| Thrane et al., 2019 | Cross-sectional observational | N = 56 / 65.4 ± 13.2 (High FMA-UE = 60 - 66)  N = 24 / 68.2 ± 11.8 (Submaximal FMA-UE = 60-65)  N = 21 / (Maximal FMA-UE = 66)  N = 30 / 58.3 ± 12.8 (Control) | Type: high FMA-UE = 60 - 66  Ischemic: n = 44;  Hemorrágico: n = 12  Submaximal FMA-UE = 60-65  Ischemic: n = 19  Hemorrhagic: n = 5  Maximal FMA-UE = 66  Ischemic: n =16  Hemorrhagic: n = 5 | Subacute and chronic | Not reported | Not reported | FMA-UE  high FMA-UE = 60 – 66: 62 (61 – 64)  Submaximal FMA-UE = 60-65: 64 (62 – 65)  Maximal FMA-UE = 66: 66  Admission NIHSS  high FMA-UE = 60 – 66: 4 (3 – 6)  Submaximal FMA-UE = 60-65: 4 (3 – 6)  Maximal FMA-UE = 66: 3 (2 – 6) |
| Feingold-Polak et al., 2021 | Comparative experimental | N = 30 / 70.3 ± 9.3 (Stroke)  N = 16 / 69.1 ± 11.5 (Control) | Site: MCA (n = 23), PCA (n = 3), ACA (n = 2), cerebellum (n = 1), pons (n = 1). | Subacute | Not reported | Not reported | Not reported |
| Padilla-Magaña et al., 2022 | Cross-sectional observational | N = 7 / 62 ± 10.3 (Right stroke)  N = 5 / 69.6 ± 5.3 (Left stroke)  N = 25 / 40.2 ± 18.1 (Control) | Type: Ischemic (n = 10), hemorrhagic (n = 2) | Chronic | Not reported | Not reported | ARAT  Right stroke: 39.2 ± 14.3  Left stroke: 45.4 ± 13.7 |
| Choi et al., 2023 | Cross-sectional observational | N = 16 / 67.44 ± 12.36 (Mild stroke)  N = 15 / 61.87 ± 14.28 (Moderate stroke)  N = 15 / 57.73 ± 13.05 (Severe stroke)  N = 20 / 31.5 ± 5.2 (Control) | Type: mild stroke  Ischemic: n = 15  Hemorrhagic: n = 1  Moderate stroke  Ischemic: n = 13  Hemorrhagic: n = 2  Severe stroke  Ischemic: n = 12  Hemorrhagic: n = 3 | Subacute | Not reported | Not reported | FMA-UE  Mild: 61.9 ± 3  Moderate: 34.1 ± 5.9  Severe: 22 ± 3  WMFT  Mild: 67 ± 9.8  Moderate: 31.1 ± 14.9  Severe: 19.8 ± 3.2  Motricity index  Mild: 77.3 ± 7.7  Moderate: 59.2 ± 12  Severe: 41.9 ± 11  TCT  Mild: 99.1 ± 3.2  Moderate: 92.2 ± 17.3  Severe: 90.6 ± 20.6 |
| Ota et al., 2023 | Comparative experimental | N = 10 / 26.5 ± 4.9 (Stroke)  N = 20 / 70.7 ± 13.8 (Control) | Type: Ischemic (n = 6); hemorrhagic (n = 4) | Subacute and chronic | Not reported | Not reported | FMA-UE: 52.1 ± 10.7 |

ACA: anterior cerebral artery; ARAT: Action Research Arm Test; BBT: Box and Bock test; BBS: Berg Balance Scale; CMSA: Chedoke-McMaster Stroke Assessment; FAT: Frenchay Arm Test; FIM: Functional Independence Measure; FMA-LE: Fugle-Meyer Assessment – Lower Extremity; FMA-UE: Fugl-Meyer – upper limb section; MAS: Motor Assessment Scale; MAS-UL: Motor Assessment Scale – Upper Limb; MCA: middle cerebral artery; MiniBESTest: Mini Balance Evaluation Systems Test; mRS: Modified Rankin Scale; NHPT: Nine-Hole Peg Test; NIHSS: National Institutes of Health Stroke Scale; PCA: posterior cerebral artery; RMA: Rivermead Motor Assessment; SIS: Stroke Impairment Scale; TCT: Trunk Control Test; WMFT: Wolf Motor Function Test.

*values presented as mean (interquartil range)
