# Supplementary material for: Task‐Based Mapping of Compensatory Strategies and Movement Kinematics After Stroke: A Systematic Scoping Review
Source: Physiother Res Int. 2026 Apr 13;31(2):e70215. doi: 10.1002/pri.70215 (PMC13076240; doi:10.1002/pri.70215)
Supplement: Supplementary file 15 — Table S15: Description of the reaching, grasping and manipulation tasks in each included study. [file PRI-31-e70215-s008.docx]

**Table S15.** Description of the reaching, grasping and manipulation task in each included study.

| **Author/year** | **Task description** | **Kinematic outcomes of interest** | **Movement analysis instrument used** | **Results** |
| --- | --- | --- | --- | --- |
| Levin, 1996 | Position: Seated, shoulder adducted, elbow flexed 45°, forearm pronated on a table in front. Trunk motion limited; wrist immobilized in neutral; fingers lightly flexed, maintained by a splint.  Task: Perform reaching movements to 4 computer-cued targets: near target, 200 mm from the start position; far target, 400 mm from the start position; contralateral and ipsilateral targets, both 200 mm lateral to the near target. Ten back-and-forth movements per target. | Movement time, distance, and trajectory (curvature and direction);  Interjoint coordination (shoulder and elbow). | Optotrak motion-analysis system using infrared light–emitting diodes placed on the shoulder, arm, and hand. | Longer movement time in the more affected limb. Reduced joint excursion and trajectory in the more affected limb, especially for contralateral and farther targets. Poorer interjoint coordination between shoulder and elbow in the more affected limb. |
| Roby-Brami et al., 1997 | Position: Seated facing the midline of a board (15 cm from the abdomen), hand resting 10 cm lateral to the midline and 12 cm behind the nearest target.  Task: Reach and grasp a cone, always starting from the same fixed position, and bring it to a specified final position. Order (relative to the moving arm): mid-sagittal, near-sagittal, far-sagittal, far-external, far-internal, near-external, and near-internal. Five repetitions per position. | Hand and trunk sensor trajectories;  Movement duration and peak velocity;  Trunk movement;  Hand orientation at movement end;  Hand height. | Fastrack® Polhemus system using transmitter-generated electROMagnetic fields to determine position and orientation of two remote sensors at 6.8 Hz, positioned on the dorsum of the hand and the acROMion. | Slower and more irregular hand sensor trajectory. Multiple smaller velocity peaks. Longer movement duration. Greater and more variable trunk movement. Trunk movement occurring before the hand and reliance on trunk to reach near targets. Frontal hand orientation. Variation in hand height at grasp. |
| Archambault et al., 1999 | Position: Seated 80 cm from the floor, at a 180 × 120 cm table; shoulder at 70° flexion and abduction, elbow at 60–90° flexion; initial target 20 cm from the sternum.  Task: Reach to one of two final targets located 35 cm from the initial target and at 45° in the ipsilateral and contralateral directions relative to the initial target. At the preparatory cue, place the hand on the initial target; at the voice command, reach to the final target and return. Reaching performed with and without trunk restraint, 10 times each. | Reach velocity, time, and endpoint trajectory;  Trunk velocity, time, and trajectory;  Shoulder flexion and elbow extension angles;  Movement units. | Optotrak motion-analysis system with infrared LEDs on the sternum, shoulders, arm, forearm, and hand. | Lower reach velocity. Longer reach time and greater endpoint trajectory variability, influenced by target location and trunk use. Lower peak trunk velocity. Longer trunk movement time. Lower trunk ROM. Lower elbow extension ROM for the contralateral target. Greater number of movement units, influenced by trunk use. |
| Cirstea, Levin, 2000 | Position: Seated, initial target ipsilateral to the right arm on a platform (41.5 cm high) 10 cm lateral to the right hip.  Task: Reach to a contralateral final target 10 cm lateral and anterior to the left foot, at 80 cm height. Movement to be performed without vision, with eyes allowed to open for feedback every 5 trials. Forty trials total. | Movement velocity and time;  Trajectory distance;  Interjoint coordination;  Elbow extension, horizontal adduction and shoulder flexion, trunk displacement and rotation;  Movement units. | Optotrak system with infrared LEDs on the shoulders, sternum, arm, forearm, and hand. | Lower mean movement velocity. Longer movement time and trajectory. Impaired interjoint coordination: greater movement segmentation. Reduced ROM of elbow extension, horizontal adduction, and shoulder flexion. Increased trunk displacement and rotation. Greater number of movement units. |
| Michaelsen et al., 2001 | Position: Seated on an adjustable chair at 100% leg length, with 75% of the thigh supported; hand at the lower-third height, thumb 5 cm apart, elbow at the side.  Task: Reach and grasp a cone (7 cm diameter, 17.5 cm height) at two positions: (T1) at half arm length; (T2) at 100% arm length. Task performed with and without trunk restraint. Twenty repetitions per condition. | Movement time;  Endpoint and trunk trajectories;  Movement velocity;  Number of velocity peaks;  Maximal joint and trunk excursion;  Interjoint coordination;  Elbow extension, horizontal adduction, and shoulder flexion. | Optotrak system with 10 infrared LEDs on the shoulders, sternum, arm, forearm and hand, pelvis, and knee. | Lower movement velocity, especially with trunk restraint and for the farther target. Less smooth, more curved trajectory and greater trunk displacement. Reduced trunk displacement with restraint. Lower peak tangential velocity, especially with restraint and for the farther target. Lower elbow extension and shoulder horizontal adduction/flexion ROM and greater trunk displacement. Reduced elbow extension and shoulder flexion ROM with restraint. Impaired shoulder–elbow coordination; coordination improved with restraint. |
| Kusoffsky, Apel, Hirschfeld, 2001 | Position: Standing, arm at the side.  Task: Reach, grasp, and move a milk carton with 0.5 L of water placed on a table (72 cm height) at a distance equal to arm length, moving it to a lateral table (65 cm). Movement performed with both arms, one at a time. Five repetitions per arm. | Movement time;  Movement velocity. | ELITE system with two cameras. Ten reflective markers were placed, but only those on the wrists and the object were used for analysis. | Longer time in the reach and object-placement phases with the more affected hand. Shorter time in the object-lifting phase with the more affected hand. Lower mean movement velocity and higher peak velocity with the more affected hand. |
| Kamper et al., 2002 | Position: Seated, with the sternoclavicular notch aligned to the midpoint of the target array; hand resting on the body with the thumb against the navel; wrist immobilized with a splint; trunk restrained, allowing only scapular mobility.  Task: Perform 150 reaches: 75 with the more affected arm and 75 with the less affected arm. Each reach was to a different target within a 75-target matrix distributed across 5 latitudinal and 15 longitudinal meridians. | Reach fraction;  Path-length ratio;  Peak-velocity difference;  Number of velocity peaks;  Movement-direction variability. | Flock of Birds® sensor on the dorsum of the hand. | Reduced reach fraction with severity of impairment, and more limited for targets above and across the body. Higher path-length ratio. Lower peak velocity in the more affected arm. Greater number of velocity peaks. Greater movement-direction variability. |
| Levin et al., 2002 | Position: Seated without trunk support, hand near the sternum, elbow at the side.  Task: Reach, grasp, and return a cone (7 cm diameter, 17.5 cm height) positioned at sternal height to the start position. Reach at 4 distances: target 1 at half arm length; target 2 at 100% arm length; target 3 at 100% + one-third arm length; target 4 at twice arm length. Twenty trials per target. | Trajectory curvature;  Endpoint displacement;  Trunk and hand displacement and velocity;  Elbow extension;  Shoulder flexion and horizontal adduction. | Optotrak system with 9 infrared LEDs on the sternum, shoulders, arm, forearm and hand, pelvis, and knee. | Greater trajectory curvature for target 2. Greater trunk use for all targets, mainly targets 1 and 2. Higher trunk velocity for targets 1 and 2. Lower hand displacement for targets 2 and 4. Lower hand velocity for target 4. Lower elbow extension for targets 2 and 4. Lower shoulder flexion for target 4. |
| Cirstea et al., 2003 | Position: Seated, right hand on a platform (41.5 cm high), 10 cm ipsilateral to the right thigh.  Task: Perform 25 reaches with the right arm to a contralateral target positioned in front; target height and distance were adjusted to arm length and extension capacity. | Target distance from the sternum;  Target height;  Arm displacement;  Peak velocity;  Movement units;  Movement time;  Endpoint error;  Path-length index;  Trajectory variability;  Elbow extension and shoulder horizontal adduction;  Trunk displacement. | Optotrak system with infrared LEDs on the shoulders, arm, forearm, and hand. | Lower target distance from the sternum, arm displacement, and peak velocity. Greater number of movement units, longer total movement time, and greater endpoint error. Lower elbow extension and shoulder horizontal adduction. Greater trunk displacement and increased trajectory variability. |
| Roby-brami et al., 2003 | Position: Seated facing a wooden board, 15 cm from abdominal level.  Task: Reach and remove a cardboard cone (5 cm diameter, 17 cm height) fixed to the board. The board had 7 holes distributed over a 20 × 20 cm area, at distances 25–45 cm from the participant; holes 3–5 were beyond most participants’ arm length. Task performed with the more affected limb; those unable to lift the cone were to reach and return. Five repetitions per hole. | Peak velocity;  Movement duration;  AcROMion displacement;  Elbow extension;  Shoulder flexion, abduction, and rotation;  Trunk/scapular protraction and anterior tilt. | Fastrack™ Polhemus system (electROMagnetic), 4 sensors at 30 Hz on the acROMion, arm, forearm, and hand. | Lower peak velocity. Longer movement duration and greater acROMion displacement in those with “poor” motor function. Lower elbow extension and shoulder flexion, and greater shoulder abduction and anterior trunk tilt, mainly in those with “poor” function. |
| Roby-brami et al., 2003 | Position: Seated facing a wooden board, 15 cm from abdominal level.  Task: Reach and remove a cardboard cone (5 cm diameter, 17 cm height) fixed to the board. The board had 7 holes over a 20 × 20 cm area, at distances 12–32 cm from the participant. Task performed with the more affected limb. Five repetitions per hole. | Hand orientation (horizontal-plane deviation, elevation, rotation);  Wrist extension and adduction;  Supination;  Elbow extension;  Shoulder horizontal adduction, elevation, and internal rotation;  Scapular protraction;  Shoulder elevation and anterior trunk tilt;  AcROMion displacement. | Fastrack™ Polhemus system with 4 sensors (shoulder, arm, forearm, hand) at 30 Hz. | Greater hand orientation in the horizontal plane, especially with moderate impairment. Lower hand elevation and rotation and greater wrist extension and adduction with moderate impairment. Greater forearm supination and lower elbow extension and shoulder horizontal adduction with moderate stroke. Greater shoulder elevation and greater anterior trunk tilt with moderate stroke. |
| Reisman, Scholz, 2003 | Position: Seated with trunk supported and restrained; elbows at 90° flexion, shoulders slightly abducted; hand positioned in front of the corresponding shoulder.  Task: Place a pointer within a ring suspended at shoulder height and at 90% arm length, ipsilateral and contralateral. Twenty movements per target (ipsi/contra), first with right arm then left. | Movement time;  Shoulder and elbow mobility;  Variation of hand path length and direction;  Absolute mean pointing error. | VICON camera system; rigid retroreflective marker sets on the shoulder girdle, arm, forearm, and hand, attached to a splint. | Longer movement time. Lower shoulder and elbow ROM, mainly elbow extension. Greater hand-path variability with the more affected arm in middle, late, and terminal movement phases. Greater absolute pointing error with the more affected arm. |
| Michaelsen et al., 2004 | Position: Seated, hips and knees at 90° flexion, feet on the floor; more affected upper limb resting on a table; shoulder 10° extension and 20° abduction; elbow 70° flexion; forearm pronated; wrist neutral.  Task: Reach and grasp an object (3.5 cm diameter, 9.5 cm height) at two locations: T1 along the body midline at 90% arm length; T2 at the same distance but 45° lateral toward the ipsilateral side. Ten movements per target. | Wrist and elbow extension;  Shoulder horizontal adduction;  Trunk displacement;  Trunk rotation;  Hand orientation (horizontal-plane deviation, elevation, rotation);  Movement time;  Wrist peak velocity;  Time to wrist peak velocity;  Maximum hand aperture;  Time to maximum hand aperture. | Optotrak system with eight infrared LEDs on sternum, shoulders, arm, forearm, and hand; Fastrack Polhemus for 3D hand-orientation (rotational) data. | Lower wrist and elbow extension and shoulder horizontal adduction, mainly for T2. Greater anterior trunk displacement (mainly T1) and trunk rotation (T2). Greater horizontal-plane deviation. Lower hand elevation. Longer movement time. Lower peak velocity, mainly for T2. Longer time to maximum hand aperture. |
| Zackowski et al., 2004 | Position: Seated.  Task: Reaching to a suspended Styrofoam ball under two conditions: forward (≈40° shoulder flexion and elbow extension) and upward (≈40° shoulder and elbow flexion). Four to seven movements per condition. | Wrist peak velocity;  Endpoint error index;  Index-finger path ratio. | Optotrak with infrared LEDs on the shoulder, arm, forearm, and hand. | Higher wrist peak velocity. Imprecise reaching. Longer path relative to straight-line target distance. |
| McCrea, Eng, Hodgson, 2005 | Position: Seated, arm alongside the body, hand resting on the thigh.  Task: Reach and touch with the finger a 3 × 3 cm target at shoulder height and at an arm-length distance. Trunk and hip movements limited. Five movements recorded. | Elbow flexion/extension ROM;  Shoulder flexion/extension, abduction/adduction, and external/internal rotation ROM;  Hand trajectory;  Hand velocity. | Optoelectronic sensor (Northern Digital Optotrak) with sensors on the arm, forearm, and hand. | Elbow extension coupled with shoulder internal rotation for the more affected arm. Increased shoulder abduction and rotation. Shoulder abduction peak during elbow flexion. Increased segmentation and reduced trajectory directionality. Asymmetry and increased segmentation of hand-velocity profile. |
| Rose, Winstein, 2005 | Position: Seated, elbow at 90° flexion, forearm at 90° pronation.  Task: Reach and tap a button as fast as possible, each limb alone and then both simultaneously. Button located at body midline at a distance requiring ≤15° elbow extension and at least 90° shoulder flexion. Trunk movement restricted. Three separate blocks of 28 trials each. | Movement time;  Time to peak velocity;  Time after peak velocity;  Resultant peak velocity. | ElectROMagnetic motion capture and analysis. Hand displacement recorded from an electROMagnetic sensor on each hand. | Longer movement time, especially in the bimanual condition and for the less affected limb. Longer time to peak velocity in the bimanual condition. Longer post–peak-velocity time in the bimanual condition and in the less affected limb. Greater reduction in peak velocity for the less affected arm in the bimanual condition. |
| Wenzelburger et al., 2005 | Position: Seated with back support, hand on a table in front.  Task: Reach and grasp a socket 34 cm above the table and 50 cm in front of the trunk using index finger and thumb, release it, and return to start. Ten movements with the more affected limb. | Total movement time;  Acceleration time;  Deceleration time;  Time to grasp;  Total grasp duration;  Pre-shaping duration;  Pre-transfer duration;  Transfer duration. | Passive infrared motion-analysis system with reflective markers on the arm and hand. | Longer total movement time. Increased deceleration time and time to grasp. Increased total grasp duration and durations of pre-shaping and pre-transfer phases. |
| Kilbreath et al., 2006 | Position: Seated with support; trunk restrained; hand at the table edge.  Task: Reach and grasp a tray, move it to the table edge, and return to start. Unimanual and bimanual execution, five repetitions each. | Movement time (“hand to tray” and “tray transport”);  Total absolute hand deviation (cm/s);  Mean hand deviation (cm);  Synchrony of hand movement at movement onset;  Synchrony during handle release;  Relative phase angle. | Six key events signaled via switches under the wrist at start/end positions, on the tray handle, under the tray’s initial position, and at the table edge; electROMagnetic sensors on the wrists recorded absolute position relative to a fixed sensor. | Lower movement velocity in the hand-to-tray and tray-transport phases, mainly for the bimanual task with two small trays. Lower synchrony at movement initiation and at handle release. |
| Messier et al., 2006 | Position: Seated, shoulders at 25° abduction, wrists supported on a table.  Task: Move one or two cones placed at 100% arm length + 20 cm to one or two targets, respectively, and return to start. Movements in 3 directions: (1) in front; (2) 45° contralateral to the more affected limb; (3) 45° ipsilateral. Three trials per condition. | Shoulder flexion;  Shoulder abduction/adduction;  Elbow extension;  Anterior trunk flexion;  Lateral trunk flexion;  Trunk rotation. | Optotrak 3020 with 14 infrared markers on each upper limb and trunk. | Lower elbow extension, greater anterior trunk flexion, and greater trunk rotation. |
| Nowak et al., 2007 | Position: Seated, shoulder slightly abducted; elbow 90° flexion; forearm neutral; hand resting on a mark on a table.  Task: Reach and grasp an object 30 cm away, lift it 5 cm, hold for 3 s, and return to start. Ten movements with each hand. | Wrist vertical position and velocity peaks;  Wrist movement time;  Peak hand aperture;  Time to peak hand aperture. | Ultrasonic motion-analysis system (CMS 20S) with 3 microphones and ultrasonic-emitter markers on the forearm and hand. | Lower wrist elevation amplitude. Lower wrist velocity, mainly in the contralateral hand. Longer total wrist movement time. Greater hand aperture, mainly in the contralateral hand. Earlier peak hand aperture, mainly in the contralateral hand. |
| Van vliet, Sheridan, 2007 | Position: Seated on an adjustable chair, waist against a table; forearm neutral; elbow 100° flexion; wrist on a mark 20 cm from the cup.  Task: Reach and pick up a cup of water (small and large), drink, and return the cup to start. Movements at comfortable and fast speeds, 8 each. | Movement time;  Transport onset;  Peak velocity;  Time to peak velocity;  Time to peak deceleration;  Hand-opening onset;  Maximum hand aperture;  Time to maximum hand aperture. | MacReflex 3D motion system with two cameras, tracking markers on forearm and hand. | Longer movement time. Delayed transport onset. Lower peak velocity. Earlier times to peak velocity and to deceleration. Delayed hand-opening onset and time to maximum hand aperture. |
| Michaelsen, Magdalon, Levin, 2009 | Position: Seated, hips and knees at 90° flexion; forearm pronated; wrist neutral.  Task: Reach and grasp a small and a large cone, with two grasp types: whole-hand (all fingers flexed) and fingertip (finger pads only). Targets at body midline, at 2/3 arm length. Ten repetitions per condition. | Movement time;  Peak velocity;  Time to peak velocity;  Deceleration-phase duration;  Maximum grasp aperture;  Time to maximum grasp aperture;  Trunk displacement. | Optotrak 3010 optical system with infrared LEDs on the sternum, forearm, and hand. | Longer movement time. Lower peak velocity, but higher during whole-hand grasp. Longer time to peak velocity, mainly for whole-hand grasp. Prolonged deceleration phase. Longer time to maximum grasp aperture, occurring earlier when seizing smaller objects. Greater trunk displacement. |
| Sangole, Levin, 2009 | Position: Seated with back support; hand on an adjustable platform; shoulder 45° abduction; elbow 90° flexion; forearm neutral.  Task: Grasp a sphere and a cylinder and transport them 10 cm upward. Targets at 90% arm length, in front of the ipsilateral shoulder on a table, 5 cm above hand start. Ten repetitions per target. | Palmar-arch modulation in each phase;  Total movement time;  Time of each phase;  Alignment between MCP and PIP joints. | Vicon 3D motion system with 6 cameras; markers on forearm and hand. | Lower palmar-arch modulation during transport, contact-adjustment, and pre-shaping. Longer total task time. Longer durations of the three phases, especially for cylindrical grasp. Altered finger-coordination patterns, mainly during transport and pre-shaping for spherical grasp. |
| Raghavan et al., 2010 | Position: Seated at a table; right arm aligned with the body; elbow 90° flexion; hand on a fixed table position; instrumented glove used.  Task: Reach and grasp one of three objects (rectangular, concave, convex) using whole-hand grasp and lift from the table. Objects at 75% arm length. Seven repetitions per object. | Peak and ROM of abduction, flexion, and extension of MCP and PIP joints of digits 2–4;  Reach and grasp phases. | ElectROMagnetic position sensor (Polhemus, Colchester, VT) for wrist position; hand posture recorded via 14 sensors integrated in the glove. | Reduced finger abduction, PIP flexion, and MCP extension. Increased MCP flexion. Longer movement time. Hand shaping during the deceleration phase. |
| Murphy et al., 2011 | Position: Seated on a 46-cm chair at a 74-cm table; hand on the table with pronated arm; wrist line at the edge; shoulder adducted at side; elbow 90° flexion.  Task: Reach and pick up a cup (7 cm diameter, 9.5 cm height) 30 cm from the table edge and at body midline, lift it, take a sip of water, replace the cup on the mark, and return the hand to start. Five repetitions per arm. | Hand peak velocity;  Time to hand peak velocity;  First hand peak velocity;  Time to first hand peak velocity;  Elbow peak angular velocity;  Number of movement units;  Shoulder and elbow flexion/extension;  Shoulder abduction/adduction;  Maximal thorax displacement;  Interjoint coordination. | ProReflex motion capture with 5 optoelectronic cameras; data collected by Qualisys Track Manager 2.0 and analyzed in MATLAB. | Lower peak velocity and relative time to peak velocity, indicating longer deceleration phase. Longer time to reach peak velocity. Lower elbow peak angular velocity. Fewer movement units. Lower maximal elbow extension. Greater shoulder abduction during drinking. Greater trunk displacement. |
| Robertson, Roby-Brami, 2011 | Position: Seated with table at umbilical height; hand on the abdomen.  Task: Reach and touch nine plastic-disc targets (2 cm diameter) fixed to the table: three near (60% arm length), three far (90%), and three elevated (17 cm above far targets) across three directions relative to the shoulder: internal (−30°), central (0°), and external (30°). | Anterior, lateral flexion and trunk rotation;  Hand velocity;  Trunk velocity. | Polhemus Fastrak electROMagnetic tracker (6 DOF); sensors on sternum and dorsum of the hand. | Greater anterior flexion and trunk rotation. Lower hand velocity. Higher trunk velocity. |
| DeJong, Lang, 2012 | Position: Seated at a table with its edge at mid-thigh and adjusted as low as possible without touching the leg; hands resting on the lap.  Task: Grasp and lift a cylinder (34-mm diameter, 113-mm height, fixed to a rectangular base) at 90% arm length, aligned with the center of the clavicle; hold for 5 s and return to the table. Four grasp types: unilateral palmar, bilateral palmar, unilateral three-finger, bilateral three-finger. Three trials per condition. | Reach duration;  Pre-lift delay;  Release time;  Reach path-length ratio;  Peak hand aperture;  Hand-aperture path-length ratio. | ElectROMagnetic system (The MotionMonitor, Innovative Sports Training, Chicago, IL); 9 sensors on sternum, arm, forearm, and hand; reference sensor on the cylinder base. | Longer reach duration. Longer pre-lift delay for three-finger grasp. Higher reach path-length ratio. |
| Robertson, Roche, Roby-Brami, 2012 | Position: Seated at a table at umbilical height; trunk restrained; wrist immobilized with a splint bearing a pointer simulating an extended finger; hand on a mark; forearm neutral; elbow 90° flexion.  Task: Reach and touch targets at a comfortable speed. Targets were 1-cm-wide red tapes fixed to nine 1.5-cm-diameter rods in three directions: midline (toward the shoulder), internal (45° toward midline), and external (45° away). Six targets at 7 cm height—three at 90% arm length and three at 65%—plus three at acROMial height. | Hand peak velocity;  Curvature;  Accuracy;  Scapular protraction/retraction, rotation, and tilt ROM;  Glenohumeral elevation, abduction/adduction, and rotation;  Elbow flexion/extension. | Polhemus Fastrak (SPACE FASTRAK, Colchester, VT, USA); transmitters under the table; sensors on sternum, shoulder, arm, and on the dorsum of the wrist splint. | Lower hand peak velocity. Higher curvature index, mainly on the more affected side. Lower pointing accuracy. Lower scapular protraction, rotation, and tilt (mainly contralaterally). Greater glenohumeral elevation. Lower glenohumeral horizontal abduction and rotation. Reduced elbow extension contralaterally. |
| Schaefer et al., 2012 | Position: Seated, hands on the thigh, fingers together. Table edge at mid-thigh, adjusted as low as possible without touching the leg. Object at 90% arm length, aligned with the midpoint of the more affected clavicle.  Task: Reach, grasp, and hold or lift a cylindrical object (circumference 10.7 cm; diameter 3.4 cm; height 11.3 cm) with a rectangular base (13 × 6 cm), weight 420 g. Two grasp types: three-finger and palmar. Four conditions: hold only; hold and lift; each with both grasp types. | Reach path-length ratio;  Peak reach velocity;  Reach time;  Contact velocity. | ElectROMagnetic tracking with nine sensors (The MotionMonitor) on the sternum, arm, forearm, hand, and on the nail of each finger. | Higher reach path-length ratio. Lower peak reach velocity. Longer reach time with three-finger grasp. Longer time for the “lift” task. |
| Van Kordelaar, van Wegen, Kwakkel, 2012 | Position: Seated at a 76-cm table; hand at table edge in front of the shoulder. Object at maximal arm length; target on the contralateral side at the same distance.  Task: Reach, grasp, lift, and move the object to the target. | Movement time;  Trunk anterior, lateral, and axial rotation ROM;  Shoulder upward, horizontal, and internal rotation ROM;  Elbow flexion ROM;  Forearm pronation ROM. | Polhemus Liberty (6 DOF) electROMagnetic tracker; sensors on thorax, scapula, arm, and forearm. | Higher mean movement time. Greater elbow flexion. |
| Merdler et al., 2013 | Position: Seated, feet on the floor; table at elbow height. Three targets (30 × 30 mm) beyond arm length: central at eye level (150 mm above shoulder); contralateral 300 mm left of central; ipsilateral 300 mm right.  Task: Reach to targets in randomized order. Two blocks of 7 movements per target. | Arm-plane angle;  Peak velocity;  Time to peak;  Trajectory length and variation;  Curvature index;  Number of velocity peaks;  Elbow flexion/extension ROM;  Shoulder flexion/extension and horizontal abduction/adduction ROM;  Trunk trajectory length anteriorly, laterally, and rotationally. | Optoelectronic system with three cameras (ProReflex, Qualisys Ltd., Sweden); seven passive reflective markers on shoulder, arm, forearm, hand, and on a back-mounted exoskeleton to record trunk motion. | Greater arm-plane angle (compensatory elbow elevation). Reduced arm-plane velocity, mainly for ipsilateral targets. Shorter time to peak velocity and shorter trajectory length for contralateral targets. Less straight trajectory and more velocity peaks. Lower elbow extension, greater shoulder abduction, and lower shoulder adduction. Greater trunk displacement in all directions, especially for contralateral targets. |
| Aprile et al., 2014 | Position: Seated with hands on a table. Cup positioned 400 mm from the table edge, aligned with the participant’s sagittal plane.  Task: Reach, grasp, bring the cup to the mouth, drink, and reposition the cup on the table. | Phase durations;  Arm elongation;  Anterior trunk inclination;  Trunk rotation;  Elbow flexion/extension ROM;  Anterior mouth displacement;  Arm contribution;  Number of movement units. | SMART optoelectronic capture system (BTS S.p.A., Milan), 30 markers on head, shoulders, arm, forearm, hand, and trunk; eight cameras. | Longer execution time for each phase. Less arm elongation. Reduced elbow flexion/extension ROM and trunk rotation. Increased anterior trunk inclination, anterior mouth displacement, and number of movement units. Greater variability during the bring-to-mouth phase. |
| Shaikh et al., 2014 | Position: Seated at a table; hand 30 cm from the sternum on the sagittal midline; shutter goggles used to avoid visual corrections. Target at 1.3× arm length, at 45° from the sagittal line.  Task: Sixty reaches, 70% without trunk restraint and 30% with unexpected trunk restraint via an electROMagnet attached to the back. | Hand peak velocity;  Trunk peak velocity;  Time to hand peak velocity;  Time to trunk peak velocity;  Hand displacement;  Trunk displacement;  Trunk rotation ROM;  Elbow extension ROM;  Shoulder flexion and horizontal adduction ROM. | 3D motion analysis (Optotrak 3010, Northern Digital, Waterloo) with six infrared LEDs on sternum, shoulders, arm, forearm, and hand. | Lower hand and trunk peak velocities. Longer time to peak velocity. Lower hand displacement, elbow extension, and shoulder flexion. |
| Stewart, Gordon, Winstein, 2014 | Position: Interaction with an immersive virtual panel; cursor positioned over a 2.5 cm blue sphere. Six targets presented in two directions (+45° and −45°) and three distances (8, 16, 24 cm).  Task: Reach to whichever of the 6 targets appeared. Movement first with the more affected limb, then the less affected. 168 trials in 7 blocks of 24. | Movement time;  Movement distance;  Endpoint error;  Peak velocity;  Peak acceleration;  Time to peak velocity. | ElectROMagnetic marker on the reaching hand serving as the main interface to the virtual environment and for position data capture; stereoscopic glasses calibrated at 60 Hz per eye for 3D viewing. | Longer movement time in the more affected arm. Greater variability in movement distance. Greater endpoint error in the more affected arm. Lower peak velocity and acceleration in the more affected arm. Longer time to peak velocity in the more affected arm. |
| Levin et al., 2016 | Position: Seated, hands resting on a 100 × 150 mm platform on a table adjusted to elbow height. Three suspended targets (30 × 30 mm): the second aligned with the more affected shoulder at eye height at arm length; the first and third 150 mm below the second and 300 mm to the left and right, respectively.  Task: Point to the targets without touching them. Seven movements per target. | Lateral trunk displacement;  Sagittal trunk displacement;  Trunk rotation;  Elbow extension;  Arm-plane angle;  Final error. | Optoelectronic system with three cameras (7s, 100 Hz, ProReflex, Qualisys, Gothenburg); three markers on shoulder, arm, and forearm; trunk displacement via three orthogonal markers on a back exoskeleton. | Greater lateral and anterior trunk displacement in those with moderate/severe impairment. Greater trunk rotation. Lower elbow extension, especially for ipsilateral targets and in moderate/severe cases. Greater arm-plane angle for all targets and higher final error for the central target, mainly in the moderate/severe group. |
| Alvarez et al., 2017 | Position: Seated, facing a 48-cm and a 70-cm-high table. Four objects (water bottle, teaspoon, tissue pack, tennis ball) placed at 30% arm length.  Task: Grasp the object as if to use it. Three trials per object. | Grasp type used. | Three cameras around the object triggered simultaneously via open-source iSPY software; videos analyzed and re-rated by two expert physiotherapists. | Four grasps identified: digito-palmar (palm against one or more fingers); raking (fingers without thumb); ulnar (ulnar side of hand, involving ring and little fingers); interdigital (object supported between fingers without palm). |
| Ma et al., 2017 | Position: Seated on adjustable chairs; knees flexed and ankles dorsiflexed at 90°; hands resting on the table edge. Two bells aligned with the shoulders at two distances: 90% and 125% arm length.  Task: Perform bilateral reaches to touch the bells as fast as possible. Three recorded attempts. | Shoulder flexion and abduction;  Elbow extension;  Trunk flexion, rotation, and lateral displacement;  Arm–trunk interaction. | VICON (7 cameras) with optical markers on anterior/posterior trunk, shoulder, arm, forearm, and hand. | Lower elbow extension. Greater trunk flexion for within-arm-reach targets. Greater trunk rotation and lateral displacement toward the more affected side for beyond-reach targets. Arm–trunk incoordination: trunk movement from reach onset. |
| Valdes, Glegg, Van der Loos, 2017 | Position: Seated; hips and knees at 90°; 75% of thighs supported; hand on the xiphoid process. Four haptic robot–presented targets: A and B at 90% and 50% arm length at xiphoid height; C and D at 90% arm length at shoulder and knee heights.  Task: Perform bimanual reaches to presented targets. Fifteen movements per target. | Anterior trunk displacement;  Curvature index;  Movement time. | Two haptic robotic devices (Geomagic Phantom Premium 1.5) and a motion-tracking camera (Microsoft Kinect v1). Movement-analysis data obtained via Kinect. | Greater anterior trunk displacement. Greater inter-hand asymmetry. Higher coefficient of variation of hand movements. |
| Tomita, Mullick, Levin, 2018 | Position: Standing, base of support shoulder-width apart; eyes closed; safety harness to ceiling; hand on the superior border of the ipsilateral greater trochanter. Target at 130% arm length on the sagittal midline at the anterior–superior iliac spine height.  Task: Touch the target. In 30% of movements, an electROMagnetic device attached to a back plate remained engaged, blocking hip motion; in 70%, it disengaged, allowing hip motion. Sixty-five movements total. | Peak velocity;  Curvature index;  Number of movement units;  Final elbow, shoulder, and hip flexion angles;  Step initiation;  Step length. | 3D motion capture (Optotrak, NDI, Canada) with IR-LED markers on sternum, shoulders, arm, forearm, hand, pelvis, knee, and ankle. | More curved trajectory. Greater number of movement units, mainly with hip blocking. Multiple velocity peaks in the moderate group. Less variation in elbow, shoulder, and hip flexion when the hip was blocked or when an intentional step occurred. Longer step-initiation time. Greater variability in step length. |
| Thrane et al., 2019 | Position: Seated on an adjustable chair, hips and knees at 90°, at an adjustable table; test-hand on the table. Cup at 30 cm from the table edge.  Task: Reach, grasp, lift a cup (7 cm diameter, 9 cm height, 100 ml water), drink, and return it to start. Five movements per arm. | Total movement time;  Number of movement units;  Peak velocity;  Time to peak velocity;  Elbow peak angular velocity;  Maximal elbow extension angle;  Shoulder abduction during drinking;  Trunk displacement. | ProReflex optoelectronic system with five cameras (MCU240 Hz, Qualisys AB, Sweden); nine markers on head, sternum, shoulders, arm, forearm, hand, and on the top/bottom edges of the cup. | Slower movement, more movement units, reduced peak velocity and elbow angular velocity, and greater shoulder abduction and trunk displacement in those with FMA-UE > 60. Submaximal FMA-UE group showed longer movement time, more movement units, and lower linear and angular elbow velocities than the maximal group. Both maximal and submaximal groups showed greater shoulder abduction and greater trunk displacement. |
| Feingold-Polak et al., 2021 | Position: Seated without trunk support at a table adjusted to three heights: low (50 cm), medium (75 cm), and high (shoulder height). Cup aligned horizontally with the test limb, at arm length.  Task: Reach a cup (two weights: empty and filled with water), grasp it, and place it on a 5-cm block. Six movement conditions (height × weight). | Movement time;  Mean velocity;  Peak velocity;  Time to peak velocity;  Curvature index;  Trunk displacement;  Scapular elevation and rotation;  Elbow movement. | Portable V120:Trio (OptiTrack) motion capture with three cameras; 11 markers on sternum, shoulder, arm, forearm, and hand; two wall reference markers and three on the cup. | Longer movement time. Lower mean velocity during reach and lift phases. Lower reach peak velocity and longer time to peak. Higher curvature index. Greater trunk displacement and greater scapular elevation for higher targets. Greater scapular rotation during reach for lower targets and during grasp for higher targets. Lower elbow extension during reach and lift. |
| Padilla-Magaña et al., 2022 | Position: Seated on a chair with backrest, at a table 15 cm away and adjusted to abdominal height.  Task: Sixteen ARAT tests were administered. For each, participants were to grasp the object, lift it, place it on the table, and release it. | Flexion angles of joints:  CMC, MCP, and IP of the thumb;  MCP and PIP of digits 2–5.  ROM of flexion, extension, and arc of motion of joints:  CMC, MCP, and IP of the thumb;  MCP and PIP of digits 2–5. | CyberGlove II® during the 16 activities; 18 sensors: two per finger, four abduction sensors, and sensors measuring thumb opposition, palmar arch, wrist flexion, and wrist abduction. | Greater flexion of thumb MCP and IP joints. Lower MCP flexion and greater PIP flexion of digits 2–5. Greater MCP extension angle and increased arc of motion at the PIP of all digits. |
| Choi et al., 2023 | Position: Seated without trunk support; shoulders neutral; elbows 90° flexion; forearms pronated; wrist neutral. Object 30 cm from body midline. Task: Reach and grasp (P1) a cup (12.5 cm height, 7 cm diameter), bring it to the mouth and drink (P2), place it back on the table (P3), and return to start (P4). | Movement time per phase (P1–P4) and total;  Peak velocity per phase;  Number of movement units per phase and total;  ROM per phase of:  shoulder flexion, abduction, internal rotation;  elbow flexion;  wrist extension, pronation, ulnar deviation;  thorax anterior tilt, internal rotation, and superior obliquity. | VICON MX (six cameras) with eleven markers on the spine, shoulders, sternum, arm, forearm, and hand. | Longer movement time, especially in reach, bring-to-mouth, and return phases. Reduced peak velocity. Greater number of movement units. Reduced elbow and wrist flexion/extension. Compensatory increase in shoulder abduction and rotation. Greater thorax mobility, mainly in the bring-to-mouth phase. |
| Ota et al., 2023 | Position: Seated, hand on a table at elbow height. Object at one-third arm length. Task: Reach for a 5-cm-diameter, 8-cm-long, 50-g object and transport it from start to maximal arm extension; and from start to the mouth. | Total anterior reach distance; Vertical hand-to-mouth distance; Shoulder elevation, abduction, and flexion ROM; Elbow extension ROM; Trunk flexion and rotation ROM; Cervical flexion ROM. | KinemaTracer (Kissei Comtec Corporation): computer with analysis software and six cameras; ten anatomical markers on mandible, cervical spine, shoulder, arm, hand, and thigh. | Lower shoulder and elbow flexion contributions to total anterior reach distance; greater trunk flexion contribution. Lower shoulder and elbow flexion contributions to vertical hand-to-mouth distance; greater contribution of shoulder-girdle elevation and shoulder abduction. Greater shoulder elevation/abduction ROM and trunk flexion. Lower shoulder flexion and elbow extension ROM. |

3D: Three-dimensional; ARAT: Action Research Arm Test; CMC: Carpometacarpal; DOF: Degrees of freedom; FMA-UE: Fugl-Meyer Assessment – Upper Extremity; IP: Interphalangeal; IR: Infrared; MCP: metacarpophalangeal; PIP: Proximal interphalangeal; ROM: Range of motion; USA: United States of America.
